# Commercial suitability and characterization of newly developed *Erucastrum canariense* (*Can*) sterile cytoplasm based cytoplasmic male sterile (CMS) lines in Indian cauliflower

**DOI:** 10.1038/s41598-024-52714-z

**Published:** 2024-01-29

**Authors:** K. S. Manjunath, Shrawan Singh, Pritam Kalia, Manisha Mangal, Brij Bihari Sharma, Naveen Singh, Mrinmoy Ray, Mahesh Rao, Bhoopal Singh Tomar

**Affiliations:** 1 Division of Vegetable Science, ICAR-Indian Agricultural Research Institute, New Delhi, 110012 India; 2 Division of Genetics, ICAR-Indian Agricultural Research Institute, New Delhi, 110012 India; 3 ICAR-Indian Agricultural Statistical Research Institute, New Delhi, 110012 India; 4 ICAR-National Institute of Plant Biotechnology, New Delhi, 110012 India

**Keywords:** Plant breeding, Plant genetics

## Abstract

The study presents the first to characterize novel *Erucastrum canarianse* Webb and Berthel (or *Can*) sterile cytoplasm-based CMS lines in Indian cauliflower (*Brassica oleracea* var. *botrytis* L.) and investigating their commercial suitability. Eleven *Can*-based CMS lines were examined for 12 agro-morphological and yield traits,18 floral traits, four seed yield traits together with three each of the *Ogura* (source: wild Japanese Radish) and *Tour* (Source: *Brassica tournefortii*) cytoplasms. All of the recorded floral and seed traits showed significant (*P* > 0.05) differences between the CMS lines of each group. Agro-morphological and yield traits in CMS lines and their maintainers, however, were non-significantly different. All the *Can-* and *Ogura-*based CMS lines showed flowering and appropriate seed formation by natural cross-pollination. Only two *Tour* cytoplasm-based CMS lines, *Tour* (DC-41-5) and *Tour* (DC-67), produced the smallest malformed flowers and stigma. The highest seed yield per plant in CMS lines was in *Ogu* (DC-98-4) and the lowest in *Tour* (DC-67). P14 and P15, two polymorphic mtDNA markers, were discovered for the *Can* CMS system for early detection. Five primers (ITS5a-ITS4, atpF-atpH, P16, rbeL and trnL), along with their maintainers, were sequenced and aligned to detect nucleotide changes including as additions and or deletions at different positions. The newly introduced *E*. *canariense* sterile cytoplasm-based CMS system in cauliflower is the subject of the first comprehensive report, which emphasises their potential as a further stable and reliable genetic mechanism for hybrid breeding.

## Introduction

Cytoplasmic male sterility (CMS) is a robust, easy-to-maintain and produces satisfactory hybrid seed yield which are essential features for a genetic mechanism to use in commercial hybrid seed production. It is the maternal inheritance of the inability of a plant to produce a viable pollen grain and is governed by mitochondrial genome^1,2^. Introgression of sterile cytoplasm from different species or genus creates new nulceo-cytoplasmic interactions in the converted CMS lines. However, the level of impact needs to be investigated which may vary depending upon donor source (species or genus) and recipient backgrounds (different genotypes).

There are different male sterile cytoplasms available for *Brassica* crops, namely ‘*Ogu’* from wild Japanese radish^3^,‘*Can*’ of *Erucastrum canariense* Webb and Berthel^4^*,* ‘*Tour*’ of *Brassica tournefortii* Goun^5^, *nap’* of *Brassica napus* L.^6^ ,‘*kosena*’ of ‘*Raphanus sativus* L.^7^ , ‘*mur*’ of *Diplotaxis muralis* (L.) DC^8^ , ‘*Pol’* of *B. napus*^9^,‘*hau*’ of *Brassica juncea* (L.) Czern^10^ ,‘*mori*’ of *Moricandia arvensis* L.^11^ *,‘fruti’* of *Brassica fruticulosa* Cirillo^12^ ,‘*inap’* of *Brassica napus* L. + *Isatis indigotica* Fort.^13^ and *Tour-Stiewe* of *B. tournefortii* Goun (somatic cell fusion)^14^. The present study elaborates on alloplasmic CMS lines developed using two sets of combinations: first, two species of the same genus *i.e.* cytoplasm of *Brassica tournefortii* (*Tour* cytoplasm) and nuclear genome of cauliflower (*Brassica oleracea* var. *botrytis* L.) and second, two different genera either ‘*Raphanus sativus* L.’ (*Ogura* cytoplasm) or *E. canariense* (*Can* cytoplasm) and nuclear genome of cauliflower. The *Ogura* cytoplasm is the only commercially exploited male sterility in *Brassica oleracea* L. for hybrid breeding.

Cauliflower (*Brassica oleracea* var*. botrytis* L.; 2n = 2x = 18) is one of the most important vegetable crops worldwide. It is being grown in an area of 1.36 million ha with production of 25.53 million tonnes^15^. With a contribution of around 33% in area and 34.5% in global production, India ranks second behind China. In India, area and production of cauliflower are 0.473 million ha and 9.22 million tonnes, respectively^16^. Cauliflower is being grown for white curds which have unique texture, flavour and taste. Fresh curd portion has 1.9% protein, 5% carbohydrate, 95% water, 48.2 mg vitamin C, and 199 mg potassium^17^. It contains health-beneficial glucosinolates particularly sinigrin, glucobrassicin and 4 hydroxy-glucobrassicin^18^.

Cauliflower originated in the Island of Cyprus and travelled to Europe via trade routes. The European type varieties of cauliflower were first introduced to the Indian sub-continent in 1822 at Company Bagh, Saharanpur in United Province (currently Uttar Pradesh, India) by a British botanist named Dr. Jemson^19^. The newly introduced cauliflower could only be grown in winter due to India’s higher temperature during summer and rainy seasons^20^. However, with staggered planting, human and natural selection pressure and crossing-recombination occurrences, the introduced cauliflowers evolved into ‘local types’ that were better adapted to high temperature and humidity. It formed curd at higher temperature (20–27 °C) than the source European type varieties (10–16 °C or less). The ‘local’ or ‘Indian’ type cauliflower produced satisfactory seeds with a lesser requirement of cool temperature which was prevalent during winters in North Indian plains^19,21,22^. Based on temperature requirement for curd formation, the Indian cauliflower is grouped into Early (20–27 °C), Mid-early (16–20 °C) and Mid-late (12–16 °C) maturity groups^23^. Mid-September to mid-November, mid-November to mid-December and mid-December to mid-January, respectively are the contiguous seasons for these temperatures. Late or snowball cauliflower (10–16 °C) is also common for harvesting during February–March in North Indian plains; nevertheless, due to temperature considerations, its seed production is feasible in the middle to higher altitude mountainous regions^24,25^.

The F_1_ hybrids have been crucial in increasing yield of cauliflower while also improving crop uniformity and product quality, both of which attracted farmers and retailers. The F_1_ hybrids are advantageous over the open-pollinated varieties (OPV) for uniformity, productivity, quality and tolerance to various biotic and abiotic stresses^26^. Therefore, the primary breeding goal for cauliflower is to develop F_1_ hybrids using genetic mechanisms for economic hybrid seed production. The earlier efforts were confined to self-incompatibility (SI) for hybrid development due to lack of alternative usable genetic mechanisms. In India, the SI system has been utilized for breeding three hybrids namely Pusa Hybrid-2 (1998), Pusa Kartik Sankar (2004) and Pusa Cauliflower Hybrid-101 (2021). However, the SI system has issues of poor stability at higher temperature, sib seeds and cumbersome maintenance^26,27^. The breeders tried for alternative genetic mechanism(s) with robust phenotype and easy maintenance. Genic male sterility (GMS) was identified in *Brassica oleracea* crops such as cauliflower (*ms-4*, *ms-5*) and other Cole vegetables namely *ms-1*^28,29^, *ms-2*, *ms-4*, *ms-5* and *ms-6*^30^, DGMs79-399-3^31^ in broccoli and *Ms-cd1* in cabbage^32^, but unfit for commercial use in hybrid breeding^25^. The identification of *Ogura* sterile cytoplasm in wild Japanese radish^3^, its deployment in *Brassica oleracea* L.^33^ and refinements for chlorosis defects^34^ has opened a new era of hybrid breeding. The cytoplasmic male sterility (CMS) owing to its robustness and easy maintenance was deployed in different nuclear backgrounds of cauliflower including genotypes from all the maturity groups^22^. The *Ogura* system imparts complete male sterility which facilitated its use in commercial hybrid seed production. *E. canariense* (*Can* system) and *B. tournefortii* (*Tour* system) are two other sterile cytoplasms deployed in cauliflower^4,35^. Cauliflower breeders at ICAR-IARI, New Delhi transferred these three CMS systems in different genetic backgrounds in cauliflower^36^. Singh et al.^22^ studied the *Ogura*, *Can* and *Tour* CMS cytoplasm deployed CMS lines in Indian cauliflower for floral and yield traits and indicated for better prospect of *Ogura-* and *Can-* based CMS systems in hybrid breeding. They reported that *Tour* cytoplasm-based CMS lines of cauliflower have ‘gripping phenomena’ wherein stigma gets trapped in a sepal which hinders insect pollination. *Ogura* cytoplasm has been well investigated for its interaction with different genetic backgrounds^22,25,37,38^ and for their heterotic potential^7,39,40^. It impact floral traits in snowball cauliflower^41^ and Indian cauliflower^22^. The *Can* cytoplasm is the most recently deployed sterile cytoplasm in different genotypes of the early group of Indian cauliflower at ICAR-IARI, New Delhi. These *Can*-based CMS lines need to be investigated for the impact of *Can* cytoplasm on agro-morphological and floral traits in nuclear backgrounds of cauliflower genotypes and also their potential in F_1_ hybrid seed production.

## Materials and methods

### Plant material and morphological observations

Seventeen CMS lines (11 *Can* CMS; 3 *Ogura* CMS; 3 *Tour* CMS) and their 11 maintainers were developed and being maintained by Division of Vegetable Science, IARI, New Delhi (Table 1). The original introgression *Can* CMS line ‘NIPB-1’ was developed and maintained by ICAR-NIPB, New Delhi and maintainer ‘PM’ by ICAR-IARI, New Delhi. Three maintainers namely DC 98-4, DC-41-5 and DC-67 were kept common for all three sterile cytoplasms (*i.e. Can*, *Ogura* and *Tour*) carrying CMS lines. The experiment was carried out at Vegetable Research Farm, Division of Vegetable Science, ICAR-IARI, New Delhi (28.08 ^0^N and 77.12 ^0^E, above mean sea level being 228.61 m). The seedlings were raised on nursery beds of 3 m × 0.5 m × 0.20 m size. Sowing was done on 20.07.2021 and transplanted on 25.08.2021 at 45 × 45 cm spacing in complete randomized block design (RBD) with 3 replications. Standard crop management practices and plant protection measures were adopted for raising the trial crop^23^.

**Table 1:** List of *Can*, *Ogura* and *Tour* CMS lines and their maintainers used for study on agro-morphological and molecular characterization of Indian cauliflower.

CMS lines/maintainers	Nuclear genome source	Protoplast source	Mitochondrial genome source	Generation with cauliflower	Generations with maintainer	Maturity group
CMS lines						
*Can* (DC-23)	Cauliflower	*E.* *canariense*	*E.* *canariense*	BC_11_	BC_5_	Early
*Can* (DC-98-4)	Cauliflower	*E.* *canariense*	*E.* *canariense*	BC_11_	BC_5_	Early
*Can* (DC-7)	Cauliflower	*E.* *canariense*	*E.* *canariense*	BC_11_	BC_5_	Early
*Can* (DC-8)	Cauliflower	*E.* *canariense*	*E.* *canariense*	BC_11_	BC_5_	Early
*Can* (DC-94-2)	Cauliflower	*E.* *canariense*	*E.* *canariense*	BC_11_	BC_5_	Early
*Can* (DC-903)	Cauliflower	*E.* *canariense*	*E.* *canariense*	BC_11_	BC_5_	Early
*Can* (DC-209)	Cauliflower	*E.* *canariense*	*E.* *canariense*	BC_11_	BC_5_	Early
*Can* (DC-41-5)	Cauliflower	*E.* *canariense*	*E.* *canariense*	BC_11_	BC_5_	Early
*Can* (DC-18)	Cauliflower	*E.* *canariense*	*E.* *canariense*	BC_11_	BC_5_	Early
*Can* (DC-63)	Cauliflower	*E.* *canariense*	*E.* *canariense*	BC_11_	BC_5_	Early
*Can* (DC-67)	Cauliflower	*E.* *canariense*	*E.* *canariense*	BC_11_	BC_5_	Early
*Ogu*(DC-98-4)	Cauliflower	Cauliflower	*Ogura* (wild Japanese radish)	*	BC_15_	Early
*Ogu*(DC-41-5)	Cauliflower	Cauliflower	*’’*	*	BC_15_	Early
*Ogu*(DC-67)	Cauliflower	Cauliflower	*’’*	*	BC_15_	Early
*Tour* (DC-98-4)	Cauliflower	Cauliflower	*Brassica* *tournifortii*	*	BC_8_	Early
*Tour* (DC-41-5)	Cauliflower	Cauliflower	*’’*	*	BC_8_	Early
*Tour* (DC-67)	Cauliflower	Cauliflower	*’’*	*	BC_8_	Early
Fertile inbred lines						
DC-23	Cauliflower	Cauliflower	Cauliflower	Inbred	Inbred	Early
DC-98-4	Cauliflower	Cauliflower	Cauliflower	Inbred	Inbred	Early
DC-7	Cauliflower	Cauliflower	Cauliflower	Inbred	Inbred	Early
DC-8	Cauliflower	Cauliflower	Cauliflower	Inbred	Inbred	Early
DC-94-2	Cauliflower	Cauliflower	Cauliflower	Inbred	Inbred	Early
DC-903	Cauliflower	Cauliflower	Cauliflower	Inbred	Inbred	Early
DC-209	Cauliflower	Cauliflower	Cauliflower	Inbred	Inbred	Early
DC-41-5	Cauliflower	Cauliflower	Cauliflower	Inbred	Inbred	Early
DC-18	Cauliflower	Cauliflower	Cauliflower	Inbred	Inbred	Early
DC-63	Cauliflower	Cauliflower	Cauliflower	Inbred	Inbred	Early
DC-67	Cauliflower	Cauliflower	Cauliflower	Inbred	Inbred	Early

The CMS lines and their maintainers were observed for 12 agro-morphological and curd yield traits, 18 floral traits and four seed yield traits. Plant height (PH), plant spread (PS), leaf length (LL), leaf width (LW), number of leaves per plants (NLP), gross plant weight (GPW), marketable curd weight (MCW), net curd weight (NCW), root length (RL) and root width (RW) were recorded from five random plants in each plot at curd maturity or harvesting stage. Marketable curd yield (MCY, t/ha) was calculated from plot yield. The harvest index (HI) was obtained by using the flowing formula: HI = MCW (g)/GPW (g).

The 18 floral traits were recorded each of 10 random plants at the flowering stage in CMS lines and their maintainers grown in replicated trials with three replications. The observations were taken using 25 to 30 fully developed floral buds (for floral bud traits) or naturally opened flowers (for flower traits) from each plant. Flower length (FL), flower diameter (FD), stalk length (StaL), petal length (PL), petal width (PW), sepal length (SL), sepal width (SW), bud length (BL) and bud width (BW) were recorded using standard rural scale. Stigma length (StiL), long stamen length (LSL), short stamen length (SSL), long filament length (LFL), short filament length (SFL), anther length (AL), nectary length (NL) and nectary width (NW) were taken using Discovery.18 SteREO Zoom Microscope (Carl Zeiss Microimaging Gmbh, Germany) in Division of Floriculture and Landscaping, IARI, New Delhi. Fully opened flowers from already covered inflorescence branches were taken and nectar was collected in calibrated capillary tubes (Sigma-Aldrich, Missouri, USA). The nectar volume (NV, μl/10 flowers) was calculated by dividing the length of the nectar column in the capillary tubes by the total length of the capillary tube^42^.

For seed parameters, all the 17 CMS lines were grown alongwith fertile pollen-parent in 1:1 ratio in three replications at two isolation blocks namely Mela Gate Farm (MG) and Vegetable Research Farm (VRF) located ≈1000 m apart in ICAR-IARI, New Delhi. Pollen parent DC-67 and DC-98-4 were planted at MG and VRF site, respectively. The seed parameters namely siliqua length (SqL), number of seeds (NS) per siliqua, seed yield (SY) per plant and 1000-seed weight or test weight (TW) were recorded from five randomly tagged plants.

### DNA extraction and PCR analysis

The DNA of CMS lines and their maintainers was extracted from fresh mature leaf samples from healthy young plants by using CTAB protocol^43^ and stored at −80 °C for further molecular work. The sampling for DNA extraction was done from five plants of each genotype (CMS and maintainers). The DNA quality was checked using agarose gel and DNA concentration was measured using Nanodrop (Eppendorf). The DNA was diluted with 0.1 X TE buffer (10 mM Tris, 0.1 mM EDTA, pH 8.0) to yield a working concentration of 30 ng/μl. An equal volume of this working DNA sample from each of the five plants was mixed and made a representative sample for the respective genotype.

A set of 25 mt-DNA primers available in the public domain were synthesised by Integrated DNA Technologies, (IDT, Coraville, Iowa, USA) and screened for amplification using polymerase chain reaction (PCR) (Biometra Tone 96G, Analytik Jena, Germany). The details of the primers and their sequence information are given in Table S1. These were synthesized from Integrated DNA Technologies, Inc (IDT, USA). All the primers were amplified using 10 μl PCR mixture volume with 2X Master mix (GeneDireX® OnePCR™, Forward and Reverse Primer 1.0 μL (0.5 μL each, 25 μM), Template DNA (1.0 μL, 30 ng/μL) and Sterile double distilled water (3.0 μL, 75 ng/μL) using thermocycler. The PCR steps were: Initial denaturation at 94 °C for 5 min followed by 35 cycles of denaturation at 94 °C for 1 min, annealing at 55–65 °C for 1 min (varied with primer), extension at 72 °C for 1 min; final extension at 72 °C for 10 min and hold at 4 °C for 30 min followed by storing at 4 °C for further processing.

### Electrophoresis and fragment detection

The amplified PCR products were resolved on 3% agarose gel in 100 mL 1X TAE buffer using 1 μg/mL of ethidium bromide florescent dye in gel electrophoresis (BioRad, USA). DNA bands were visualized and photographed under UV light in gel documentation unit (Alpha imager Cell Biosciences, Santa Clara, CA). The molecular markers showing variation in banding pattern among the genotypes were considered as polymorphic while rest were counted as monomorphic. Amplicon size was measured using the DNA ladder (GeneDireX Inc., 50 bp) as a reference.

### Nucleotide sequencing

The PCR products were obtained from selected eight genotypes representing three different sterile cytoplasms namely *Can*, *Ogura* and *Tour*. These included five CMS lines, namely *Can *(DC-23), *Can *(DC-98-4), *Can *(DC-41-5), *Ogu*(DC-41-5) and *Tour* (DC-41-5) and three maintainers, namely DC-23, DC-98-4 and DC-41-5. The PCR products from five selected primers (i.e., ITS5a-ITS4, atpF-atpH, trnL, P16 and rbeL) were custom sequenced at Agri-Genome Pvt. Ltd., Hyderabad. The forward and reverse sequences, thus, obtained were assembled to obtain contigs using Bioedit sequence alignment editor version 7.0.5.3. The contigs were aligned using ClustalW multiple alignment option of BioEdit software and output was obtained under graphic view and exported as rich text with 50 residues per row and in blocks of ten residues.

### Statistical analysis

The data from agro-morphological, floral and seed yielding traits were recorded and subjected "doebioresearch" package of R software for ANOVA (analysis of variance). Standard deviation and per cent change due to CMS introgression were calculated over the fertile (maintainer) line for agro-moprhological and floral traits using Microsoft Excel 2019. K-means clustering was implemented using “cluster” package of R software. The optimal number of clusters was determined by average silhouette width. Average silhouette width was computed employing “factoextra” package of R software. The "Hmisc" R package was utilized to perform correlation analysis on both agro-morphological and floral traits. Non-significant correlations, determined at a significance level of 5%, have been marked with a strikeout. Principal Component Analysis (PCA) has been employed using the “factominer” package of R Software. Factorial RBD design has been employed using the “doebioresearch” package of R software.

### Statement regarding plant guidelines

The use of plant parts in the study complies with international, national, and/or institutional guidelines.

## Results

### Analysis of variance (ANOVA) for traits

Table 2 provides the results of the analysis of variance for agro-morphological features observed in early cauliflower CMS lines and their maintainers. There were significant differences among the groups (CMS lines and maintainers) for the observed agro-morphological traits except PH. No significant difference observed between the groups.

**Table 2:** Analysis of variance (ANOVA) for agro-morphological and curding traits observed from CMS lines carrying *Can*, *Ogura* and *Tour* cytoplasm’s and their maintainers in Indian cauliflower.

Source of variation	DF	PH (cm)	PS (cm)	LL (cm)	LW (cm)	NLP (No./Plant)	RL (cm)	RW (cm)	GPW (g)	MCW (g)	NCW (g)	MCY (t/ha)	HI (cm)
Between the group	2	87.6	104.5	19.4	5.1	0.8	6538.0	411.5	871.5	0.3	4.1	0.5	0.001
Among the group	27	46.2	206.3*	123.2*	9.4*	6.2*	67,800.5*	7019.0*	5460.9*	6.7*	8.7*	8.1*	0.006*
Error	54	29.8	32.9	25.2	4.2	2.5	7285.1	987.8	913.5	3.0	3.6	1.3	0.001

*DF* Degree of freedom.

*Significant at 5% level of significance when tested against MSS due to error.

### Variation in agro-morphological and curding traits

**Figure 1:**
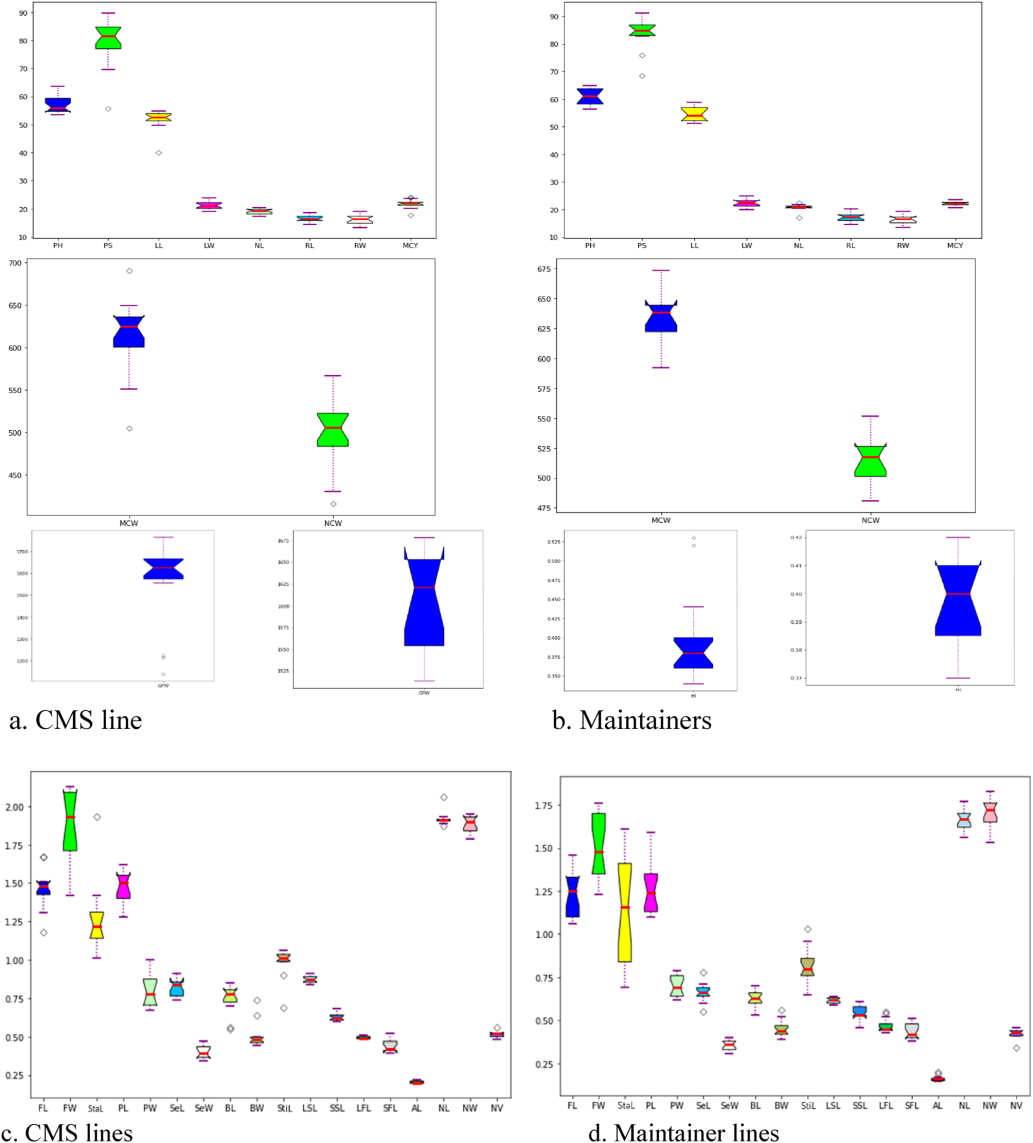
Box plot of 12 agro-morphological traits of *Canarianse* based CMS lines (**a**) and their maintainers (**b**) in early group of Indian cauliflower. Box plot of 18 floral traits of *E. canarianse* based CMS lines (**c**) and their maintainers (**d**) in early group of Indian cauliflower.

The CMS lines and their maintainers showed significant differences for all the observed agro-morphological traits (Table 3). Box plot of these traits from the CMS lines (Fig. 1a) and their maintainers (Fig. 1b) depicts minimum, first quartile (Q1), median, third quartile (Q3) and maximum values of the traits. The box plot analysis showed wide variation in both CMS and maintainer lines for PH, PS, MCW, NCW and GPW. PH in CMS lines was ranged from 53.5 cm in *Can *(DC-94-2) to 63.6 cm in *Ogu* (DC-98-4) and in maintainers from 58.1 cm (DC-41-5) to 64.8 cm (DC-63). PS ranged from 55.7 cm in *Can *(DC-41-5) to 89.8 cm in *Tour* (DC-98-4) in CMS lines. LL, LW and NLP also showed significant variation in CMS lines and maintainers. LW ranged from 40.0 cm in *Can *(DC-41-5) to 54.8 cm in *Can *(DC-63) and LL from 19.1 cm in *Can *(DC-67) to 23.9 cm in *Ogu* (DC-98-4), respectively. NLP were maximum in *Tour* (DC-41-5) (20.3) in CMS lines and DC-63 (22.4) in testers. GPW ranged from 1215.0 g in *Can *(DC-98-4) to 1766.3 g in *Can *(DC-209). Among maintainers, the GPW was highest in DC-41-5 (1677.9 g). *Can *(DC-903) (690.9 g; 567.0 g) in CMS lines and DC-63 (673.8 g; 551.7 g) in testers had maximum MCW and NCW, respectively. MCY was highest in *Can *(DC-903), ranging from 17.7 to 24.2 t/ha. It was the greatest among the testers in DC-63 (23.6 t/ha). HI ranged from 0.34 to 0.53 with the highest in *Can *(DC-41-5) in CMS lines. Among maintainers, it was highest in DC-8 (0.42). RL (18.6 cm) and RW (19.1 cm) were the greatest in CMS *Can *(DC-8) while in maintainers these were maximum in DC-94-2 (20.2 cm) and DC 41-5 (19.4 cm), respectively.

**Table 3:** Agro-morphological and curding traits observed from CMS lines carrying *Can*, *Ogura* and *Tour* cytoplasm’s and their maintainers in Indian cauliflower.

CMS lines /maintainers	PH (cm)	PS (cm)	LL (cm)	LW (cm)	NLP (No./plant)	GPW (g)	MCW (g)	NCW (g)	RL (cm)	RW (cm)	MCY (t/ha)	HI
CMS lines												
*Can* (DC-23)	62.4 (± 1.17)	83.5 (± 2.80)	53.7 (± 2.5)	21.1 (± 1.5)	18.9 (± 0.4)	1685.8 (± 24.0)	609.1 (± 13.0)	483.3 (± 24.6)	16.3 (± 1.5)	16.5 (± 1.7)	21.3 (± 0.5)	0.36 (± 0.01)
*Can* (DC-98-4)	55.9 (± 3.56)	71.1 (3.85)	54.2 (± 3.3)	20.1 (± 5.1)	18.1 (± 1.6)	1215.0 (± 79.2)	504.8 (± 26.7)	416.2 (± 32.1)	17.3 (± 0.3)	16.3 (± 1.5)	17.7 (± 0.9)	0.42 (± 0.01)
*Can* (DC-7)	59.2 (± 4.30)	87.9 (± 4.54)	52.0 (± 2.0)	22.5 (± 1.7)	19.3 (± 2.8)	1555.5 (± 104.4)	550.9 (± 13.9)	430.1 (± 17.8)	18.0 (± 3.5)	17.4 (± 2.4)	20.4 (± 0.5)	0.35 (± 0.03)
*Can* (DC-8)	53.9 (± 2.73)	69.5 (± 1.51)	52.5 (± 8.3)	20.1 (± 1.8)	20.1 (± 1.9)	1221.3 (± 33.8)	636.8 (± 29.9)	521.7 (± 20.2)	18.6 (± 4.8)	19.1 (± 0.8)	22.3 (± 1.0)	0.52 (± 0.04)
*Can* (DC-94-2)	53.5 (± 3.02)	78.9 (± 1.07)	51.3 (± 1.5)	22.0 (± 0.6)	19.1 (± 1.4)	1624.8 (± 23.2)	627.6 (± 16.6)	505.3 (± 22.2)	15.5 (± 0.7)	14.5 (± 1.7)	22.0 (± 0.6)	0.39 (± 0.01)
*Can* (DC-903)	58.2 (± 7.71)	81.6 (± 10.94)	52.6 (± 5.1)	22.3 (± 3.3)	19.6 (± 0.6)	1573.2 (± 88.4)	690.9 (± 46.1)	567.0 (± 50.4)	16.8 (± 0.8)	16.2 (± 3.0)	24.2 (± 1.6)	0.44 (± 0.06)
*Can* (DC-209)	54.7 (± 1.00)	82.1 (± 4.72)	50.1 (± 0.2)	20.6 (± 1.3)	17.3 (± 0.7)	1766.3 (± 27.4)	636.9 (± 13.6)	522.7 (± 2.8)	15.8 (± 0.7)	14.2 (± 2.0)	23.6 (± 0.5)	0.36 (± 0.01)
*Can* (DC-41-5)	55.2 (± 4.10)	55.7 (± 3.58)	40.0 (± 17.1)	22.2 (± 1.3)	19.6 (± 0.2)	1139.3 (± 85.6)	600.2 (± 8.7)	486.1 (± 7.0)	18.3 (± 0.3)	16.0 (± 2.0)	21.0 (± 0.3)	0.53 (± 0.05)
*Can* (DC-18)	61.7 (± 7.86)	84.8 (± 9.86)	53.9 (± 4.6)	20.1 (± 1.0)	18.4 (± 0.8)	1652.4 (± 132.8)	649.5 (± 62.6)	529.8 (± 54.5)	15.1 (± 1.4)	17.3 (± 1.5)	24.0 (± 2.3)	0.39 (± 0.04)
*Can* (DC-63)	58.8 (± 7.07)	79.7 (± 6.49)	54.8 (± 4.6)	21.8 (± 2.8)	19.6 (± 1.5)	1647.5 (± 46.6)	618.1 (± 30.1)	500.6 (± 24.5)	16.9 (± 2.2)	18.0 (± 1.7)	22.9 (± 1.1)	0.37 (± 0.02)
*Can* (DC-67)	54.0 (± 6.66)	79.0 (± 4.18)	50.7 (± 3.2)	19.1 (± 1.3)	19.7 (± 2.2)	1703.3 (± 9.7)	571.9 (± 65.6)	459.0 (± 61.3)	16.0 (± 0.7)	16.6 (± 1.5)	21.2 (± 2.4)	0.34 (± 0.04)
*Ogu*(DC-98-4)	63.6 (± 3.10)	83.1 (± 11.41)	52.4 (± 3.1)	23.9 (± 0.7)	18.1 (± 0.8)	1726.0 (± 189.6)	608.7 (± 1.2)	487.9 (± 2.4)	14.4 (± 0.6)	14.8 (± 2.0)	21.3 (± 0.0)	0.36 (± 0.04)
*Ogu*(DC-41-5)	59.3 (± 7.51)	88.0 (± 6.00)	54.0 (± 4.1)	20.6 (± 1.0)	19.7 (± 0.3)	1607.1 (± 76.8)	633.1 (± 14.9)	522.3 (± 10.7)	16.1 (± 1.4)	16.4 (± 3.1)	22.2 (± 0.5)	0.39 (± 0.02)
*Ogu*(DC-67)	55.2 (± 2.36)	77.0 (± 3.61)	52.5 (± 1.6)	21.5 (± 0.7)	17.7 (± 0.6)	1667.4 (± 65.6)	624.4 (± 10.3)	506.0 (± 15.3)	16.0 (± 1.5)	13.4 (± 0.6)	21.9 (± 0.4)	0.37 (± 0.02)
*Tour* (DC-98-4)	62.2 (± 1.07)	89.8 (± 4.62)	54.1 (± 2.7)	23.2 (± 0.5)	17.8 (± 1.1)	1626.0 (± 100.2)	575.3 (± 56.6)	454.6 (± 55.3)	14.7 (± 0.0)	13.5 (± 1.3)	20.2 (± 2.0)	0.36 (± 0.04)
*Tour* (DC-41-5)	54.3 (± 2.31)	74.8 (± 3.56)	49.7 (± 2.6)	19.4 (± 1.9)	20.3 (± 0.9)	1592.8 (± 56.3)	635.8 (± 24.1)	520.5 (± 25.6)	17.8 (± 0.8)	14.8 (± 3.0)	22.2 (± 0.8)	0.40 (± 0.00)
*Tour* (DC-67)	56.0 (± 1.76)	84.7 (± 3.06)	51.7 (± 0.7)	20.6 (± 1.0)	20.3 (± 1.5)	1640.4 (± 21.3)	626.4 (± 15.8)	532.3 (± 12.8)	16.8 (± 1.0)	17.8 (± 1.1)	21.9 (± 0.6)	0.38 (± 0.01)
Maintainers												
DC-23	61.0 (± 7.84)	85.4 (± 0.84)	51.9 (± 4.2)	21.3 (± 1.2)	17.0 (± 2.5)	1659.2 (± 39.5)	638.3 (± 12.9)	519.3 (± 17.6)	17.7 (± 1.5)	15.2 (± 0.4)	22.3 (± 0.5)	0.38 (± 0.00)
DC-98-4	58.3 (± 2.85)	82.7 (± 0.88)	51.1 (± 2.2)	21.5 (± 2.8)	20.8 (± 0.7)	1537.1 (± 73.0)	627.9 (± 49.3)	502.5 (± 49.9)	15.2 (± 1.7)	15.2 (± 1.1)	22.0 (± 1.7)	0.41 (± 0.05)
DC-7	56.4 (± 4.44)	84.8 (± 8.49)	52.4 (± 4.2)	22.7 (± 1.2)	21.7 (± 0.9)	1646.0 (± 41.7)	638.9 (± 39.4)	525.4 (± 48.2)	16.9 (± 1.6)	17.0 (± 0.6)	22.4 (± 1.4)	0.39 (± 0.03)
DC-8	63.3 (± 2.73)	87.7 (± 3.84)	56.8 (± 2.8)	21.1 (± 3.3)	20.8 (± 2.7)	1559.1 (± 45.7)	649.2 (± 37.4)	527.4 (± 30.7)	16.9 (± 2.6)	18.3 (± 3.0)	22.7 (± 1.3)	0.42 (± 0.03)
DC-94-2	63.8 (± 7.13)	83.3 (± 4.16)	58.1 (± 6.3)	25.0 (± 2.9)	20.4 (± 3.0)	1644.3 (± 50.6)	657.6 (± 19.9)	531.5 (± 29.3)	20.2 (± 2.0)	17.3 (± 1.0)	23.0 (± 0.7)	0.40 (± 0.01)
DC-903	60.8 (± 4.11)	91.2 (± 3.89)	54.4 (± 3.0)	24.6 (± 2.5)	20.5 (± 1.3)	1548.5 (± 56.0)	617.0 (± 3.2)	500.1 (± 17.0)	18.1 (± 0.8)	13.6 (± 1.8)	21.6 (± 0.1)	0.40 (± 0.02)
DC-209	57.7 (± 12.86)	75.9 ± (± 3. ± 89)	51.3 (± 6.3)	20.1 (± 2.5)	21.2 (± 1.4)	1620.4 (± 74.6)	592.4 (± 27.9)	481.1 (± 21.7)	14.8 (± 2.0)	14.1 (± 1.3)	20.7 (± 1.0)	0.37 (± 0.01)
DC-41-5	58.1 (± 4.48)	68.5 (± 2.04)	54.2 (± 3.3)	23.3 (± 0.0)	21.4 (± 2.6)	1677.9 (± 92.2)	639.8 (± 34.9)	517.4 (± 26.1)	19.0 (± 1.0)	16.7 (± 2.4)	22.4 (± 1.2)	0.38 (± 0.03)
DC-18	63.7 (± 11.20)	86.2 (± 13.96)	58.9 (± 6.0)	21.2 (± 1.3)	20.9 (± 0.4)	1620.6 (± 126.2)	627.6 (± 16.0)	506.8 (± 19.7)	17.9 (± 1.0)	19.4 (± 2.5)	21.9 (± 0.6)	0.39 (± 0.03)
DC-63	64.8 (± 6.91)	87.4 (± 10.39)	53.2 (± 2.0)	22.5 (± 2.0)	22.4 (± 1.4)	1663.6 (± 55.8)	673.8 (± 4.2)	551.7 (± 8.5)	14.7 (± 1.5)	15.9 (± 1.0)	23.6 (± 0.1)	0.41 (± 0.01)
DC-67	64.2 (± 6.74)	84.6 (± 3.47)	57.0 (± 4.7)	23.2 (± 1.0)	21.1 (± 1.1)	1513.6 (± 67.2)	615.9 (± 9.7)	495.2 (± 13.9)	17.3 (± 0.7)	17.2 (± 3.2)	21.6 (± 0.3)	0.41 (± 0.01)
C.D. (0.05)	NS	9.4	8.2	3.4	2.6	139.9	51.5	49.5	2.8	3.1	1.8	0.05
C.V. (%)	9.2	7.1	9.7	9.4	8.0	5.4	5.1	6.1	10.3	11.7	5.2	7.80

*CD* critical difference,* CV* coefficient of variation.

### Variation in CMS lines and fertile maintainers for floral traits

**Figure 2:**
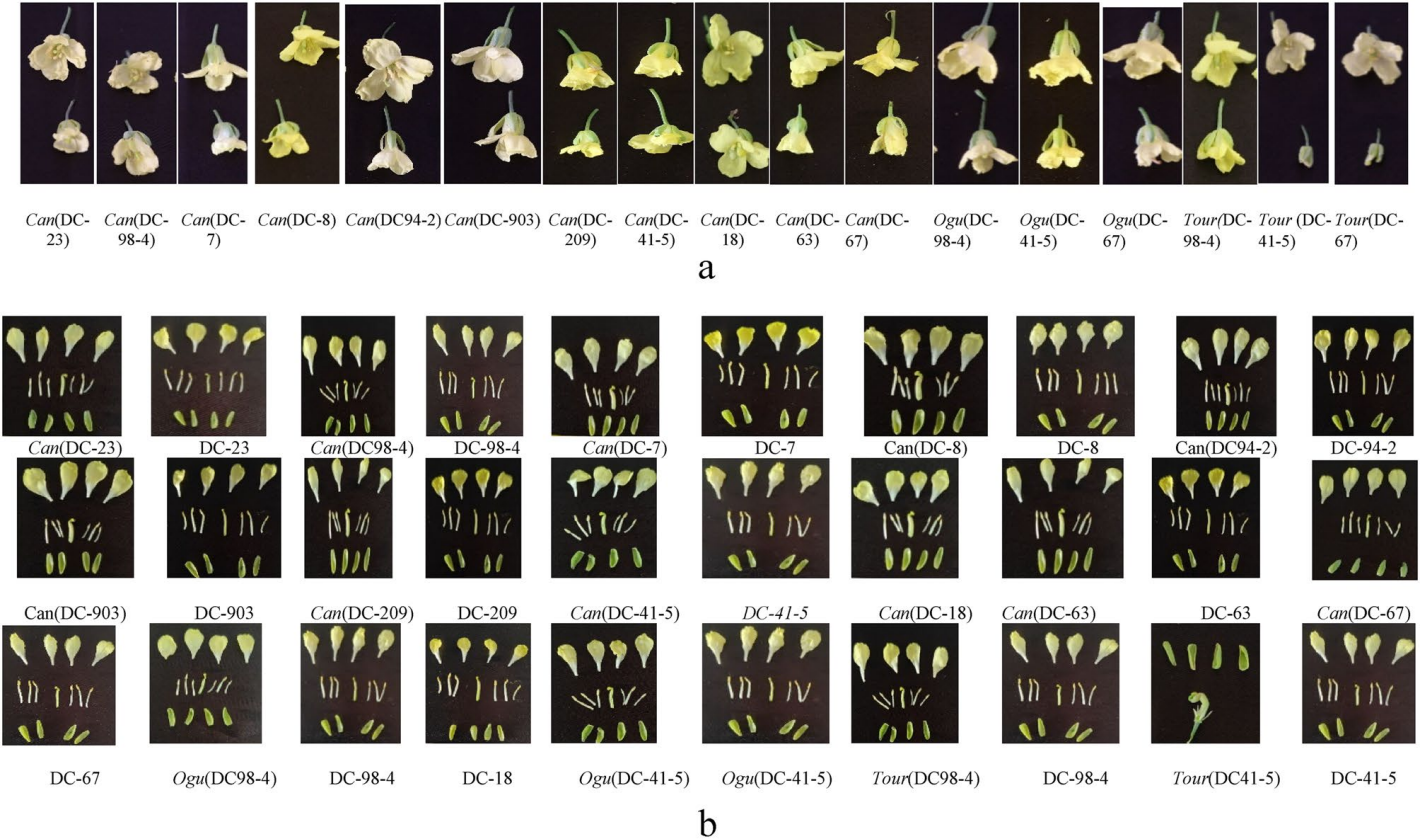
(**a**) Flowers of fertile maintainers (top side) and CMS lines (bottom side) of cauliflower. (**b**) Flower floral parts (sepals, petals, stigma and anthers) of CMS lines and maintainers in early group of Indian cauliflower.

The ANOVA for 18 floral traits in CMS lines and their maintainers is Table 4. Table 5 displays average values of 18 floral traits observed in CMS lines and their maintainers. The box plot of floral traits showed wide range (minimum, maximum), median, first quartile (Q1) and third quartile (Q3) in CMS lines (Fig. 1c) and their maintainers (Fig. 1d). Box plot analysis also revealed large variation in floral traits such as FL, FW, PL, PW, BW, StiL and NL in CMS lines. The cytoplasmic sterility reduced overall flower size and size of reproductive organs (stigma and stamens) in CMS lines (Fig. 2a). The flower parts (sepals, petals, stigma and anthers) of CMS lines and their maintainers of early cauliflower are presented in Fig. 2b. All the observed floral traits showed a significant difference in the CMS lines and maintainers. The tiniest and narrowest flowers were observed in all the three *Tour*-based CMS lines. FD ranged from 1.23 cm in *Tour* (DC-67) to 1.76 cm in *Can *(DC-94-2) in CMS lines and DC-41-5 (2.13 cm) in maintainers. *Can *(DC-41-5) had the shortest StaL (1.61 cm) in CMS lines while DC-7 had the longest StaL (1.93 cm) in maintainers. PL and PW were ranged from 1.12 cm [*Can *(DC-18)] to 1.59 cm [*Can *(DC-7)] and 0.62 cm [*Can *(DC-209)] to 0.79 cm [*Can *(DC-67)], respectively. DC-63 (1.62 cm) and DC-98-4 (1.93 cm) had the highest values of these two petal parameters. SL and SW were ranged from 0.69 to 0.78 cm and 0.31 to 0.40 cm in CMS lines. DC-67 had maximum SL (0.91 cm) while DC-63 had the widest sepals (0.47 cm). BW ranged from 0.39 to 0.56 cm and BL from 0.53 cm to 0.70 cm.

**Table 4:** Analysis of variance (ANOVA) for floral traits observed from CMS lines carrying *Can*, *Ogura* and *Tour* cytoplasm’s and their maintainers in Indian cauliflower.

Source of variation	DF	FL (c m)	FD (cm)	StaL (cm)	PL (cm)	PW (cm)	SL (cm)	SW (cm)	BL (cm)	BW (cm)	StiL (cm)	LSL (mm)	SSL (mm)	LFL (mm)	SFL (mm)	AL (mm)	NL (mm)	NW (mm)	NV (µl/10 fl)
Between the group	2	0.002	0.003	0.001	0.005	0.000	0.002	0.001	0.002	0.001	0.003	0.001	0.001	0.002	0.001	0.001	0.003	0.003	0.005
Among the group	27	0.094*	0.207*	0.250*	0.081*	0.030*	0.044*	0.017*	0.037*	0.040*	0.050*	0.049*	0.008*	0.003*	0.007*	0.002*	0.056*	0.038*	0.007*
Error	54	0.001	0.002	0.004	0.002	0.002	0.001	0.001	0.002	0.001	0.003	0.001	0.001	0.001	0.001	0.001	0.014	0.003	0.001

*DF* Degree of freedom.

*Significant at 5% level of significance when tested against MSS due to error.

**Table 5:** Floral traits observed from CMS lines carrying Can, *Ogura* and *Tour* cytoplasms and their maintainers in Indian cauliflower.

CMS line/maintainer	FL (c m)	FD (cm)	StaL (cm)	PL (cm)	PW (cm)	SL (cm)	SW (cm)	BL (cm)	BW (cm)
*Can* (DC-23)	1.27 (± 0.04)	1.35 (± 0.76)	1.10 (± 0.02)	1.26 (± 0.04)	0.77 (± 0.05)	0.64 (± 0.03)	0.33 (± 0.05)	0.63 (± 0.08)	0.43 (± 0.10)
*Can* (DC-98-4)	1.10 (± 0.02)	1.28 (± 0.72)	1.35 (± 0.03)	1.19 (± 0.15)	0.68 (± 0.00)	0.69 (± 0.01)	0.36 (± 0.01)	0.66 (± 0.00)	0.39 (± 0.01)
*Can* (DC-7)	1.46 (± 0.02)	1.49 (± 0.83)	0.69 (± 0.19)	1.59 (± 0.18)	0.64 (± 0.09)	0.71 (± 0.05)	0.31 (± 0.01)	0.65 (± 0.00)	0.56 (± 0.07)
*Can* (DC-8)	1.40 (± 0.03)	1.63 (± 0.91)	0.72 (± 0.15)	1.46 (± 0.08)	0.62 (± 0.03)	0.55 (± 0.08)	0.33 (± 0.05)	0.53 (± 0.08)	0.47 (± 0.06)
*Can* (DC-94-2)	1.16 (± 0.01)	1.76 (± 1.03)	1.10 (± 0.00)	1.13 (± 0.00)	0.72 (± 0.00)	0.60 (± 0.00)	0.38 (± 0.00)	0.54 (± 0.00)	0.52 (± 0.01)
*Can* (DC-903)	1.42 (± 0.04)	1.38 (± 0.77)	1.16 (± 0.01)	1.24 (± 0.02)	0.76 (± 0.01)	0.68 (± 0.01)	0.35 (± 0.01)	0.59 (± 0.00)	0.41 (± 0.01)
*Can* (DC-209)	1.33 (± 0.03)	1.45 (± 0.81)	0.94 (± 0.01)	1.35 (± 0.00)	0.62 (± 0.02)	0.63 (± 0.01)	0.32 (± 0.04)	0.67 (± 0.03)	0.47 (± 0.05)
*Can* (DC-41-5)	1.17 (± 0.02)	1.74 (± 0.99)	1.61 (± 0.00)	1.35 (± 0.01)	0.76 (± 0.01)	0.69 (± 0.02)	0.39 (± 0.01)	0.60 (± 0.02)	0.44 (± 0.00)
*Can* (DC-18)	1.31 (± 0.01)	1.48 (± 0.85)	0.74 (± 0.10)	1.12 (± 0.02)	0.69 (± 0.04)	0.66 (± 0.02)	0.33 (± 0.01)	0.64 (± 0.00)	0.42 (± 0.00)
*Can* (DC-63)	1.16 (± 0.01)	1.73 (± 0.99)	1.50 (± 0.04)	1.38 (± 0.00)	0.72 (± 0.00)	0.78 (± 0.00)	0.40 (± 0.00)	0.70 (± 0.01)	0.46 (± 0.00)
*Can* (DC-67)	1.06 (± 0.04)	1.74 (± 0.98)	1.49 (± 0.00)	1.36 (± 0.01)	0.79 (± 0.01)	0.69 (± 0.01)	0.38 (± 0.00)	0.59 (± 0.00)	0.42 (± 0.00)
*Ogu*(DC-98-4)	1.32 (± 0.01)	1.70 (± 0.98)	0.84 (± 0.01)	1.16 (± 0.00)	0.76 (± 0.05)	0.64 (± 0.00)	0.38 (± 0.01)	0.64 (± 0.00)	0.41 (± 0.00)
*Ogu*(DC-41-5)	1.34 (± 0.04)	1.43 (± 0.80)	1.31 (± 0.28)	1.10 (± 0.01)	0.62 (± 0.00)	0.67 (± 0.01)	0.31 (± 0.02)	0.61 (± 0.03)	0.50 (± 0.01)
*Ogu*(DC-67)	1.25 (± 0.07)	1.64 (± 0.89)	0.83 (± 0.01)	1.13 (± 0.00)	0.73 (± 0.05)	0.66 (± 0.03)	0.36 (± 0.00)	0.60 (± 0.02)	0.44 (± 0.07)
*Tour* (DC-98-4)	1.07 (± 0.04)	1.2 (± 0.70)	1.38 (± 0.10)	1.13 (± 0.01)	0.64 (± 0.00)	0.66 (± 0.01)	0.36 (± 0.01)	0.66 (± 0.00)	0.39 (± 0.01)
*Tour* (DC-41-5)	1.07 (± 0.01)	1.33 (± 1.06)	1.41 (± 0.00)	1.24 (± 0.07)	0.68 (± 0.01)	0.69 (± 0.01)	0.35 (± 0.01)	0.62 (± 0.00)	0.50 (± 0.00)
*Tour* (DC-67)	1.07 (± 0.01)	1.23 (± 1.06)	1.44 (± 0.00)	1.31 (± 0.07)	0.65 (± 0.01)	0.66 (± 0.01)	0.38 (± 0.08)	0.66 (± 0.00)	0.47 (± 0.07)
Maintainers									
DC-23	1.50 (± 0.00)	1.90 (± 1.10)	1.21 (± 0.02)	1.52 (± 0.00)	0.80 (± 0.00)	0.84 (± 0.01)	0.34 (± 0.02)	0.82 (± 0.29)	0.74 (± 0.01)
DC-98-4	1.51 (± 0.02)	2.11 (± 1.21)	1.07 (± 0.02)	1.50 (± 0.01)	1.00 (± 0.02)	0.85 (± 0.00)	0.43 (± 0.00)	0.80 (± 0.01)	0.46 (± 0.01)
DC-7	1.31 (± 0.01)	1.42 (± 0.81)	1.93 (± 0.01)	1.39 (± 0.01)	0.69 (± 0.00)	0.90 (± 0.01)	0.45 (± 0.03)	0.80 (± 0.01)	0.46 (± 0.01)
DC-8	1.67 (± 0.03)	1.68 (± 0.95)	1.42 (± 0.01)	1.56 (± 0.01)	0.71 (± 0.00)	0.74 (± 0.02)	0.39 (± 0.02)	0.70 (± 0.00)	0.50 (± 0.01)
DC-94-2	1.43 (± 0.01)	2.07 (± 1.18)	1.03 (± 0.01)	1.29 (± 0.02)	0.78 (± 6.31)	0.74 (± 0.00)	0.37 (± 0.01)	0.75 (± 0.00)	0.49 (± 0.01)
DC-903	1.42 (± 0.02)	2.13 (± 1.21)	1.01 (± 0.04)	1.28 (± 0.00)	0.67 (± 0.03)	0.77 (± 0.03)	0.36 (± 0.01)	0.75 (± 0.00)	0.49 (± 0.03)
DC-209	1.44 (± 0.01)	2.05 (± 1.17)	1.22 (± 0.00)	1.49 (± 0.01)	0.80 (± 0.01)	0.84 (± 0.00)	0.43 (± 0.02)	0.55 (± 0.01)	0.44 (± 0.01)
DC-41-5	1.48 (± 0.02)	2.13 (± 1.24)	1.23 (± 0.01)	1.41 (± 0.01)	0.70 (± 0.00)	0.76 (± 0.01)	0.38 (± 0.01)	0.85 (± 0.02)	0.48 (± 0.00)
DC-18	1.50 (± 0.02)	1.93 (± 1.09)	1.22 (± 0.00)	1.54 (± 0.01)	0.77 (± 0.01)	0.86 (± 0.01)	0.35 (± 0.00)	0.81 (± 0.01)	0.47 (± 0.01)
DC-63	1.18 (± 0.02)	1.74 (± 0.99)	1.33 (± 0.01)	1.62 (± 0.02)	0.95 (± 0.04)	0.79 (± 0.01)	0.47 (± 0.00)	0.56 (± 0.01)	0.64 (± 0.00)
DC-67	1.67 (± 0.00)	1.68 (± 0.97)	1.29 (± 0.02)	1.62 (± 0.00)	0.96 (± 5.94)	0.91 (± 0.01)	0.44 (± 0.00)	0.78 (± 0.02)	0.45 (± 0.01)
CD (5%)	0.04	0.08	0.10	0.07	0.07	0.05	0.04	0.08	0.05
(CV %)	5.90	6.76	5.12	6.13	5.51	6.92	7.00	7.53	6.11

CMS line/maintainer	StiL (cm)	LSL (mm)	SSL (mm)	LFL (mm)	SFL (mm)	AL (mm)	NL (mm)	NW (mm)	NV (µl/10 fl)
*Can* (DC-23)	0.83 (± 0.10)	0.63 (± 0.03)	0.46 (± 0.03)	0.55 (± 0.00)	0.38 (± 0.03)	0.15 (± 0.00)	1.62 (± 0.08)	1.73 (± 0.05)	0.34 (± 0.02)
*Can* (DC-98-4)	0.96 (± 0.02)	0.62 (± 0.01)	0.53 (± 0.02)	0.45 (± 0.01)	0.39 (± 0.00)	0.15 (± 0.00)	1.73 (± 0.09)	1.62 (± 0.01)	0.45 (± 0.01)
*Can* (DC-7)	0.65 (± 0.04)	0.59 (± 0.01)	0.53 (± 0.00)	0.45 (± 0.00)	0.42 (± 0.04)	0.15 (± 0.00)	1.60 (± 0.22)	1.72 (± 0.00)	0.45 (± 0.01)
*Can* (DC-8)	0.80 (± 0.07)	0.59 (± 0.04)	0.53 (± 0.02)	0.44 (± 0.00)	0.42 (± 0.00)	0.19 (± 0.08)	1.62 (± 0.11)	1.71 (± 0.06)	0.44 (± 0.01)
*Can* (DC-94-2)	0.78 (± 0.00)	0.62 (± 0.04)	0.57 (± 0.05)	0.54 (± 0.02)	0.51 (± 0.00)	0.16 (± 0.00)	1.56 (± 0.39)	1.75 (± 0.01)	0.41 (± 0.02)
*Can* (DC-903)	1.03 (± 0.01)	0.59 (± 0.06)	0.53 (± 0.03)	0.45 (± 0.01)	0.41 (± 0.01)	0.15 (± 0.01)	1.62 (± 0.22)	1.68 (± 0.03)	0.44 (± 0.00)
*Can* (DC-209)	0.92 (± 0.05)	0.64 (± 0.02)	0.59 (± 0.01)	0.48 (± 0.02)	0.49 (± 0.04)	0.16 (± 0.00)	1.68 (± 0.06)	1.75 (± 0.04)	0.41 (± 0.01)
*Can* (DC-41-5)	0.84 (± 0.02)	0.62 (± 0.00)	0.59 (± 0.02)	0.48 (± 0.02)	0.50 (± 0.04)	0.16 (± 0.00)	1.73 (± 0.03)	1.76 (± 0.11)	0.42 (± 0.01)
*Can* (DC-18)	0.68 (± 0.00)	0.64 (± 0.03)	0.51 (± 0.01)	0.45 (± 0.03)	0.39 (± 0.02)	0.16 (± 0.01)	1.68 (± 0.16)	1.53 (± 0.04)	0.43 (± 0.04)
*Can* (DC-63)	0.80 (± 0.01)	0.63 (± 0.01)	0.59 (± 0.01)	0.46 (± 0.04)	0.48 (± 0.02)	0.20 (± 0.10)	1.67 (± 0.07)	1.77 (± 0.01)	0.41 (± 0.00)
*Can* (DC-67)	0.86 (± 0.01)	0.59 (± 0.08)	0.54 (± 0.02)	0.45 (± 0.02)	0.43 (± 0.02)	0.16 (± 0.00)	1.63 (± 0.23)	1.70 (± 0.05)	0.45 (± 0.01)
*Ogu*(DC-98-4)	0.74 (± 0.00)	0.63 (± 0.01)	0.53 (± 0.01)	0.46 (± 0.00)	0.41 (± 0.01)	0.17 (± 0.00)	1.74 (± 0.08)	1.63 (± 0.02)	0.43 (± 0.03)
*Ogu*(DC-41-5)	0.85 (± 0.00)	0.64 (± 0.01)	0.61 (± 0.03)	0.45 (± 0.04)	0.39 (± 0.01)	0.15 (± 0.00)	1.70 (± 0.15)	1.83 (± 0.06)	0.46 (± 0.01)
*Ogu*(DC-67)	0.77 (± 0.07)	0.64 (± 0.00)	0.53 (± 0.01)	0.44 (± 0.00)	0.43 (± 0.02)	0.17 (± 0.01)	1.77 (± 0.08)	1.60 (± 0.09)	0.43 (± 0.03)
*Tour* (DC-98-4)	0.96 (± 0.02)	0.62 (± 0.01)	0.53 (± 0.01)	0.43 (± 0.01)	0.40 (± 0.00)	0.16 (± 0.01)	1.66 (± 0.05)	1.65 (± 0.06)	0.41 (± 0.06)
*Tour* (DC-41-5)	0.74 (± 0.01)	0.60 (± 0.02)	0.58 (± 0.01)	0.52 (± 0.05)	0.49 (± 0.02)	0.16 (± 0.00)	1.68 (± 0.04)	1.76 (± 0.07)	0.41 (± 0.04)
*Tour* (DC-67)	0.76 (± 0.01)	0.63 (± 0.05)	0.51 (± 0.08)	0.45 (± 0.02)	0.42 (± 0.05)	0.16 (± 0.00)	1.64 (± 0.04)	1.79 (± 0.07)	0.44 (± 0.04)
Maintainers									
DC-23	0.69 (± 0.27)	0.89 (± 0.00)	0.60 (± 0.01)	0.49 (± 0.02)	0.43 (± 0.04)	0.20 (± 0.01)	1.91 (± 0.00)	1.93 (± 0.01)	0.50 (± 0.03)
DC-98-4	1.05 (± 0.02)	0.90 (± 0.01)	0.61 (± 0.02)	0.50 (± 0.03)	0.42 (± 0.02)	0.20 (± 0.02)	1.91 (± 0.01)	1.93 (± 0.01)	0.52 (± 0.03)
DC-7	0.99 (± 0.01)	0.89 (± 0.00)	0.60 (± 0.03)	0.48 (± 0.00)	0.42 (± 0.02)	0.20 (± 0.01)	1.91 (± 0.02)	1.92 (± 0.04)	0.51 (± 0.02)
DC-8	1.01 (± 0.00)	0.89 (± 0.02)	0.61 (± 0.02)	0.49 (± 0.00)	0.42 (± 0.02)	0.20 (± 0.01)	1.91 (± 0.04)	1.95 (± 0.04)	0.52 (± 0.04)
DC-94-2	1.01 (± 0.02)	0.85 (± 0.03)	0.68 (± 0.01)	0.51 (± 0.04)	0.52 (± 0.01)	0.20 (± 0.01)	1.87 (± 0.06)	1.86 (± 0.04)	0.56 (± 0.01)
DC-903	1.05 (± 0.02)	0.86 (± 0.01)	0.63 (± 0.01)	0.49 (± 0.03)	0.39 (± 0.00)	0.21 (± 0.01)	1.91 (± 0.01)	1.79 (± 0.03)	0.48 (± 0.01)
DC-209	1.02 (± 0.00)	0.84 (± 0.01)	0.65 (± 0.01)	0.50 (± 0.02)	0.51 (± 0.03)	0.20 (± 0.00)	1.89 (± 0.03)	1.87 (± 0.01)	0.52 (± 0.02)
DC-41-5	1.02 (± 0.02)	0.85 (± 0.03)	0.65 (± 0.01)	0.50 (± 0.03)	0.51 (± 0.00)	0.20 (± 0.01)	1.90 (± 0.04)	1.90 (± 0.02)	0.52 (± 0.02)
DC-18	1.06 (± 0.01)	0.87 (± 0.04)	0.63 (± 0.03)	0.50 (± 0.00)	0.40 (± 0.00)	0.22 (± 0.01)	1.92 (± 0.05)	1.82 (± 0.03)	0.50 (± 0.03)
DC-63	0.90 (± 0.00)	0.91 (± 0.02)	0.62 (± 0.01)	0.49 (± 0.01)	0.43 (± 0.01)	0.21 (± 0.00)	2.06 (± 13.04)	1.94 (± 0.01)	0.52 (± 0.02)
DC-67	0.99 (± 0.02)	0.87 (± 0.01)	0.62 (± 0.02)	0.49 (± 0.01)	0.40 (± 0.00)	0.21 (± 0.01)	1.93 (± 0.02)	1.79 (± 0.02)	0.48 (± 0.04)
CD (5%)	0.10	0.04	0.04	0.04	0.04	0.03	0.19	0.09	0.04
(CV %)	6.54	5.73	4.45	5.03	4.95	11.42	6.63	6.03	5.76

*CD* critical difference,* CV* coefficient of variation.

StiL in CMS lines was ranged from 0.65 to 1.03 cm which was significantly less than the maintainers (0.69–1.05 cm). Observations on length of stamens (*i.e.* LSL and SSL) and anther filament (LFL and SFL) showed significant differences in CMS lines and their maintainers. NL was maximum in *Ogu*(DC-67) (1.77 mm) and NW in *Ogu* (DC-41-5) (1.83 mm) while in maintainers, the observed maximum values were 2.06 mm (DC-63) and 1.95 mm (DC-8), respectively. NV also varied significantly in CMS lines (0.34–0.46 µL/10 flowers) and maintainers (0.48–0.56 µL/10 flowers).

### *Can* cytoplasm-associated changes in agro-morphological and floral traits

**Figure 3:**
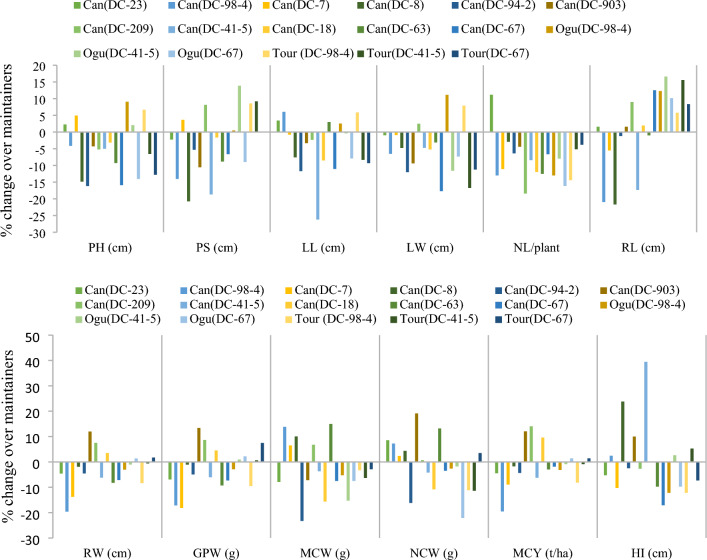
Per cent change in agro-morphological traits due to ingression of *Can* sterile cytoplasm in genotypes of early group of Indian cauliflower.

In comparison to their maintainers, the introgression of nuclear genome of Indian cauliflower genotypes in sterile cytoplasms had significant impact on all 12 of the observed morphological traits (Fig. 3). PL changed in range of −16.1% in *Can *(DC-94-2) to 9.1% in *Can *(DC-98-4) and PS reduced the greatest in *Can *(DC-8) (−20.8%). LL and LW also influenced by sterile cytoplasms in the ranges of -26.2% to 6.1% and -17.7% to 11.2%, respectively. Except for *Can *(DC-23), all *Can* based CMS lines showed reduction in NLP. Impact of *Can* cytoplasm was noticed on GPW (-21.7 to 16.6%), MCW (-19.6 to 12.0%) and NCW (up to 13.4%). The changes in RL and RW ranged from -23.3% to 15.0% and -22.1% to 19.1%, respectively. The sterile cytoplasms also affected MCY, which was decreased in eight CMS lines while slightly increased in 3 CMS lines by 9.6 to 14.0%. *Can* (DC-98-4) experienced the greatest decline (19.5%). HI was also influenced in both the directions.

**Figure 4:**
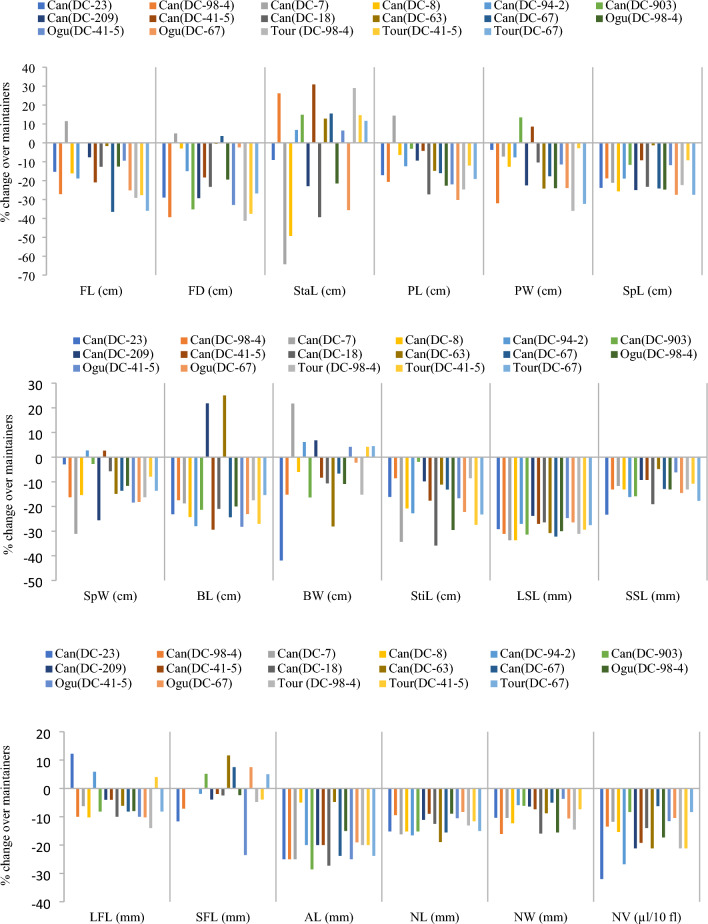
Per cent change in floral traits due to ingression of Can sterile cytoplasm in genotypes of early group of Indian cauliflower.

Contrary to agro-morphological traits, the sterile cytoplasms had highly significant and negative impact on all the 18 observed floral traits (Fig. 4). Eight floral parameters namely SL and SW, StaL, LSL, SSL, AL, NL, NW and NV showed negative impact only. The reduction in FL ranged from -36.5% in *Can* (DC-67) to 11.4% in *Can* (DC-7). Similar trend was observed for FD and StaL. Notable impact was observed on petals (PL: −30.2 to 14.3%; PW: −36.0 to 13.4%), sepals (SL: −1.2 to −27.4%; SW: −31.1 to 2.7%) and floral buds (BL: −29.4 to 25%; BW: −41.5 to 21.7%). Significant reduction was observed in StiL (−1.9 to −35.8%), LSL (−24.7 to -33.70%) and SSL (−4.8 to −23.3%). The CMS introgression reduced NL, NW and NV in range of −18.9 to −8.2%, −16.0 to −8.2% and −32 to −6.2%, respectively.

### Impact of sterile cytoplasms on agro-morphological traits

**Figure 5:**
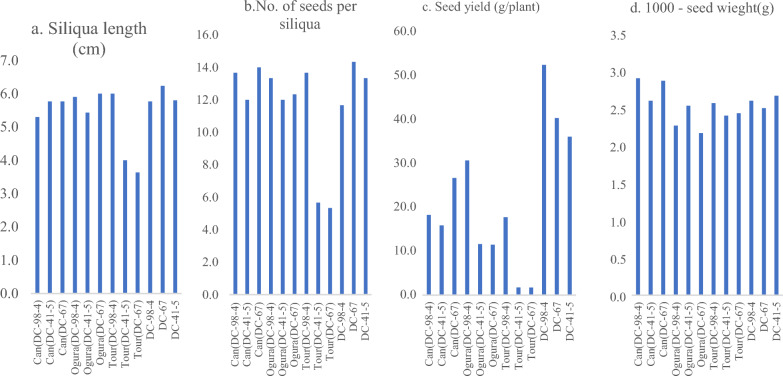
(**a**–**d**) Seed parameters from selected CMS lines and their maintainers in genotypes of early group of Indian cauliflower.

Nine CMS lines developed through introgression of *Tour*, *Ogura* and *Can* cytoplasms in nuclear backgrounds of DC-98-4, DC-67 and DC-41-5 were observed for SqL, NS/siliqua, SY (g/plant) and TW (g). The sterile cytoplasms influenced seed traits and the greatest reduction was observed in *Tour*-based CMS lines (Fig. 5a–d). While, the TW was found to be in the same range, SqL and NS/siliqua showed significant different. SY was significantly affected due to CMS introgression particularly due to *Tour* sterile cytoplasm.

### Principal component, bi-plot and correlation analyses

**Figure 6:**
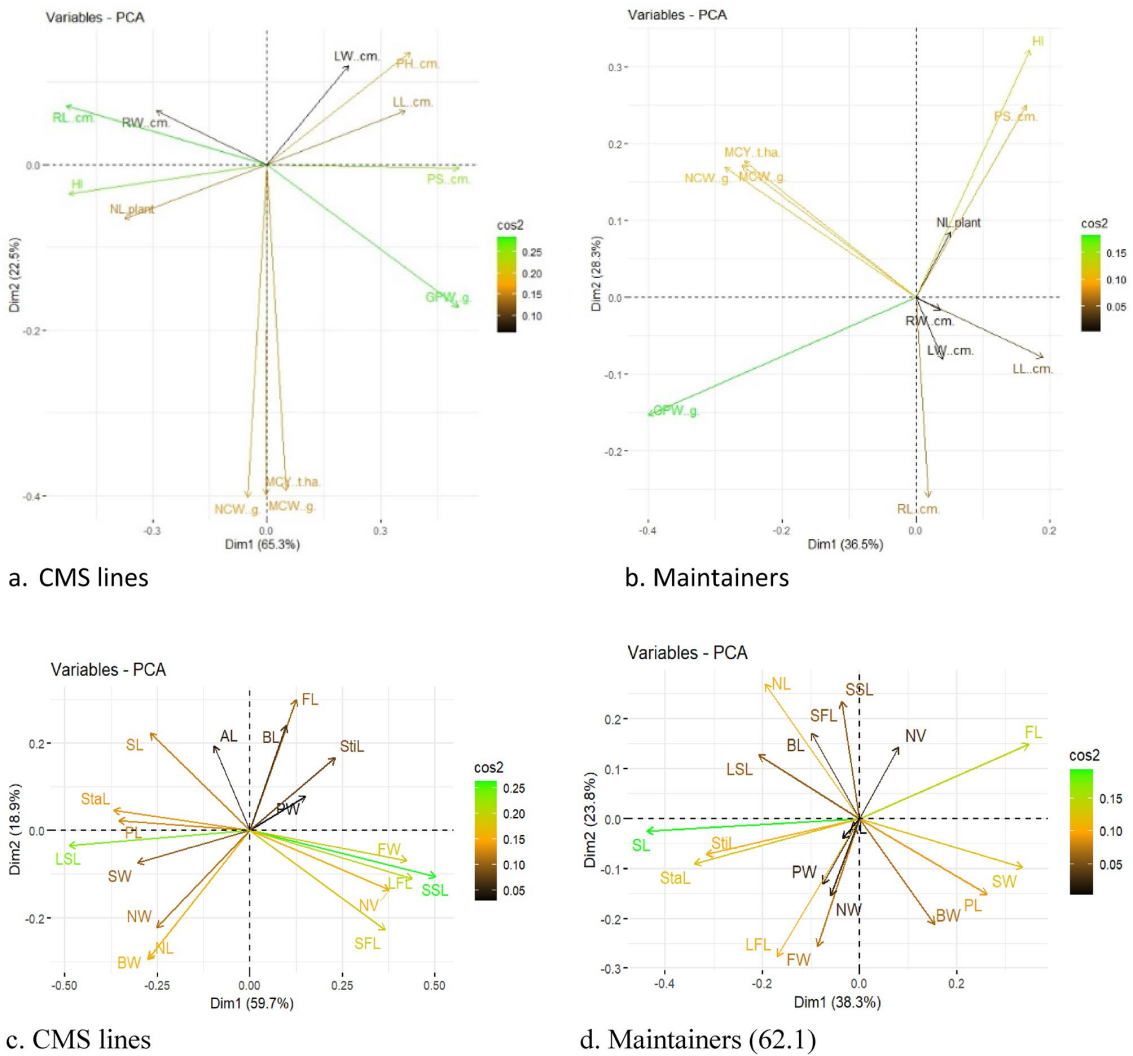
Principal component analysis (PCA) of agro-morphological traits (**a**, **b**) and floral traits (**c**, **d**) in CMS lines and maintainers in early group of Indian cauliflower using "FactoMiner" R Package.

Principal component analysis (PCA) for agro-morphological traits (Fig. 6a, b) and floral traits (Fig. 6c, d) explains prominence of PCA1 (65.3%) and PCA2 (22.5%) combining of 87.8% in explaining variances in CMS lines and PCA1 (36.5%), PCA2 (28.3%) and PCA3 (17.3%) in maintainers (Fig. S1a, b). For floral traits, PCA1 (38.3%) and PCA2 (23.8%) together explained maximum part of variance in CMS lines while only PCA1 could explain 59.7% variance in maintainers (Fig. S1c, d).

Contribution of variables of agro-morphological traits in variation in CMS lines and maintainers is presented in (Fig. S2a, b). and their floral traits in (Fig. S2c, d). RL, GPW, HI and PS are important determinant show similarity in CMS lines while GPW, HI, NCW, MCY and MCW in maintainers .SL, FL, StaL, SW, NL, LFL, StiL, PL, FW and BL in CMS lines and SSL, LSL, LFL, SFL, FW, BW, NV, NL and StaL were contributors in variance in maintainers.

**Figure 7:**
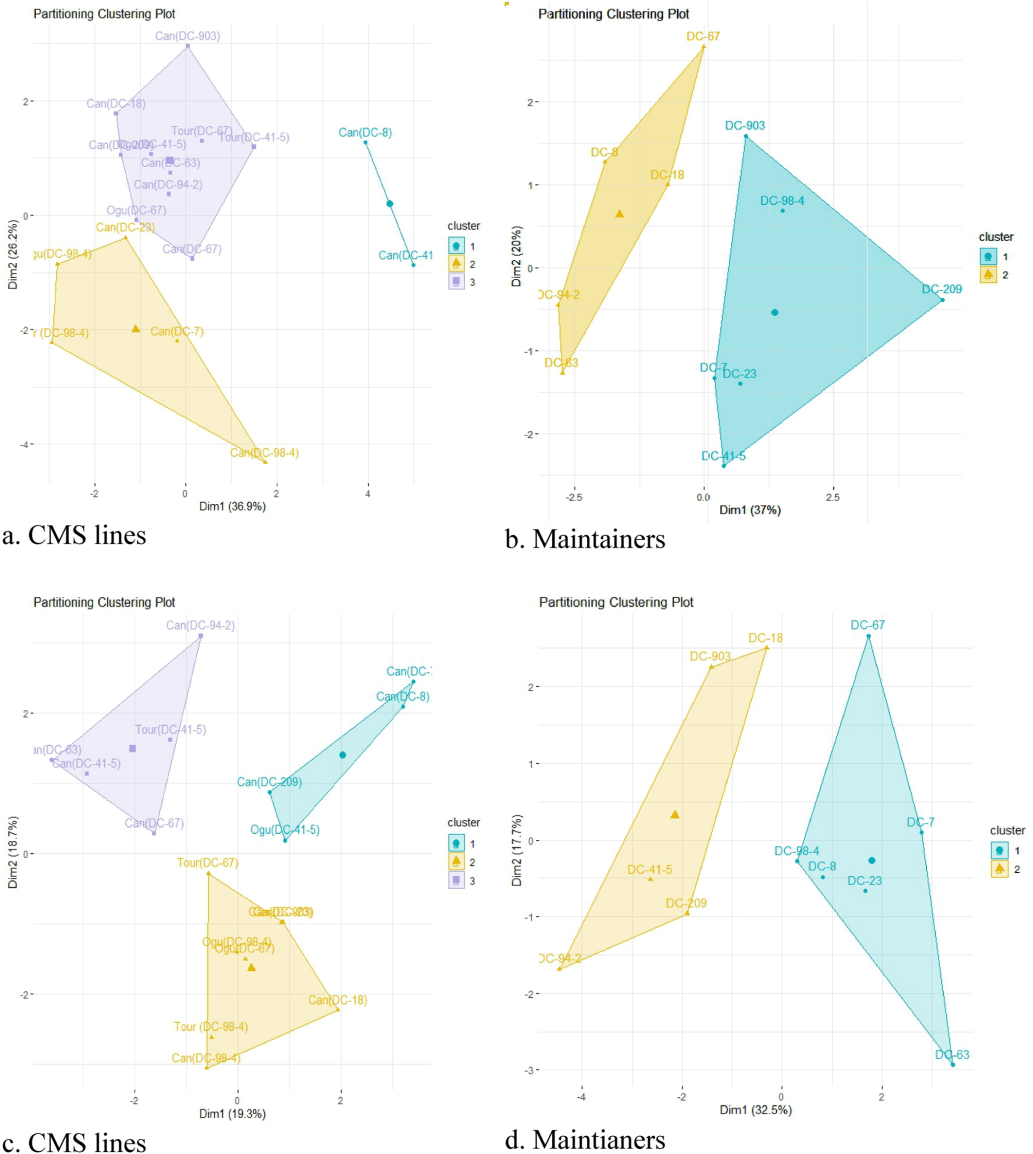
(**a**–**d**) Cluster analysis of CMS lines and their maintainers for agro-morphological trait (**a**, **b**) and floral traits (**c**, **d**) in early group of Indian cauliflower.

Bi-plot analysis for agro-morphological traits (Fig. 6a, b) and floral traits (Fig. 6c, d) in CMS lines and maintainers in early group of Indian cauliflower using "FactoMiner" R Package. This plot interpreted the similarity in most of the agro-morphological traits. HI, RL and GPW showed similarity in CMS lines whereas all the traits showed similarity in maintainers except GPW. For floral traits, LSL and SSL showed similarity in CMS lines whereas all the traits showed similarity in maintainers except SL. Cluster analysis of CMS lines and their maintainers for agro-morphological trait (Fig. 7a, b) and floral traits (Fig. 7c, d) could reveal three and two clusters, respectively.

**Figure 8:**
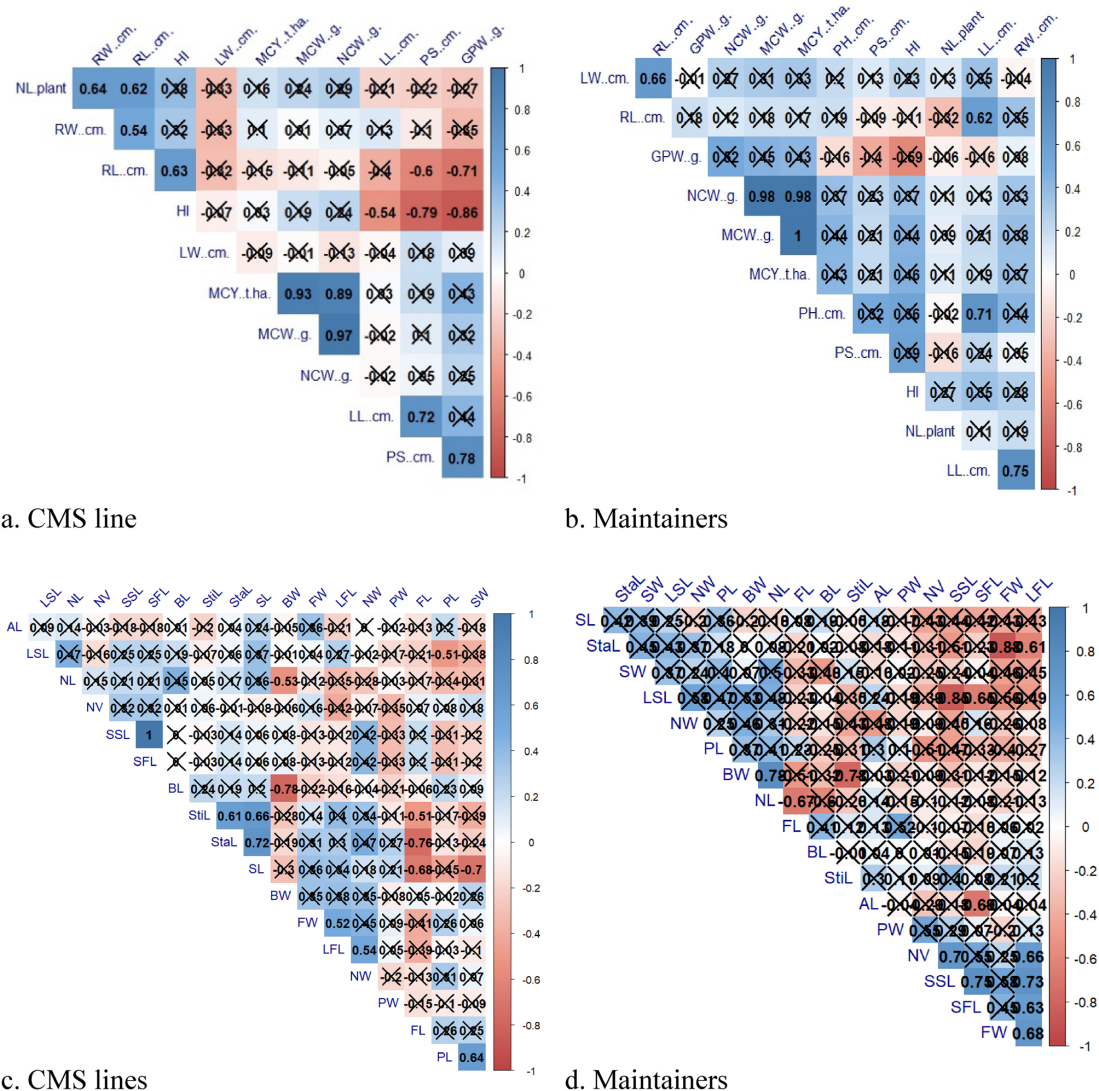
(**a**–**d**) Correlation analysis was done for agro-morphological (**a**, **b**) as well as floral traits (**c**, **d**) in genotypes of early group of Indian cauliflower using the “Hmisc” R package.

Correlation analysis between agro-morphological traits in CMS lines and maintainers is presented in Fig. 8a, b and their floral traits in Fig. 8c, d. A significant positive correlation was observed among the traits such as NCW with MCW and MCY. RL positively correlated with HI and NL positively correlated with RW while GPW negatively correlated with PS and PS negatively correlated with LL in CMS lines. In maintainers MCW positively correlated with MCY and NCW positively correlated with MCW while PS negatively correlated with HI. In CMS lines, RL was strongly correlated with NLP, RW and HI. In floral traits, LFL has strong correlation with FW and NW. StiL significantly correlated with StaL and SL in CMS lines. In maintainers, LFL was strongly correlated with NV, SSL, SFL and FW (Fig. 8c, d). SSL significantly correlated with NV and SFL and BW with NL. These correlation studies are significant at 5% level of significance.

### Variation in CMS lines for F_1_ hybrid seed traits

Table 6 lists the performance of 17 CMS lines of Indian cauliflower for F_1_ hybrid seeds production in two isolations using DC-67 and DC-98-4 fertile inbred lines as male parents. The SqL was ranged from 4.7 cm [*Tou*r (DC-41-5)] to 5.9 cm [*Tour* (DC-67)]. *Can* (DC-23) had maximum NS (14.5/siliqua) while *Tour*-based CMS lines namely *Tour* (DC-41-5) and *Tour* (DC-67) had minimum NS (4.8/siliqua). Among CMS lines, SY was maximum in *Ogu* (DC-98-4) (28.0 g/plants) followed by *Can* (98-4) (24.2 g/plant) and minimum in *Tour* (DC-67) (1.5 g/plant). TW was ranged from 1.9 g [*Can* (DC-18)] to 3.6 g [*Tour* (DC-41-5)]. Satisfactory flowering and seed settings were observed in all the *Can* and *Ogura* cytoplasm-based CMS lines.

**Table 6:** Observations on F_1_ hybrid seed produced from *Can*, *Ogura* and *Tour* cytoplasm carrying CMS lines in Indian cauliflower.

CMS line/male (pollen) parent	Siliqua1 length(cm)			No of seeds per siliqua		
	DC-67	DC-98-4	Mean	DC-67	DC-98-4	Mean
*Can* (DC-23)	5.7 (± 0.40)	6.1 (± 0.42)	5.9 (± 0.28)	14.3 (± 1.53)	14.7 (± 2.08)	14.5 (± 0.28)
*Can* (DC-98-4)	5.5 (± 0.82)	5.3 (± 1.05)	5.4 (± 0.14)	14.0 (± 1.00)	13.7 (± 2.08)	13.8 (± 0.21)
*Can* (DC-7)	5.7 (± 0.60)	5.9 (± 0.61)	5.8 (± 0.14)	14.0 (± 1.00)	14.3 (± 0.58)	14.2 (± 0.21)
*Can* (DC-8)	5.5 (± 0.53)	5.6 (± 0.53)	5.6 (± 0.07)	13.7 (± 1.53)	14.0 (± 1.00)	13.8 (± 0.21)
*Can* (DC-94-2)	5.9 (± 0.71)	4.7 (± 0.21)	5.3 (± 0.85)	14.3 (± 2.08)	14.0 (± 1.00)	14.2 (± 0.21)
*Can* (DC-903)	5.4 (± 0.20)	5.5 (± 0.15)	5.4 (± 0.07)	12.3 (± 2.52)	13.7 (± 1.53)	13.0 (± 0.98)
*Can* (DC-903)	5.6 (± 0.55)	5.5 (± 0.56)	5.5 (± 0.07)	12.3 (± 2.52)	13.3 (± 1.53)	12.8 (± 0.70)
*Can* (DC-41-5)	5.9 (± 0.42)	6.3 (± 0.26)	6.1 (± 0.28)	12.0 (± 2.00)	13.0 (± 1.00)	12.5 (± 0.70)
*Can* (DC-18)	5.4 (± 0.20)	5.4 (± 0.26)	5.4 (± 0.00)	14.0 (± 1.00)	14.0 (± 1.00)	14.0 (± 0.00)
*Can* (DC-63)	5.7 (± 0.40)	5.4 (± 0.15)	5.6 (± 0.21)	13.7 (± 1.53)	13.3 (± 2.08)	13.5 (± 0.28)
*Can* (DC-67)	5.8 (± 0.40)	5.8 (± 0.42)	5.8 (± 0.00)	14.0 (± 1.00)	13.3 (± 1.53)	13.7 (± 0.49)
*Ogura* (DC-98-4)	5.8 (± 0.40)	5.9 (± 0.36)	5.8 (± 0.07)	14.3 (± 1.53)	13.3 (± 1.53)	13.8 (± 0.70)
*Ogura* (DC-41-5)	5.7 (± 0.50)	5.7 (± 0.40)	5.7 (± 0.00)	13.3 (± 1.53)	10.7 (± 7.51)	12.0 (± 1.83)
*Ogura* (DC-67)	5.9 (± 0.25)	6.0 (± 0.26)	5.9 (± 0.07)	12.3 (± 2.08)	13.7 (± 1.53)	13.0 (± 0.98)
*Tour* (DC-98-4)	5.9 (± 0.40)	6.0 (± 0.26)	5.9 (± 0.07)	12.3 (± 0.58)	13.7 (± 1.53)	13.0 (± 0.98)
*Tour* (DC-41-5)	3.8 (± 0.55)	5.7 (± 0.40)	4.7 (± 1.34)	5.3 (± 2.52)	4.3 (± 1.53)	4.8 (± 0.70)
*Tour* (DC-67)	3.6 (± 0.42)	5.8 (± 0.25)	4.7 (± 1.56)	5.3 (± 1.53)	4.3 (± 1.15)	4.8 (± 0.70)
CD at 5% (Male parent)	0.18			NS		
CD at 5% (CMS lines)	0.53			2.31		
CD at 5% (CMS line × male parent)	0.75			NS		

CMS line/male (pollen) parent	Seed yield per plant(g/plant)			1000- seed weight (g)		
	DC-67	DC-98-4	Mean	DC-67	DC-98-4	Mean
*Can* (DC-23)	26.6 (± 0.18)	6.2 (± 0.77)	16.4 (± 14.4)	2.7 (± 1.11)	2.2 (± 0.77)	2.5 (± 0.35)
*Can* (DC-98-4)	34.7 (± 0.10)	13.7 (± 1.53)	24.2 (± 14.8)	3.5 (± 1.72)	2.9 (± 1.53)	3.2 (± 0.42)
*Can* (DC-7)	15.7 (± 0.11)	7.0 (± 1.00)	11.3 (± 6.15)	3.4 (± 1.53)	1.7 (± 1.00)	2.5 (± 1.20)
*Can* (DC-8)	26.7 (± 0.05)	8.5 (± 0.12)	17.6 (± 12.8)	2.5 (± 1.41)	2.7 (± 0.12)	2.6 (± 0.14)
*Can* (DC-94-2)	17.1 (± 0.12)	7.0 (± 1.00)	12.0 (± 7.14)	2.4 (± 2.34)	2.5 (± 1.00)	2.5 (± 0.07)
*Can* (DC-903)	18.1 (± 0.12)	15.0 (± 1.00)	16.6 (± 2.19)	2.5 (± 1.41)	2.6 (± 1.00)	2.5 (± 0.07)
*Can* (DC-903)	16.0 (± 0.09)	15.5 (± 0.95)	15.8 (± 0.35)	2.5 (± 2.00)	2.7 (± 0.95)	2.6 (± 0.14)
*Can* (DC-41-5)	17.0 (± 0.11)	26.0 (± 1.00)	21.5 (± 6.36)	2.4 (± 2.00)	2.4 (± 1.00)	2.4 (± 0.00)
*Can* (DC-18)	7.8 (± 0.02)	17.5 (± 1.13)	12.7 (± 6.85)	1.8 (± 1.15)	1.9 (± 1.13)	1.9 (± 0.07)
*Can* (DC-63)	8.3 (± 0.01)	16.7 (± 2.08)	12.5 (± 5.93)	1.9 (± 0.53)	2.2 (± 2.08)	2.1 (± 0.21)
*Can* (DC-67)	26.7 (± 0.02)	7.7 (± 0.60)	17.2 (± 13.4)	2.9 (± 1.79)	2.1 (± 0.60)	2.5 (± 0.57)
*Ogura* (DC-98-4)	25.4 (± 0.06)	30.7 (± 4.93)	28.0 (± 3.74)	2.5 (± 0.90)	2.3 (± 4.93)	2.4 (± 0.14)
*Ogura* (DC-41-5)	7.2 (± 0.06)	16.7 (± 1.53)	11.9 (± 6.71)	2.8 (± 0.46)	2.6 (± 1.53)	2.7 (± 0.14)
*Ogura* (DC-67)	11.5 (± 0.01)	8.0 (± 1.00)	9.7 (± 2.47)	2.2 (± 0.90)	2.1 (± 1.00)	2.1 (± 0.07)
*Tour* (DC-98-4)	29.4 (± 0.05)	17.7 (± 0.90)	23.6 (± 8.27)	2.9 (± 1.90)	2.6 (± 0.90)	2.7 (± 0.21)
*Tour* (DC-41-5)	3.8 (± 0.02)	3.4 (± 0.10)	3.6 (± 0.28)	3.8 (± 0.70)	3.4 (± 0.10)	3.6 (± 0.28)
*Tour* (DC-67)	1.7 (± 0.05)	1.3 (± 0.55)	1.5 (± 0.28)	2.4 (± 0.35)	2.5 (± 0.55)	2.5 (± 0.07)
CD at 5% (Male parent)	0.58			0.04		
CD at 5% (CMS lines)	1.70			0.12		
CD at 5% (CMS line × male parent)	2.40			0.17		

*CD* critical difference,* CV* coefficient of variation.

### Mitochondrial specific DNA markers for CMS lines

The observations for amplification and level of polymorphism in CMS lines with 25 mtDNA markers are presented in Fig. S3. Two markers, namely P14, P15 and P16 were polymorphic in CMS and fertile maintainers. The marker P14 amplified fragments of 200 bp in CMS lines (lane 1–6, 8,9) and that of 250 bp in fertile maintainers (lane 15–20), whereas P15 amplified distinct bands of 200 bp in CMS lines (lane 3–8) and 250 bp in fertile maintainers (lane 13–18), respectively. P14 showed polymorphism between CMS lines carrying *Ogura* (lane 21, 22, 24) and *canarianse* (lane 1–6, 8, 9) CMS systems and their fertile counterparts. It also generated a unique band (175 bp) for *Tour* cytoplasm (lane 23), separating it from *Ogura* and *canarianse* cytoplasms. P15 could distinguish *canarianse* (lane 3–8) from *Ogura* (lane 21, 22, 24)) and *Tour* (lane 23) cytoplasms, however, it could not differentiate between the latter two systems. Notably, intra-CMS system variation was also observed among the *Can* cytoplasm carrying CMS lines such as *Can* (DC-209) (lane 7) and *Can* (DC-63) (lane 10) which amplified an amplicon of 250 bp while rest of the eight *Can* based CMS lines generated 200 bp with P14 primers. Similar observations were made from P15 which could generate three different bands in *Can* based CMS lines. P16 revealed a polymorphic band (450 bp) for *Ogura* (DC-45) (lane 24) while its maintainer DC-41-5 (lane 18) amplified 500 bp amplicon.

### Sequence analysis

The results of alignment of amplicons produced by the eight genotypes employed in the study using ITS 5a-ITS 4 primer are shown in Fig. S3a. The aligned sequences were filtered from both the ends to remove the gaps and the central 614 residues were finally analyzed. In the aligned sequences, the sequences bearing similarity are shaded with the same colour e.g. A is shaded green, T red, C blue and G black. The unshaded residues are indicating that the residue in question is variable in the compared genotypes. There was not much variation in the analysed sequence up to 460 bases. In this portion, the sequences were observed to be same in all the genotypes except DC-23 and DC-98-4. It was observed that there was C in place of A at 95th position, G in place of T at 163rd position, C in place of T at 402nd, C in place of T at 416th position in DC-23 and DC-98-4. At the same time genotype *Can *(DC-41-5) showed presence of G at this position (416th). At position 432nd there was C in *Can *(DC-41-5) while G in rest of the genotypes. Similar analysis of the sequences revealed that only DC-23, DC-98-4 and *Can* (DC-41-5) showed different pattern as compared to the rest of the genotypes. The results of alignment of amplicons obtained using atpF-atpH primer from the eight genotypes (5 CMS lines and 3 fertile maintainers), two maintainers were common for four CMS lines (Fig. S3b). The central 429 residues were finally analysed. In the aligned sequences, the sequences bearing similarity are shaded with the same colour as in case of ITS 5a-ITS4. The lines showed variation at different positions for nucleotides. *Can* (DC-23) had deletions of A at two positions i.e. at 166th position A is deleted and T is deleted at 299th position. Five conversions were also observed at 178th, 258th, 318th, 370th and 429th positions. The sequence analysis revealed variation among the sterile cytoplasms and also in fertile inbreds for the observed sequences. The deletion was also observed in *Can* (DC-98-4) at 299th position but not in *Can* (DC-41-5) indicating change in sequences. At 323th position, the sequence alignment of atpF-atpH showed difference in the CMS lines namely *Can* (DC-41-5), *Ogu *(DC-41-5) and *Tour *(DC-41-5) and their maintainers DC-41-5. These CMS lines had T, while maintainer DC-41-5 had A. The sequence alignment of amplicons obtained using P16 primer from the eight genotypes (Fig. S3c) revealed no variation between *Can *(DC-23) and DC-23, while *Can *(DC-98-4) and *Can* (DC-41-5) showed variation due to addition and deletion of nucleotides at different positions. In case of rbeL primer (Fig. S3d). The central 663 residues were finally analyzed. In the aligned sequences no variation was detected between *Can *(DC-23) and DC-23 while *Can *(DC-98-4) and *Can *(DC-41-5) showed variation due to the addition and deletion of nucleotides at different positions. The sequence alignment of amplicons obtained using trnL primer from eight genotypes (Fig. S3e) could show addition and deletion of nucleotides in *Can *(DC41-5). It has additions at 14th, 15th, 60th, 74th, and 80th positions. *Can *(DC*-*41-5) had one deletion of nucleotides at 22nd, 32nd, 42nd, 48th, 50th and 57th positions.

## Discussion

In terms of agro-morphological features, the CMS lines carrying *Can* and *Tour* sterile cytoplasms are on par with the CMS lines carrying *Ogura* sterile cytoplasm. However, there was a significant difference in floral traits between the cauliflower CMS lines carrying these three sterile cytoplasms. *Tour* cytoplasm-based CMS lines namely *Tour *(DC-41-5) and *Tour *(DC-67) showed a considerable reduction in floral traits.

### Variations in agro-morphological, floral and seed traits

Cauliflower is a crop that was introduced to the Indian subcontinent, and the original genotypes had a temperate flowering habit meaning that they needed a protracted period of cold temperature for proper bolting and flowering for seed production. Newly evolved cauliflower had varied morphologies in terms of stalk length, leaf orientation, leaf size and colour, curd size and colour and plant frame^19^. ‘Indian cauliflower’ or ‘tropical cauliflower’ was the name given to the evolved ecotype particularly to the early and mid-early groups. The cytoplasmic male sterility is widely used genetic mechanism for hybrid breeding in Brassica crops. It arises as a result of a genomic conflict between the mitochondrial and the nuclear genomes. The *Ogura* sterile cytoplasm was first deployed in European cauliflowers^35^ and later on introgressed in Indian cauliflowers^26,38^. Still it is a robust and stable genetic system that ensures complete cross-pollination across the temperature range. The modification or alteration in the mitochondrial genome that causes male sterility may substantially impair CMS system performance. This has been documented in the ‘*Polima’* cytoplasm where partial restoration of fertility has occurred as a result of higher temperature^44^. As hybrid breeding in cauliflower currently solely depends on the *Ogura* CMS system, this will be a major calamity, if a *Polima*-like situation occurs. Thus, two additional sterile cytoplasms namely *E. canariense* (*Can*) and *B. tournefortii* Goun (*Tour*)^4,35^. This CMS introgression process might had an impact on other non-target but important traits such as agro-moprphological and floral traits. Because, normal size of flowers, properly developed petals, normal stigma and style and well-developed functional nectaries are required for drawing honey bees for pollination activities. The previous researchers have showed the negative effects of *Ogura* sterile cytoplasm on floral traits including flower size, petal length and width, stamens, stigma and nectary size as well as nectar volume^37,45–47^. These defects/deformities included reduced flower size, petaloid anthers, carpeloid stamens, malformed nectary, twisted stigma and split anthers in *Brassica* crops. A reduction in floral traits in CMS lines of Indian cauliflower carrying *Ogura*, *Can* and *Tour* sterile cytoplasm was also reported^22,38^. Size of functional nectaries is correlated with nectar volume^38,48^. And, the pollinator insects in *Brassica* crops are attracted to flowers based on the amount of nectar they produce ^49–51^.

In current investigation, the pattern of the per cent change in plant morphology, curd yield and floral traits revealed varied results. The parameters of PH, PS and leaf attributes showed a variable pattern. Maximum reduction in PH was recorded in DC 98-4 (−16.1%), while overall gain was seen in *Ogu* (DC-98-1) and *Can* (DC-7) in *Ogura* and *Can* cytoplasm-based CMS lines, respectively. Three CMS lines namely *Can* (DC-903) (12.0%), *Can* (DC-209) (7.5%) and *Can* (DC-18) (3.5%), which were considerably greater than other two CMS systems, demonstrated good gains in MCW. Dey et al.^37^ and Singh et al.^38^ both made similar observations. The MCY from *Can*-based CMS lines (21.8 t/ha) was nearly equal to that of the CMS lines carrying *Ogura* (21.8 t/ha) and *Tour* (21.4 t/ha) cytoplasm. This might be explained by nuclear–cytoplasmic interactions and cooperative functions between particular nuclear backgrounds of cauliflower and alien cytoplasms (i.e. sterile cytoplasm from other species). These alloplasmic lines, which carry cytoplasms namely *Can*, refined *Ogura* and *Tour* from three different sources—*E. canariense* (complete cytoplasm), *Raphanus sativus* L. (only mitochondria) and *B. tournifortti* (complete cytoplasm)*,* respectively*.* Refined *Ogura* based CMS lines carry chloroplast of *B. oleracea*, a unique functional combination in plant system.

The conversion procedure from fertile inbred lines to CMS lines had impact on floral features. Both FL, FD, PL and PW were decreased significantly. The findings are consistent with earlier studies in broccoli^46^, snowball cauliflower^37,48^ and Indian cauliflower^22^. The conversion process significantly decreased the size of the nectaries (NL, NW). The findings align with earlier reports^22,48^. NV also affected due to CMS trait. This might be the reason for a negative effect on seed yield in honey bee pollinated crops. The reduction in floral characteristics agreed with the findings of Dey et al.^41^ In comparison to their fertile lines, they reported a considerable decline in floral characteristics in refined *Ogura* based CMS lines of snowball cauliflower. However, all the *Can* and refined *Ogura* carrying CMS lines had decent size of flowers for honey bee visits (based on field observations of the senior author). Among the *Tour*-based CMS lines, only *Tour* (DC98-4) had a proper flower while the remaining *Tour* (DC-67) and *Tour* (DC-41-5) had most malformed flowers with ‘gripping phenomenon’ as also noted by Singh et al.^22^ The CMS phenotype is associated with abnormal recombination of mitochondria genome and CMS genes (*i.e. orf138* in *Ogura* CMS) encodes transmembrane proteins (ORF138) which bind to the mitochondrial membrane, affecting the hydrogen ion concentration gradient, thus affecting ATP synthesis. This leads to energy deficiency and excess accumulation of reactive oxygen species (ROS). The ROS play key role in cell signaling and their excess accumulation causes damage to lipids, proteins and DNA, inhibit enzyme activity, activate the programmed cell death (PCD) pathway and ultimately affect plant growth and development, particularly reproductive parts^52^.

The high seed yield in natural crossing conditions is essential for economic use of CMS system in hybrid breeding. The CMS trait impact most of floral traits which reflects in reduced seed yield also, however, in comparison, certain CMS systems have better performance than others^53^. The seed yield per plant in the refined *Ogura*-based CMS lines of snowball cauliflower namely Ogu1A, Ogu2A, and Ogu3A was reported to be 30.9 g, 21.4 g and 26.8 g per plant, respectively^54^. They reported that the seed yield per plant for these CMS lines was 24.3%, 29.3% and 10.1% less than its maintainer, respectively. Kucera et al.^55^ reported a reduction in seed yield per plant by 79.5% and 85.7% in FT CMS and BR CMS, respectively. It was attributed to fewer siliqua per plant and a smaller number of seeds per siliqua than their maintainers. The observed decrease in seed yield due to CMS trait in present study was consistent with the earlier findings^37,55^. However, this is the first in-depth account of changes brought on by *Can* sterile cytoplasm in agro-morphological and floral features of cauliflower. Similar to this, it was found that 11 *Can*-based CMS lines with the two pollen parents DC-67 and DC-98-4 produced hybrid seeds on average at rates of 19.5 g and 12.8 g/plant, respectively. Three *Ogura* based CMS lines with same pollen parents produced hybrid seeds 14.7 g and 18.5 g/plant, respectively. The *Tour*-based CMS lines, particularly *Tour *(DC-41-5) and *Tour *(DC 67) were worst performers for hybrid seed yield, mainly due to gripping phenomena of stigma^38^. High yield of hybrid seeds in present study could be due to genotype, open condition and nicking of CMS and pollen parents. Kucera et al.^55^ obtained only 2.3 g/plant hybrid seeds from BR CMS line with pollen parent FT13 grown in isolation cages provided with bumble bees. Thus, the study demonstrates the potential of these novel *Can* sterile cytoplasm carrying CMS lines in production of hybrid seeds.

The PCA and cluster analysis identified key agro-morphological and floral traits which contribute major share of variance in the CMS lines. The potential for trait comprehension for breeding purposes is indicated by the strong association between root and leaf parameters, curd weight and yield characteristics, and stamen length and nectary traits.

### mtDNA level molecular variation in CMS lines

At flowering stage, the CMS phenotype is persistent and simple to distinguish from fertile analogues. It is challenging to distinguish them at earlier stages, though. Further, the deployment of several sterile cytoplasms in cauliflower makes it challenging to distinguish these cytoplasms carrying CMS lines at a morphological level. Breeders will therefore benefit from the creation or discovery of DNA markers to differentiate between these several sterile cytoplasms. Additionally, because these markers are stage-independent, they can be applied to any specious mixture during the seedling stage or before flowering. Mitochondrial markers are thought to be a reliable method for identifying sterile and fertile cells as well as the type of cytoplasm that CMS lines carry^56,57^.

A dominant marker called ISSR3 was used by Wang and Song^58^ to distinguish fertile counterparts from the CMS lines. According to them, it is possible to distinguish between fertile and sterile lines using an amplicon of 313 bp size using a primer pair P9/P10 for mtDNA marker specific to the CMS cauliflower knxd612. Only two of the 25 mtDNA markers namely P14 and P15 were polymorphic between CMS and male fertile phenotypes. P16 also distinguished *Ogura* cytoplasm-based CMS line *Ogura* (DC-41-5) from its maintainer (DC-41-5). Notably, P14 distinguished *Tour* cytoplasm from *Ogura* and *canarianse* CMS systems which was in line with previous reports of Singh et al.^22^ Additionally, the polymorphism was found among the *Can*-based CMS lines. This might be the result of an adaptive interaction between the mitochondrial genome of *E*. *canerianse* and the nuclear genome of *B. oleracea* var. *botrytis* L. (cauliflower), which has changed the genetic makeup of the CMS lines. Such occurrences are uncommon, hence warrant for further studies because the modifications to the mitochondrial DNA do not occur so frequently (*i.e.* 6 to 7 years of introgression in case of *Can*—*oleracea* cytoplasm interaction) as observed from *Ogura*—*Brassica* interaction (nearly 25–30 years in existence). Also, further research is warranted to understand the variability among the CMS lines carrying same *Can* cytoplasm. This could be explained by the lower levels of polymorphism of target locus in the four cytoplasms studied (*Oleracea*, *Eru*, *Can* and *Tour*). Since these species share a common ancestor and except a few minor variations, their cytoplasmic genomes are still conserved to great extent.

The sequence analysis from five CMS lines including three *Can*-based [*Can* (DC-23), *Can* (DC-98-4), *Can* (DC-41-5)] and one each with *Ogura* [*Ogu* (DC-41-5)] and *Tour* cytoplasms [*Tour* (DC-41-5] along with their maintainers revealed insertions and deletions. This could explain the variations among these lines. Sequence analysis of P16 amplicon from *Can* (DC-98-4) and *Can* (DC-41-5) CMS lines had distinct bases from their maintainers. Further, sequence analysis of *Tour* (DC-41-5) CMS line revealed that P16 amplicon at 243^rd^ position and atpF-atpH amplicon at 138th position had T while rest of the lines had C and A, respectively. This indicates that flower deformity (i.e. gripping phenomenon’ could be due to this change in ORF region. However, it needs further investigation to establish the association. Primer atpF-atpH amplicon sequence revealed unique bases at manifold position indicating its distinctness from the other three cytoplasms. Earlier reports also suggest variation among the *Ogura*-cytoplasm carrying CMS lines of cauliflower^25^ and broccoli^31^. Deletions of nucleotide in the ORF coding region of broccoli cytolines was suggested to be associated with carpelloid stamen phenotype^31^, however, such abnormality was not reported in investigated CMS lines.

## Conclusion

The present work provides the first in-depth information on the effect of this sterile cytoplasm in new nucleo-cytoplasmic combination (*oleracea*-*canerianse*) lines of Indian cauliflower. *Erucastrum canarianse* Webb and Berthel is a new CMS system deployed in cauliflower. These lines contain the cauliflower nuclear genome, whereas *E. canarianse* provides the cytoplasm. Although most agro-morphological features and floral attributes changed as anticipated due to the novel cytoplasm-nuclear interaction, the impact’s size was not averse to the level which can affect pollination drastically. The introgression of *Can* CMS system in the tropical Indian cauliflower reduced flower parameters equal to those of the *Ogura* CMS line, but seed-setting capacity is adequate, emphasizing the potential for hybrid seed production. P14 and P15, two polymorphic co-dominant mtDNA markers that were also discovered for *Can* CMS system, will be helpful for early detection, tracking of change(s) in mtDNA sequences and spotting variation between CMS lines and their maintainers. Similarly, P16 was found to be useful to distinguish *Ogu* (DC-41-5) from its maintainer DC-41-5. In the instance of sequence analysis, the *Can*, *Ogu* and *Tour* cytoplasm harbouring CMS lines displayed variation. These novels *Can* CMS lines of Indian cauliflower creates the possibility for immediate use in hybrid breeding or further introgression in other members of *B. oleracea* group through backcrossing.

### Supplementary Information

Supplementary Information.

## Data Availability

All the data generated and analysed during this study are included in this published article and its supplementary information. However, additional information can be obtained from corresponding author on reasonable request.
