# Supplementary material for: Commercial suitability and characterization of newly developed Erucastrum canariense (Can) sterile cytoplasm based cytoplasmic male sterile (CMS) lines in Indian cauliflower
Source: Sci Rep. 2024 Jan 29;14:2346. doi: 10.1038/s41598-024-52714-z (PMC10822850; doi:10.1038/s41598-024-52714-z)
Supplement: Supplementary file 1 — Supplementary Information. [file 41598_2024_52714_MOESM1_ESM.docx]

Table S1. List of markers used for characterization of CMS lines of early cauliflower.

|  | Mt-DNA markers | Forward (5’---3’) | Reverse (5’---3’) | Annealing temperature (°C) | References |
| --- | --- | --- | --- | --- | --- |
| 1 | psbA-trnH | GTTATGCATGAACGTAATGCTC | CGCGCATGGTGGATTCACAATCC | 53 | Sang *et al*. (1997) |
| 2 | RpoC12 | GGCAAAGAGGGAAGATTTCG | CCATAAGCATATATCTTGAGTTGG | 53 | Sang *et al*. (1997) |
| 3 | ITS5a/ITS:4 | CCTTATCATTTAGAGGAAGGAG | TCCTCCGCTTATTGATATGC | 54 | White *et al*. (1990) |
| 4 | RpoB1 | AAGTGCATTGTTGGAACTGG | GATCCCAGCAGCATCACAATTCC | 53 | Sang *et al*. (1997) |
| 5 | Yef1b | TCTCGACGAAAATCAGATTGTTGTGAAT | ATACATGTCAAACTGATGGAAAA | 54 | Dong *et al*. (2015) |
| 6 | atpF-atpH | ACTCGCACACACTCCCTTTCC | GCTTTTATGGAAGCTTTAACAAT | 54 | Sang *et al*. (1997) |
| 7 | matK | CGTACAGTACTTTTGTGTTTACGAGC | ACCCAGTCCATCTGGAAATCTTGGTTC | 54 | Johnson and Soltis (1994) |
| 8 | rbeL | ATGTCACCACAAACAGAGACTAAAGC | GAAACGGTCTCTCCAACCCAT | 53 | Kress *et al*. (1997) |
| 9 | trnL | CGAAATCGGTAGACGCTACG | GGGGATAGAGGGACTTGAAC | 56 | Taberlet *et al*. (1991) |
| 10 | ITS-2 | ATGCGATACTTGGTGTGAAT | GACGCTTCTCCAGACTACAAT | 62 | Chen *et al*. (2010) |
| 11 | trnH-psbA | CGCGCATGGTGGATTCACAATCC | GTTATGCATGAACGTAATGCTC | 63 | Sang *et al*. (1997) |
| 12 | P1 | GAAACGGGAAGTGACAAT | GCATTATTTTCTCGGTCCAT | 54 | Shu *et al*. (2016) |
| 13 | P2 | AGCTGTCTGGAGGGAATC | GCGGTCTCACGCACTAATC | 56 | Shu *et al*. (2016) |
| 14 | P8 | GCAATGATTACCTTTTTCGA | GCATTATTTTCTCGGTCCCAT | 54 | Shu *et al*. (2016) |
| 15 | P9 | GAAACGGGAAGTGACAAT | GCATTATTTTCTCGGTCCAT | 54 | Shu *et al*. (2016) |
| 16 | P10 | CCATATTTGGCTAAGCTGGTTTTCT | TATTTTCTCGGTCCATTTTCCAC | 54 | Shu *et al*. (2016) |
| 17 | P11 | GCCCATATTTGGCTAATCTG | TTTTCTCGGTCCATTTTCCA | 62 | Shu *et al*. (2016) |
| 18 | P13 | AATGAAGCTGTCTGGAGGGA | TTCATTGAACTTCCATACCTG | 51 | Shu *et al*. (2016) |
| 19 | P16 | ACCAAGATTGAGCCAGAT | CGTCCTACTACCGAAAGAG | 54 | Shu *et al*. (2016) |
| 20 | P17 | CCCGAGAAGCACTGTTGA | ACGGAGTGACAAAGGAGC | 56 | Shu *et al*. (2016) |
| 21 | P18 | CCTTCTGGGTTGACTTGA | AGTGGTGCCCTCCTCTCTAA | 54 | Shu *et al*. (2016) |
| 22 | P19 | GCTGCTCATCACTACTACCTG | CACTACGCTCACTGAAACTA | 54 | Shu *et al*. (2016) |
| 23 | P12 | CGGTCGGTGTCCAAGATTT | ACTGTCGGGGTCGTGCTCT | 54 | Shu *et al*. (2016) |
| 24 | P15 | GAACTCCGAGGACCTTAGTACC | AGTAAGTTGTAGGTAGGGGCTTCAT | 54 | Shu *et al*. (2016) |
| 25 | P14 | CCGTTAGGGGTATTTAGTAACTCG | ACATAATGGCAATGTATCGGACTG | 54 | Shu *et al*. (2016) |


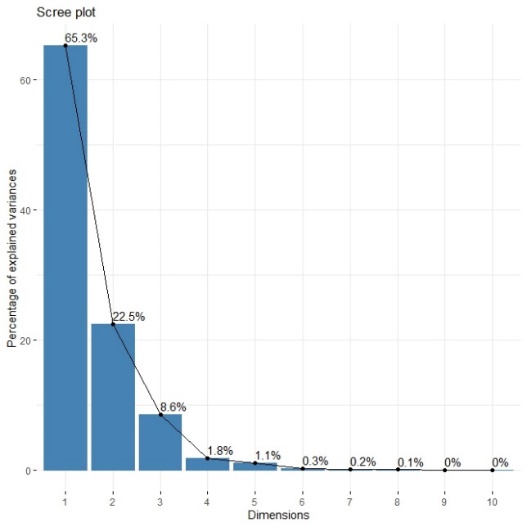

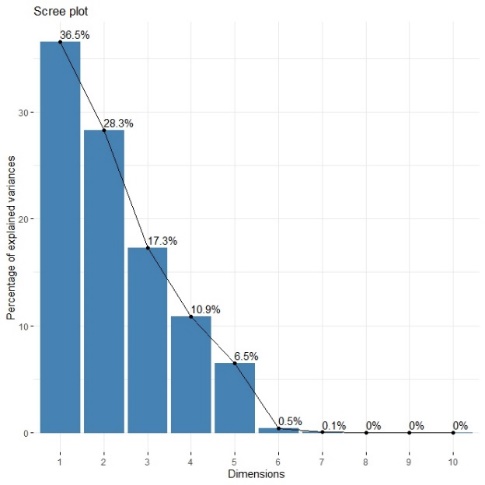


1. CMS lines b. Maintainers


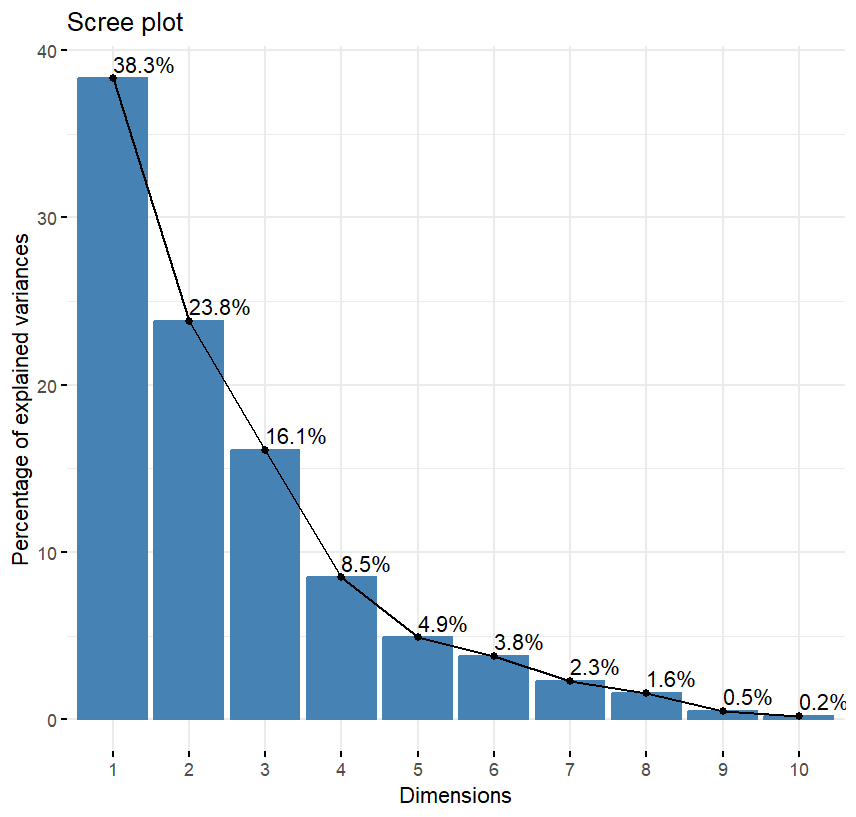

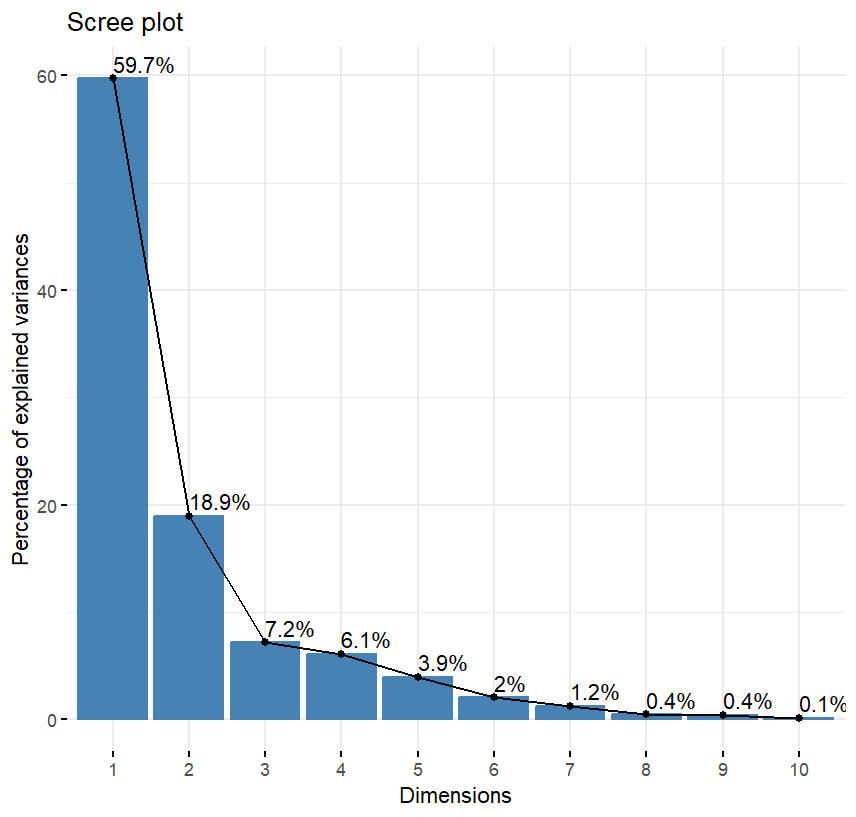


1. CMS lines d. Maintainers

Fig. S1a-d. Scree plot for agro-morphological and yield traits in CMS lines and maintainers (a-b) and floral traits (c-d) using "FactoMiner" R Package. Sl. No. 1-10 on X-axis represent principal components (PCs) for the respective groups.


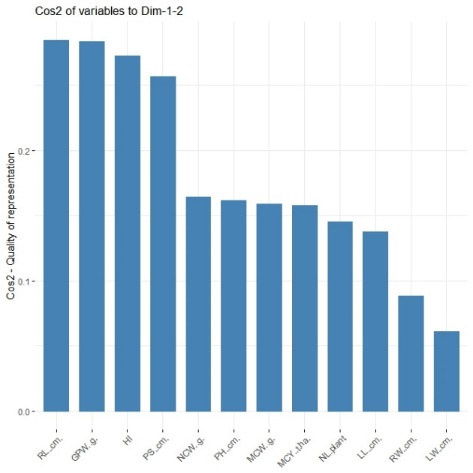

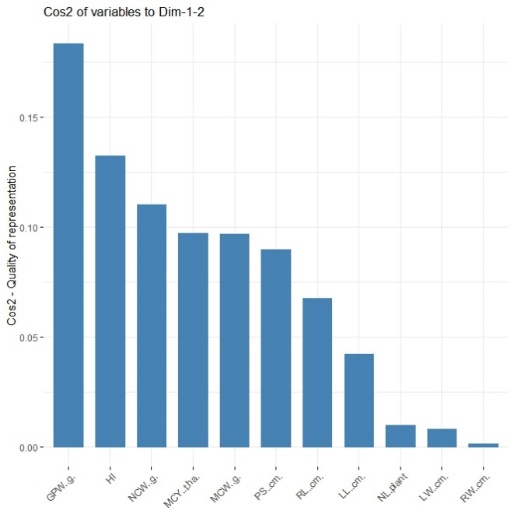


a. CMS lines B. Maintainers


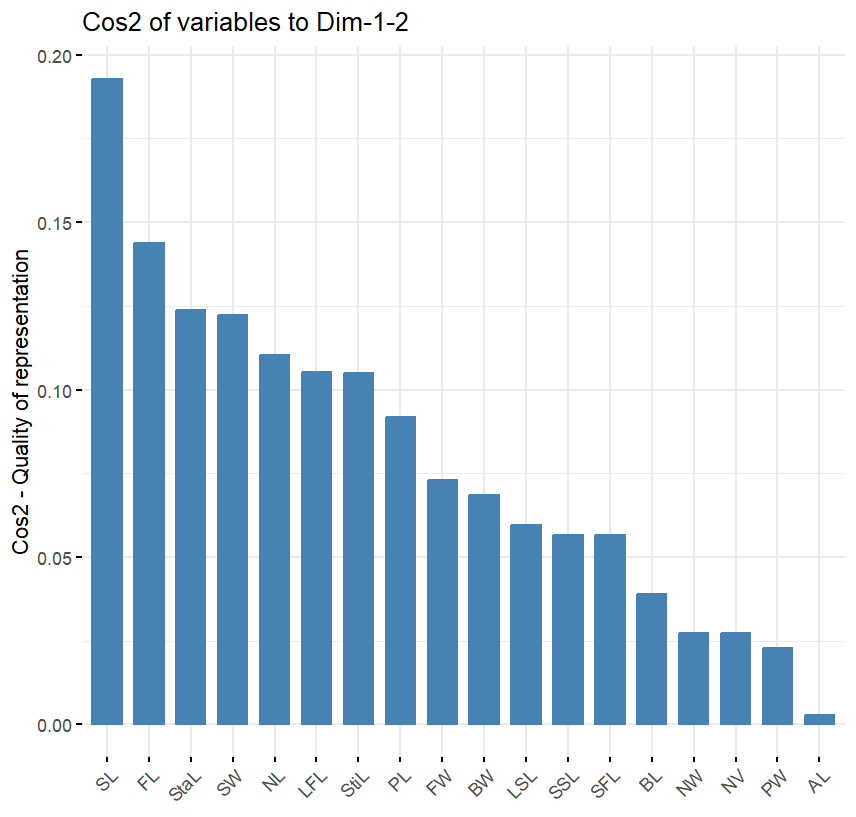

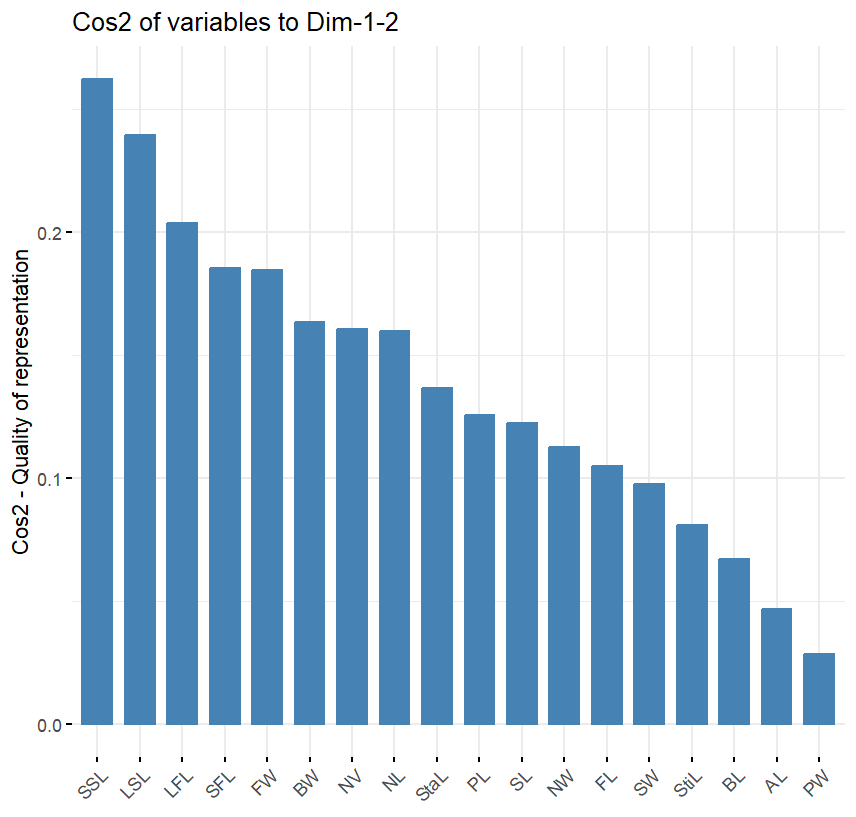


1. CMS lines d. Maintainers

Fig. S2a-d. Contribution of variables based PC1 and PC2 for agro-morphological and yield traits (a-b) and floral traits (c-d) in CMS lines and their maintainers using "FactoMiner" R Package. Here, PH- Plant height, PS-plant spread, LL- Leaf length, LW- Leaf width, NL – Number of leaves per plant, GPW – Gross plant weight, MCW- Marketable curd weight, NCW- Net curd weight, MCY- Marketable curd yield, RL-Root length, RW- root width and HI-Harvest Index, FL- Flower length, FD- Flower diameter, StaL- Stalk length, PL- Petal length, PW- Petal width, SL- Sepal length, SW- Sepal width, BL - Bud length, BW- Bud width, StiL- Stigma length, LSL- Long stamen length, SSL- Short stamen length, LFL- Long filament length, SFL- Short filament length, AL- Anther length, NL - Nectary length, NW- Nectary width, NV- Nectar volume.

| 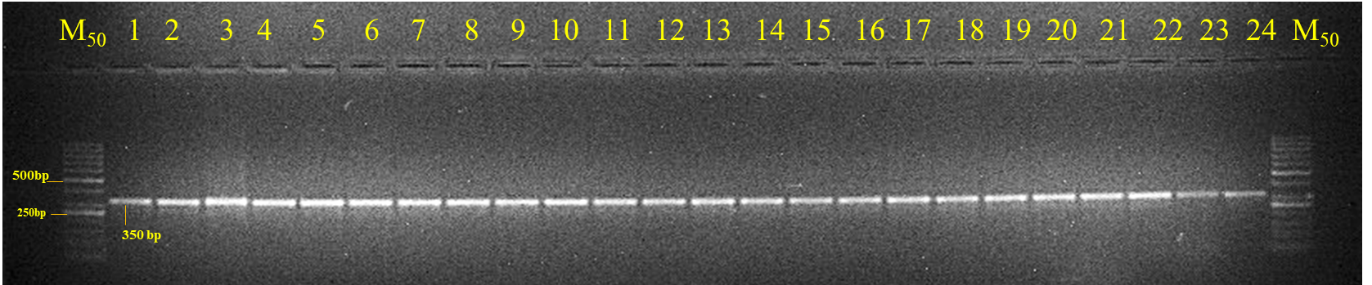 |
| --- |
| 1.psbA-trnH |
| 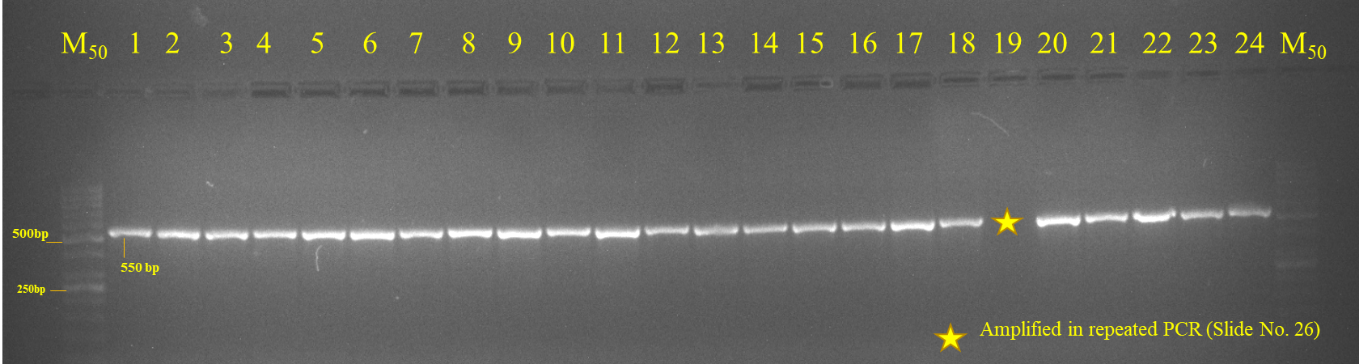 |
| 2. RpoC12 |
| 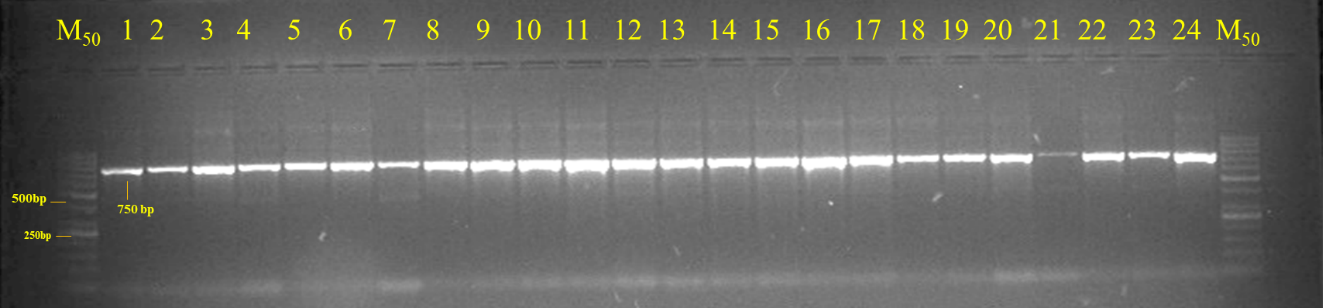 |
| 3.ITS5a/ITS:4 |
| 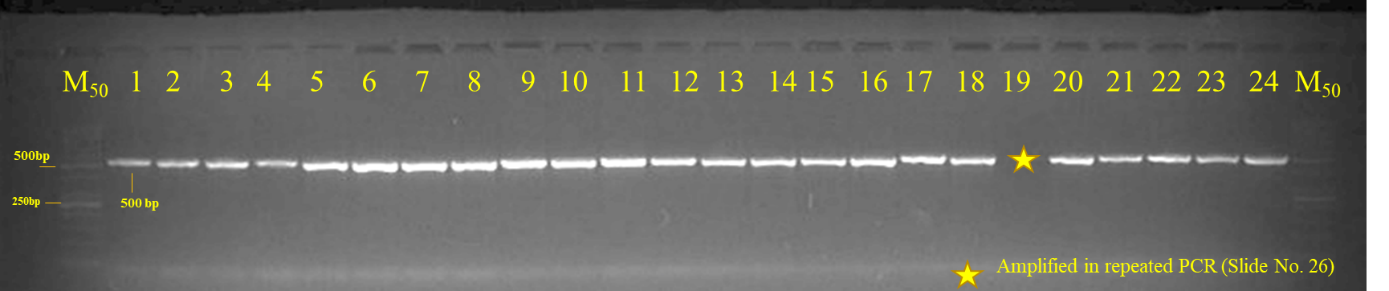 |
| 4. RpoB1 |
| 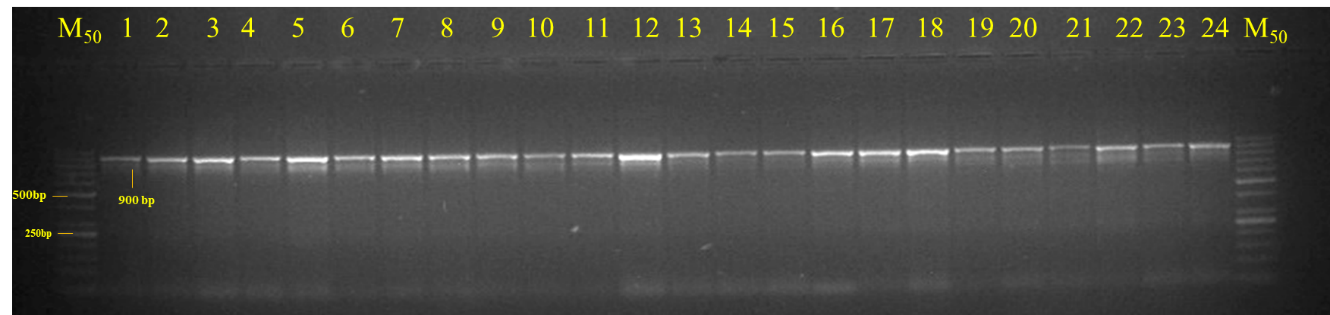 |
| 5.yef1b |
| 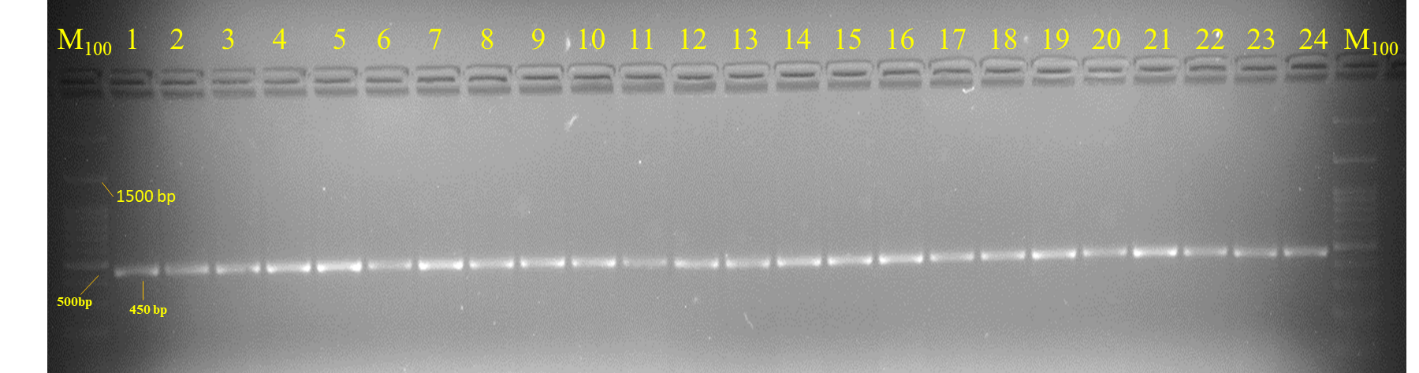 |
| 6.atpF-atpH |
| 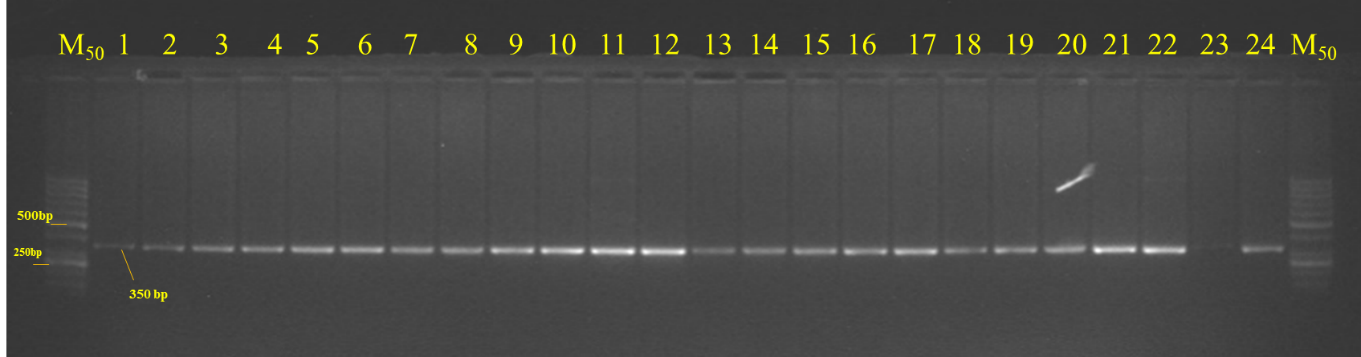 |
| 7. Matk |
| 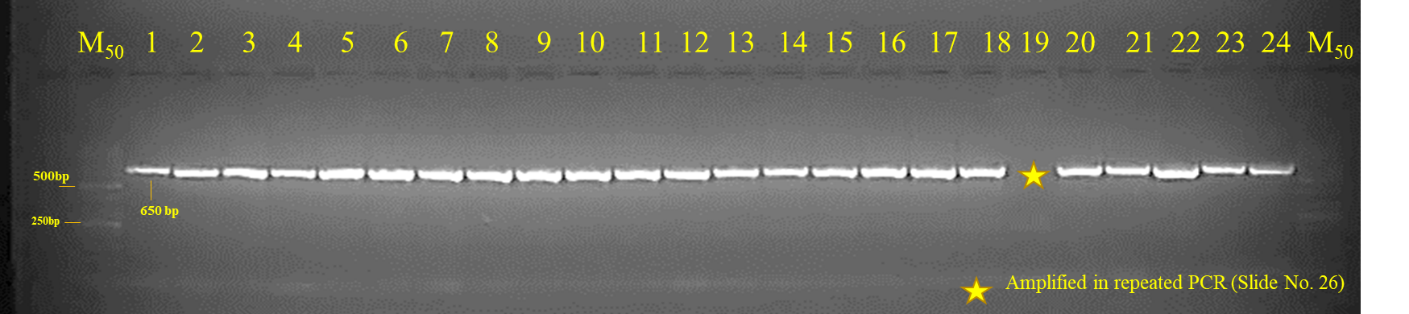 |
| 8. rbeL |
| 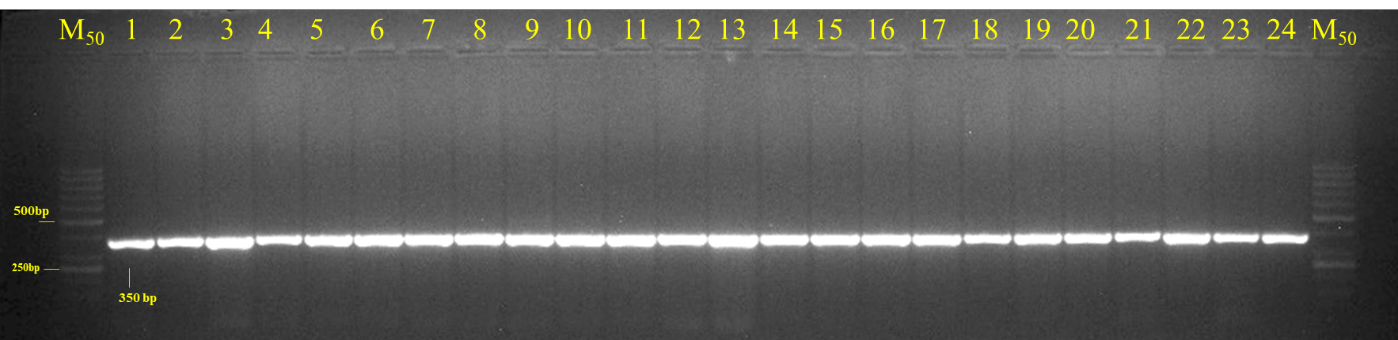 |
| 9.trnL |
| 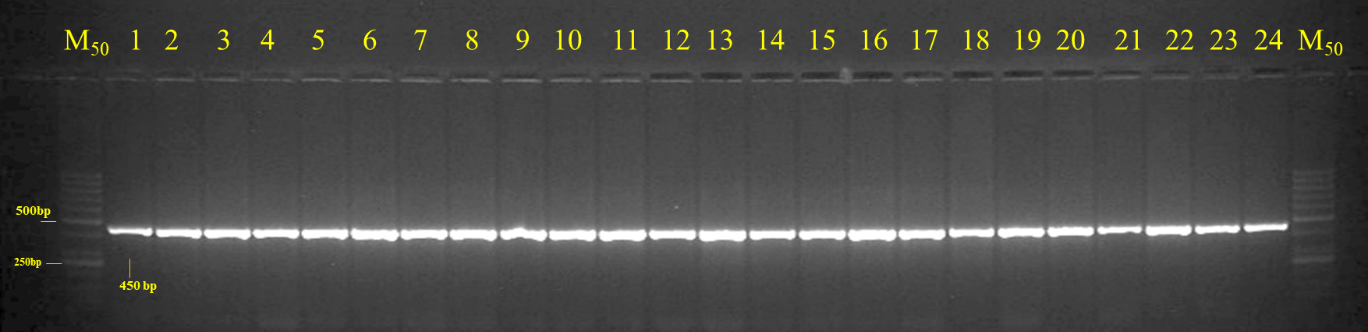 |
| 10.ITS-2 |
| 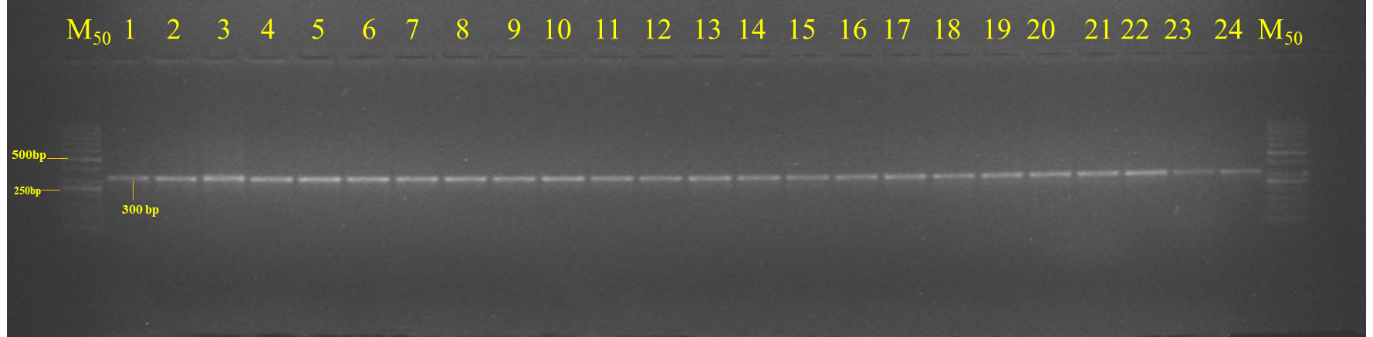 |
| 11. trnH-psbA |
| 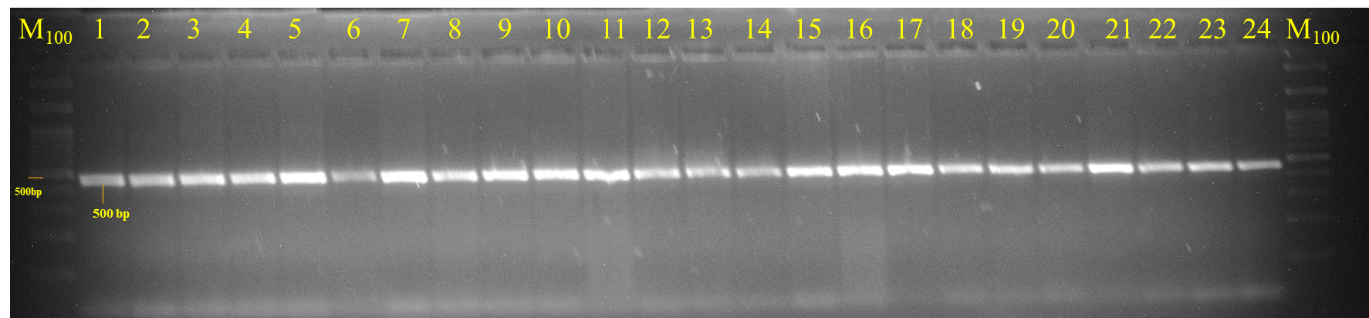 |
| 12. P1 |
| 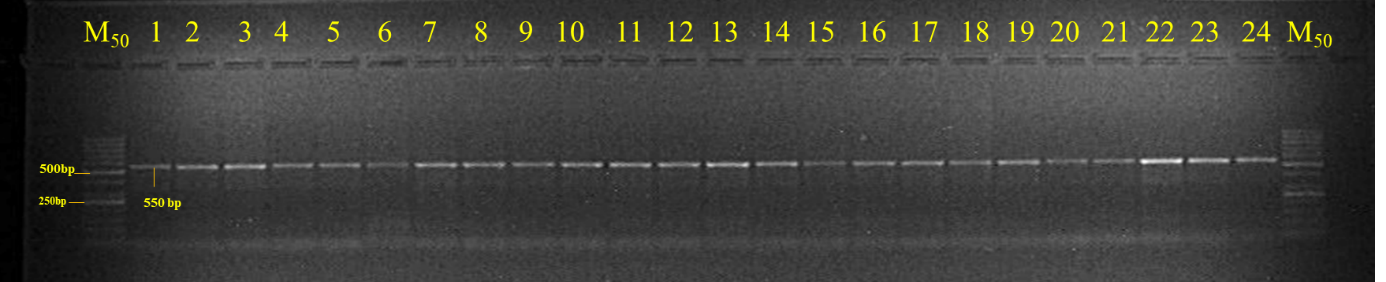 |
| 13. P2 |
| 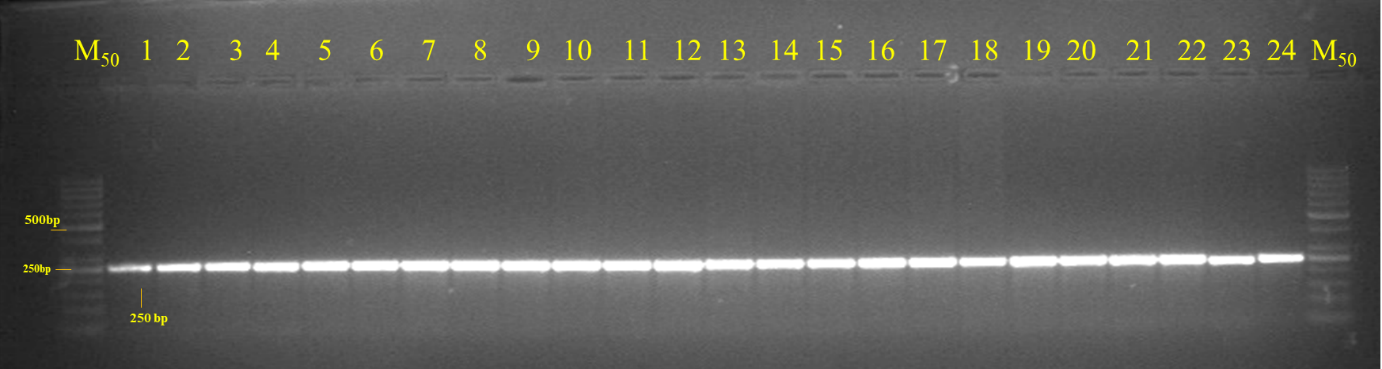 |
| 14. P8 |
| 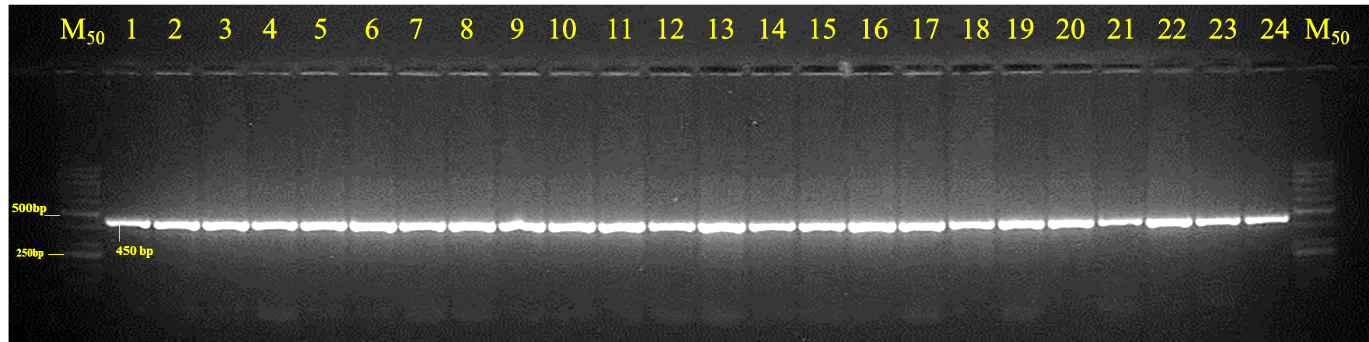 |
| 15.P9 |
| 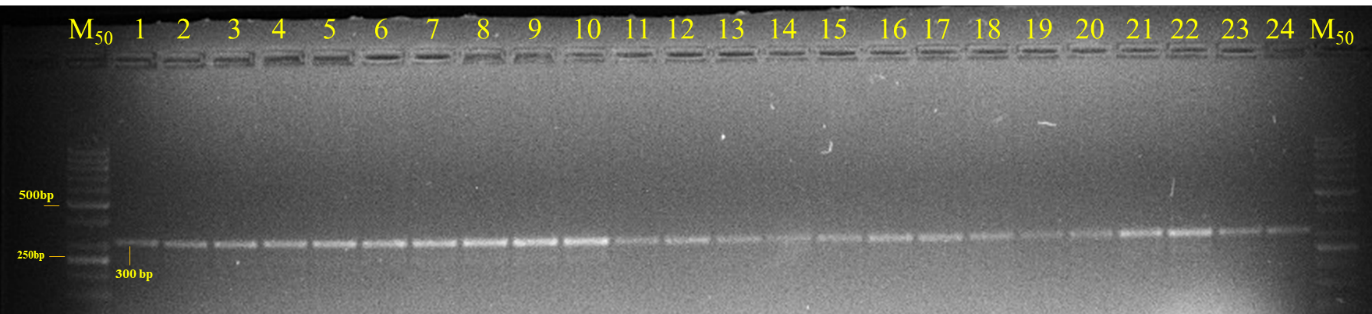 |
| 16. P10 |
| 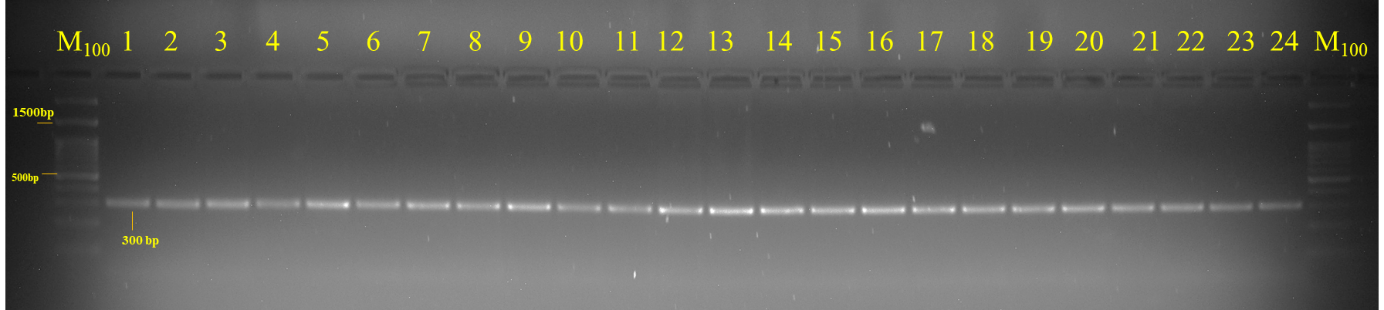 |
| 17. P 11 |
| 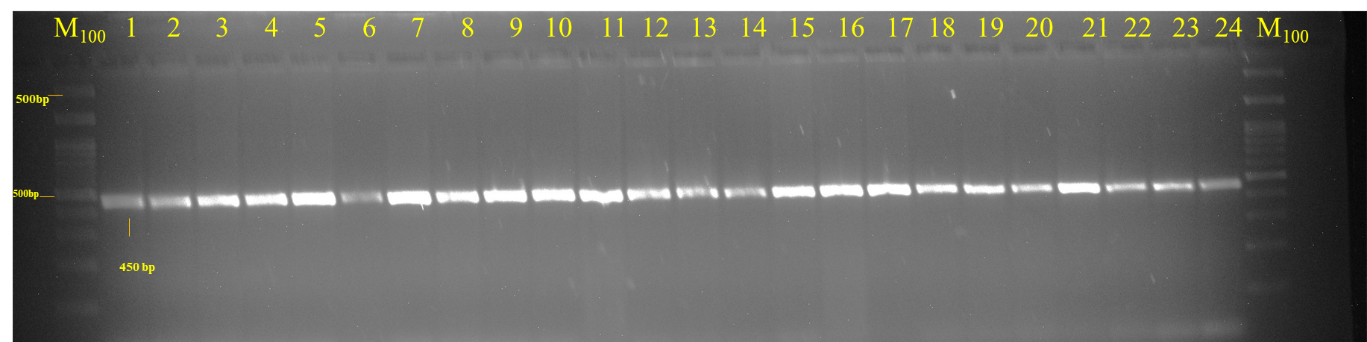 |
| 18. P 13 |
| 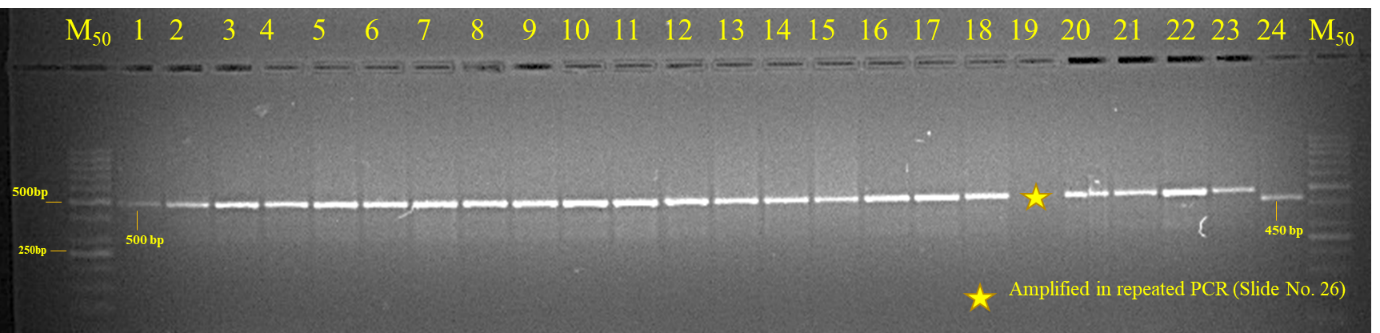 |
| 19.P16 |
| 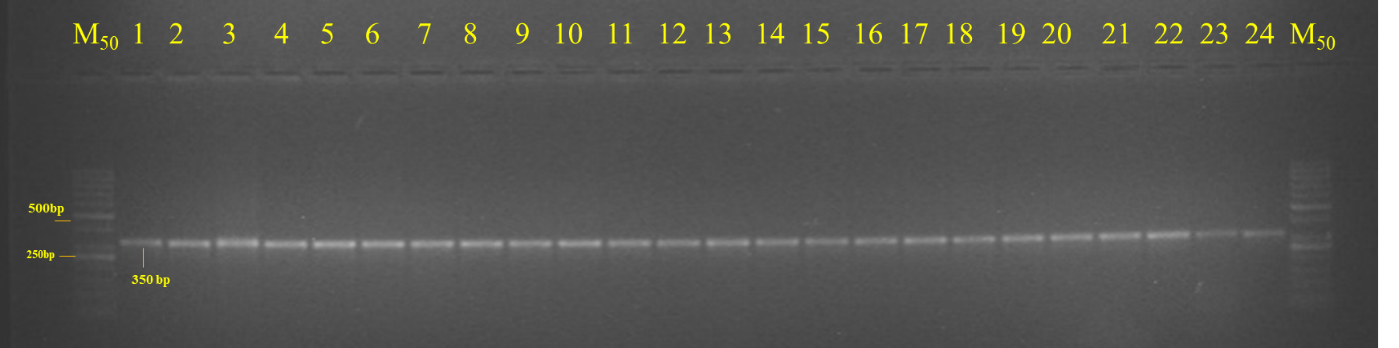 |
| 20. P 17 |
| 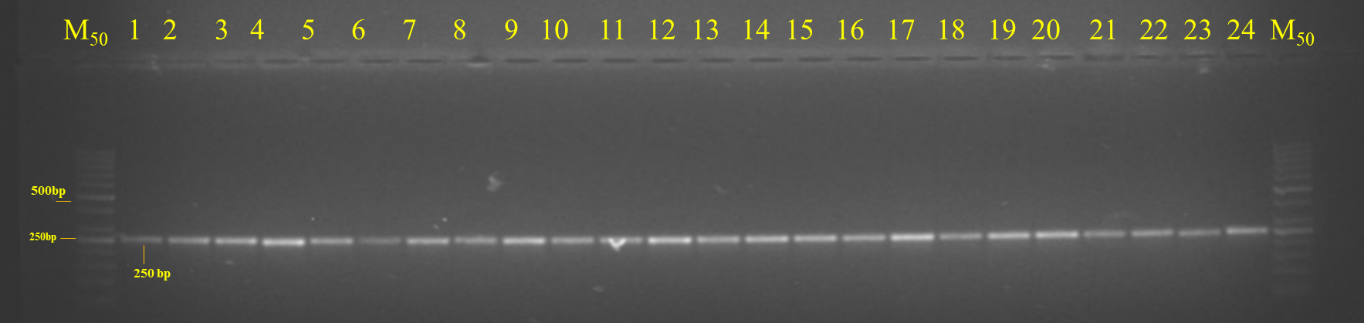 |
| 21.P18 |
| 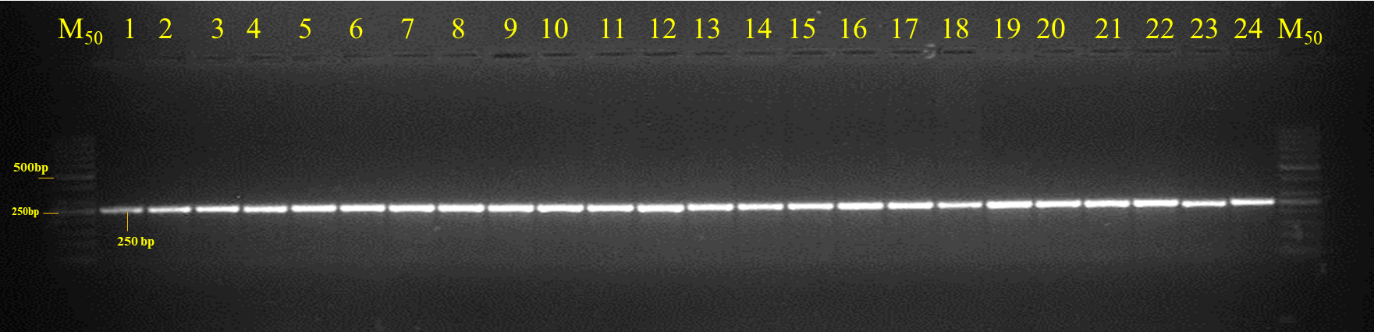 |
| 22.P19 |
| 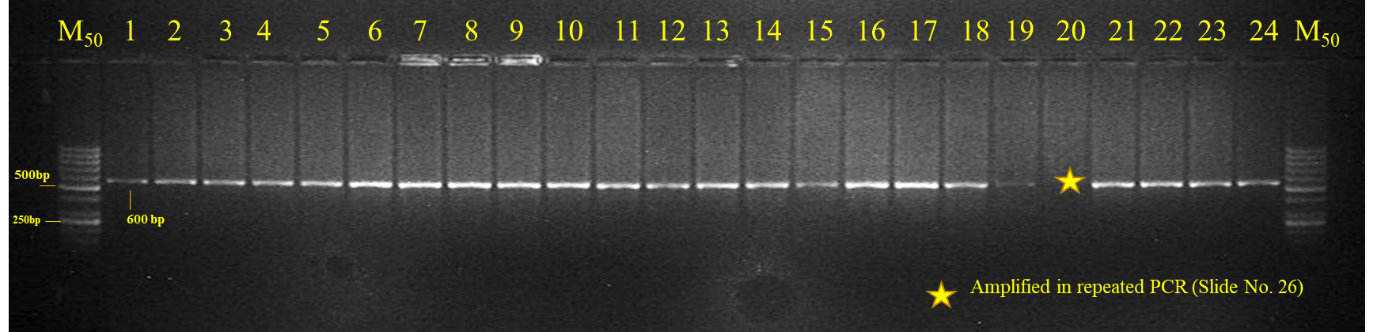 |
| 23.P12 |
| 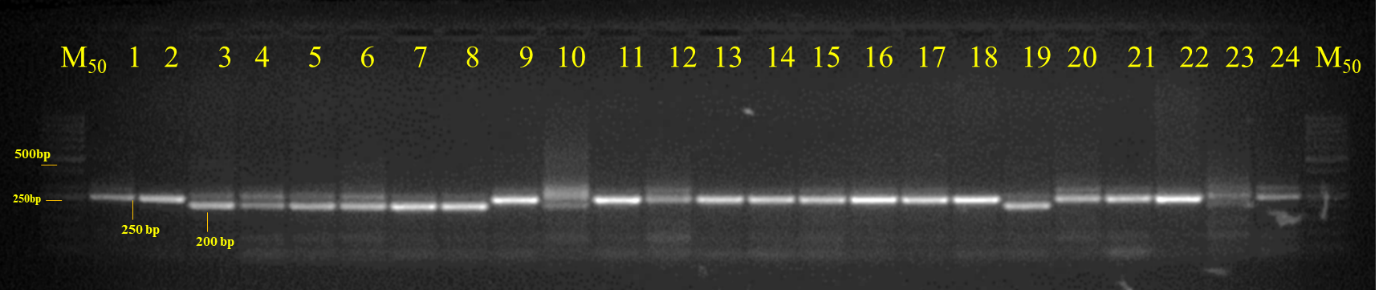 |
| 24.P15 |
| 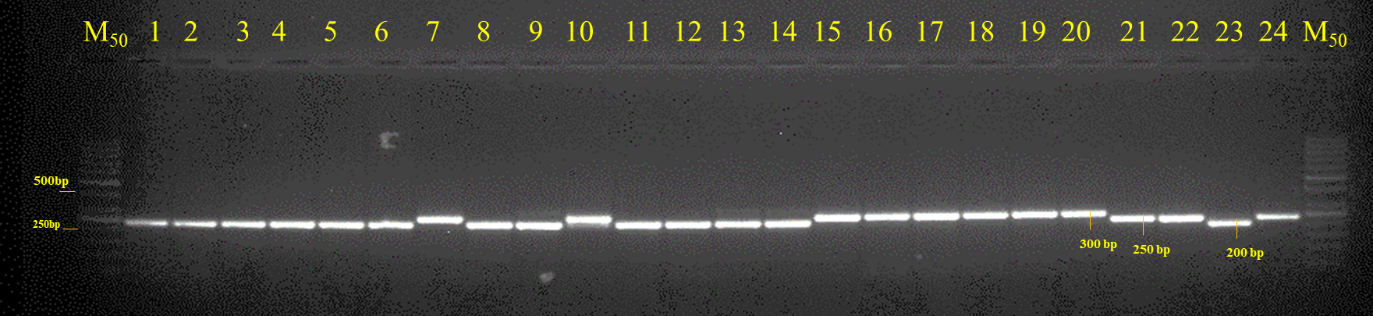 |
| 25.P14 |
| 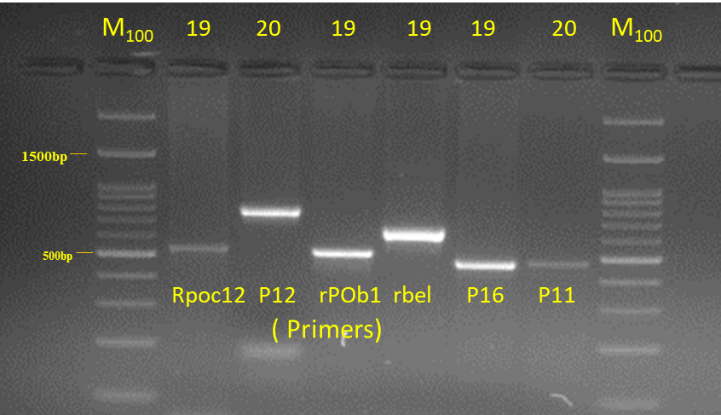 |
| 26. Repeated PCR amplification of earlier not–amplified primers (Rpoc12, P12, rPOb1, rbel, P16 and P11) |

Fig. S3. Amplification of *Can*, *Ogu* and *Tour* CMS lines in early cauliflower and their maintainers using mtDNA markers. Sl. No. 1-24 are:

Sl. No. 1-24 are as follows: 1. *Can*(DC-23) 2. *Can*(DC-98-4) 3. *Can*(DC-7) 4. *Can*(DC-8) 5. *Can*(DC-94-2 6. *Can*(DC-903), 7*.Can*(DC-209) 8. *Can*(DC-41-5) 9*.Can*(DC-18) 10. *Can*(DC-63) 11.DC-23 12. DC-98-4 13. DC-7 14.DC-8 15. DC-94-2 16.DC-903 17. DC-209 18. DC-41-5 19. DC-18 20. DC-63, 21. *Ogu*(DC-98-4) 22. *Ogu*(DC-67) 23. *Tour*(DC-41-5) 24. *Ogu*(DC-41-5). M50- Ladder 50 bp and MB_100_ ladder 100 bp (GeneDireX)

10 20 30 40 50

....|....| ....|....| ....|....| ....|....| ....|....|

*Can*(DC-23) TTGGGTCATC AAGAGCTTTC GGACCGAAAC GACTGACGAT TTGACGAGAA

DC-23 TTGGGTCATC AAGAGCTTTC GGACCGAAAC GACTGACGAT TTGACGAGAA

DC-*41-5* TTGGGTCATC AAGAGCTTTC GGACCGAAAC GACTGACGAT TTGACGAGAA

*Ogu*(*41-5)* TTGGGTCATC AAGAGCTTTC GGACCGAAAC GACTGACGAT TTGACGAGAA

*Can*(98-4) TTGGGTCATC AAGAGCTTTC GGACCGAAAC GACTGACGAT TTGACGAGAA

DC-98-4 TTGGGTCATC AAGAGCTTTC GGACCGAAAC GACTGACGAT TTGACGAGAA

*Can*(*41-5*) TTGGGTCATC AAGAGCTTTC GGACCGAAAC GACTGACGAT TTGACGAGAA

*Tour*(*41-5*) TTGGGTCATC AAGAGCTTTC GGACCGAAAC GACTGACGAT TTGACGAGAA

60 70 80 90 100

....|....| ....|....| ....|....| ....|....| ....|....|

*Can(DC-23)*  TTGAATTCAC CACCGCATGT CAAGACGCTC CTGGCATCCT TAGCTCGGAT

*DC-23* TTGAATTCAC CACCGCATGT CAAGACGCTC CTGGCATCCT TAGCTAGGAT

*DC-41-5* TTGAATTCAC CACCGCATGT CAAGACGCTC CTGGCATCCT TAGCTCGGAT

*Ogu(41-5)* TTGAATTCAC CACCGCATGT CAAGACGCTC CTGGCATCCT TAGCTCGGAT

*Can(98-4)* TTGAATTCAC CACCGCATGT CAAGACGCTC CTGGCATCCT TAGCTCGGAT

*DC-98-4* TTGAATTCAC CACCGCATGT CAAGACGCTC CTGGCATCCT TAGCTAGGAT

*Can*(41-5) TTGAATTCAC CACCGCATGT CAAGACGCTC CTGGCATCCT TAGCTCGGAT

*Tour(41-5)* TTGAATTCAC CACCGCATGT CAAGACGCTC CTGGCATCCT TAGCTCGGAT

110 120 130 140 150

....|....| ....|....| ....|....| ....|....| ....|....|

*Can(DC-23)*  TTTGGCCAAC CGCGTGCGGT AACACACGGG AGACCAGCTT CCGTCCGATA

*DC-23* TTTGGCCAAC CGCGTGCGGT AACACACGGG AGACCAGCTT CCGTCCGATA

*DC-41-5* TTTGGCCAAC CGCGTGCGGT AACACACGGG AGACCAGCTT CCGTCCGATA

*Ogu(41-5)* TTTGGCCAAC CGCGTGCGGT AACACACGGG AGACCAGCTT CCGTCCGATA

*Can(98-4)* TTTGGCCAAC CGCGTGCGGT AACACACGGG AGACCAGCTT CCGTCCGATA

*DC-98-4* TTTGGCCAAC CGCGTGCGGT AACACACGGG AGACCAGCTT CCGTCCGATA

*Can*(41-5) TTTGGCCAAC CGCGTGCGGT AACACACGGG AGACCAGCTT CCGTCCGATA

*Tour(41-5*) TTTGGCCAAC CGCGTGCGGT AACACACGGG AGACCAGCTT CCGTCCGATA

160 170 180 190 200

....|....| ....|....| ....|....| ....|....| ....|....|

*Can(DC-23)* TCCTCGAGAG GATTGGGGGA CGACGATTTG TGACACCCAG GCAGACGTGC

*DC-23* TCCTCGAGAG GATGGGGGGA CGACGATTTG TGACACCCAG GCAGACGTGC

*DC-41-5* TCCTCGAGAG GATTGGGGGA CGACGATTTG TGACACCCAG GCAGACGTGC

*Ogu(41-5)* TCCTCGAGAG GATTGGGGGA CGACGATTTG TGACACCCAG GCAGACGTGC

*Can(98-4)* TCCTCGAGAG GATTGGGGGA CGACGATTTG TGACACCCAG GCAGACGTGC

*DC-98-4*) TCCTCGAGAG GATGGGGGGA CGACGATTTG TGACACCCAG GCAGACGTGC

*Can*(41-5) TCCTCGAGAG GATTGGGGGA CGACGATTTG TGACACCCAG GCAGACGTGC

*Tour(41-5*) TCCTCGAGAG GATTGGGGGA CGACGATTTG TGACACCCAG GCAGACGTGC

210 220 230 240 250

....|....| ....|....| ....|....| ....|....| ....|....|

*Can(DC-23)* CCTCGGCCAG AAGGCTTGGG GCGCAACTTG CGTTCAAAGA CTCGATGGTT

*DC-23* CCTCGGCCAG AAGGCTTGGG GCGCAACTTG CGTTCAAAGA CTCGATGGTT

*DC-41-5* CCTCGGCCAG AAGGCTTGGG GCGCAACTTG CGTTCAAAGA CTCGATGGTT

*Ogu(41-5)* CCTCGGCCAG AAGGCTTGGG GCGCAACTTG CGTTCAAAGA CTCGATGGTT

*Can(98-4)* CCTCGGCCAG AAGGCTTGGG GCGCAACTTG CGTTCAAAGA CTCGATGGTT

*DC-98-4* CCTCGGCCAG AAGGCTTGGG GCGCAACTTG CGTTCAAAGA CTCGATGGTT

*Can*(41-5) CCTCGGCCAG AAGGCTTGGG GCGCAACTTG CGTTCAAAGA CTCGATGGTT

*Tour(41-5)* CCTCGGCCAG AAGGCTTGGG GCGCAACTTG CGTTCAAAGA CTCGATGGTT

260 270 280 290 300

....|....| ....|....| ....|....| ....|....| ....|....|

*Can(DC-23)* CACGGGATTC TGCAATTCAC ACCAAGTATC GCATTTCGCT ACGTTCTTCA

*DC-23* CACGGGATTC TGCAATTCAC ACCAAGTATC GCATTTCGCT ACGTTCTTCA

*DC-41-5* CACGGGATTC TGCAATTCAC ACCAAGTATC GCATTTCGCT ACGTTCTTCA

*Ogu(41-5)* CACGGGATTC TGCAATTCAC ACCAAGTATC GCATTTCGCT ACGTTCTTCA

*Can(98-4)* CACGGGATTC TGCAATTCAC ACCAAGTATC GCATTTCGCT ACGTTCTTCA

*DC-98-4*) CACGGGATTC TGCAATTCAC ACCAAGTATC GCATTTCGCT ACGTTCTTCA

*Can*(41-5) CACGGGATTC TGCAATTCAC ACCAAGTATC GCATTTCGCT ACGTTCTTCA

*Tour(41-5)* CACGGGATTC TGCAATTCAC ACCAAGTATC GCATTTCGCT ACGTTCTTCA

310 320 330 340 350

....|....| ....|....| ....|....| ....|....| ....|....|

*Can(DC-23)* TCGATGCGAG AGCCGAGATA TCCGTTGCCG AGAGTCGTTT TAGACTTTAC

*DC-23* TCGATGCGAG AGCCGAGATA TCCGTTGCCG AGAGTCGTTT TAGACTTTAC

*DC-41-5* TCGATGCGAG AGCCGAGATA TCCGTTGCCG AGAGTCGTTT TAGACTTTAC

*Ogu(41-5)* TCGATGCGAG AGCCGAGATA TCCGTTGCCG AGAGTCGTTT TAGACTTTAC

*Can(98-4)* TCGATGCGAG AGCCGAGATA TCCGTTGCCG AGAGTCGTTT TAGACTTTAC

*DC-98-4* TCGATGCGAG AGCCGAGATA TCCGTTGCCG AGAGTCGTTT TAGACTTTAC

*Can*(41-5) TCGATGCGAG AGCCGAGATA TCCGTTGCCG AGAGTCGTTT TAGACTTTAC

*Tour(41-*5) TCGATGCGAG AGCCGAGATA TCCGTTGCCG AGAGTCGTTT TAGACTTTAC

360 370 380 390 400

....|....| ....|....| ....|....| ....|....| ....|....|

*Can(DC-23)* ATTGCAGCAC TGCTTCCGAA CAAACACCGT CTCCGGGTTG GCGAAAGCAG

*DC-23* ATTGCAGCAC TGCTTCCGAA CAAACACCGT CTCCGGGTTG GCGAAAGCAG

*DC-41-5* ATTGCAGCAC TGCTTCCGAA CAAACACCGT CTCCGGGTTG GCGAAAGCAG

*Ogu(41-5)* ATTGCAGCAC TGCTTCCGAA CAAACACCGT CTCCGGGTTG GCGAAAGCAG

*Can(98-4)* ATTGCAGCAC TGCTTCCGAA CAAACACCGT CTCCGGGTTG GCGAAAGCAG

*DC-98-4* ATTGCAGCAC TGCTTCCGAA CAAACACCGT CTCCGGGTTG GCGAAAGCAG

*Can*(41-5) ATTGCAGCAC TGCTTCCGAA CAAACACCGT CTCCGGGTTG GCGAAAGCAG

*Tour(41-5)* ATTGCAGCAC TGCTTCCGAA CAAACACCGT CTCCGGGTTG GCGAAAGCAG

410 420 430 440 450

....|....| ....|....| ....|....| ....|....| ....|....|

*Can(DC-23)* GCTGTTTAGT TGAATGTTCC TTGACACTTT TCGTGCCGGG GTTTGGTGAT

*DC-23* GCCGTTTAGT TGAATGTTCC TTGACACTTT TCGTGCCGGG GTTTGGTGAT

*DC-41-5* GCTGTTTAGT TGAATGTTCC TTGACACTTT TCGTGCCGGG GTTTGGTGAT

*Ogu(41-5)* GCTGTTTAGT TGAATGTTCC TTGACACTTT TCGTGCCGGG GTTTGGTGAT

*Can(98-4)* GCTGTTTAGT TGAATGTTCC TTGACACTTT TCGTGCCGGG GTTTGGTGAT

*DC-98-4* GCCGTTTAGT TGAATGTTCC TTGACACTTT TCGTGCCGGG GTTTGGTGAT

*Can*(41-5) GCTGTTTAGT TGAATGGTCC TTGACACTTT TCCTGCCGGG GTTTGGTGAT

*Tour(41-5)* GCTGTTTAGT TGAATGTTCC TTGACACTTT TCGTGCCGGG GTTTGGTGAT

460 470 480 490 500

....|....| ....|....| ....|....| ....|....| ....|....|

*Can(DC-23)* ATCCGGAAGC TATGCGTACA ATCCAACCGA AACTGAGCCG GTGACGAACG

*DC-23* ATCCGGAAGC TATGCGTATG ATCCAACCGA AACTGAGCCG GTGACCAACG

*DC-41-5* ATCCGGAAGC TATGCGTACA ATCCAACCGA AACTGAGCCG GTGACGAACG

*Ogu(41-5)* ATCCGGAAGC TATGCGTACA ATCCAACCGA AACTGAGCCG GTGACCAACG

*Can(98-4)* ATCCGGAAGC TATGCGTACG ATCCAACCGA AACTGGGCCG GTGACGAACG

*DC-98-4* ATCCGGAAGC TATGCGTATG ATCCAACCGA AACTGGGCCG GGGACGAACG

*Can*(41-5) ATCCGGAAGC TATGCGGATA ATCCAACCGA AACTGAGCCG GGGGGCAAAG

*Tour(41-5)* ATCCGGAAGC TATGCGTACA ATCCAACCGA AACTGAGCCG GTGACCAACG

510 520 530 540 550

....|....| ....|....| ....|....| ....|....| ....|....|

*Can(DC-23)* CATAACCACG GAA-TCGGTA GGCACGAAAT CAGCTAAGAT ACCGGCCCAC

*DC-23* CATAACCACC GGA-TCTGTA GGGACGAAAT CAGCTAAGAA ACCGGCCCAC

*DC-41-5* CATAACCACG GAA-TCTGTA AGCACGAAAT CAGCTAAGAT ACCGGCCCAC

*Ogu(41-5)* CATAACCACG GAA-TCTGTA GGCACGAAAT CAGCTAAGAT ACCGGCCCAC

*Can(98-4)* CATAACCACG GAA-TCGGTA GGCACGAAAT CAGCTAAGAT ACCGGCCCAC

*DC-98-4* CATAACCACG GAA-TCGGGA GGGACGAAAT CAGCTAAGAA ACCGGCCCAC

*Can*(41-5) CCTTAACCCC GGA-TTTGGA AGGACGAAAT CCCCTTAGAA AGCGGACCCC

*Tour(41-5)* CATAACCACG GAA-TCTGTA GGCACGAAAT CAGCTAAGAT ACCGGCCCAC

560 570 580 590 600

....|....| ....|....| ....|....| ....|....| ....|....|

*Can(DC-23)* CGAGAGTGAT GTTTCAACGT TCT--CGGGT CGTTCTGTTT CCAGGTTACG

*DC-23* CGAGAGTGAT GTTTCA-CGT TCT--CGGGT CGTTCTGTTT CCAGGTTACG

*DC-41-5* CGAGAGTGAT GTTTCAACGT TCT--CGGGT CGTTCTGTTT CCAGGTTACG

*Ogu(41-5)* CGAGAGTGAT GTTTCAACGT TCT--CGGGT CGTTCTGTTT CCAGGTTACG

*Can(98-4)* CGAGAGTGAT GTTTCAACGT TCT--CGGGT CGTTCTGTTT CCAGGTTACG

*DC-98-4* CGAGAGTGAT GTTTCAACGT TCT--CGGGT CGTTCTGTTT CCAGGTTACG

*Can*(41-5) CCAAAGTGAA GTTTCCAAGT TTTTCCGGGC CGAAG-GTTC CCGGGGACCA

*Tour(41-5)* CGAGAGTGAT GTTTCAACGT TCT--CGGGT CGTTCTGTTT CCAGGTTACG

610

....|....| ..

*Can(DC-23)* ACAATGATCC TT

*DC-23* ACA-TGATCC TT

*DC-41-5* ACAATGATCC TT

*Ogu(41-5)* ACAATGATCC TT

*Can(98-4)* ACAATGATCC TT

*DC-98-4* ACAATGATCC TT

*Can*(41-5) AAAATGTTCT TC

*Tour(41-5)* ACAATGATCC TT

Fig.S4a. Alignment pattern of amplicon sequences obtained from primer ITS 5a-ITS 4.

10 20 30 40 50

....|....| ....|....| ....|....| ....|....| ....|....|

*Can(DC-23)* GTTTTTTTGA ATAAAAAGAA AAATT-CGTT TAATAATTTA -TAATTTAAT

*DC-23* GTTTTTTTGA ATAAAAAGAA AAATT-CGTT TAATAATTTA -TAATTTAAT

*Can(98-4)* GTTTTTTTGA AAAAAAAGAA AAATTTCGTT TAAAAATTTA -TAATTTAAT

*DC-98-4* GTTTTTTTGA ATAAAAAAAA AAATTTCGTT TAAAAATTTA -TAATTTAAA

*Can*(41-5) GTTTTTTTGA ATAAAAAAAA AAATTTCGTT TAAAAATTTA -TAATTTAAT

*Tour(41-5)* GTTTTTTTGA ATAAAAAGAA AAATT-CGTT TAATAATTTA -TAATTTAAT

*Ogu*(41-5) GCTTTTTTCC GAAAAAAAAA AATTTTCGTT AAAAGGTAAA ATAATTTTAT

*DC-41-5* GTTTTTTTGA ATAAAAAGAA AAATTTCGTT TAAAAATTTA -TAATTTAAT

60 70 80 90 100

....|....| ....|....| ....|....| ....|....| ....|....|

*Can(DC-23)* TTACC-TATT TGGATATTTA -TAAACAGAA TCCAAAACCT ATTCTATTT-

*DC-23* TTACC-TATT TGGATATTTA -TAAACAGAA TCCAAAACCT ATTCTATTT-

*Can(98-4)* TTTCCCTATT GGGATATTTA ATAAACAAAA TCAAAAACCT ATTCTATTT-

*DC-98-4* TTACC-TATT GGGATATTGA -TAAACAAAA TCAAAAACCT ATTCTATTT-

*Can*(41-5) TTACC-TATT TGGATATTTA -TAAACAAAA TCAAAAACCT ATTCTATTTT

*Tour(41-5)* TTACC-TATT TGGATATTTA -TAAACAGAA TCCAAAACCT ATTCTATTT-

*Ogu*(41-5) TGGCC-TTTT GGGATTTTTT GGGGGGTAAG GGTTTAACCA AATTCCTTTT

*DC-41-5* TTACC-TATT TGGATATTTA -TAAACAGAA TCAAAAACCT ATTCTATTT-

110 120 130 140 150

....|....| ....|....| ....|....| ....|....| ....|....|

*Can(DC-23)* TCAAATTTAG TTT-CCAAAA ATTTTTA-AT TTTCAATAAT AATAATGAGA

*DC-23* TCAAATTTAG TTT-CCAAAA ATTTTTA-AT TTTCAATAAT AATAATGAGA

*Can(98-4)* ACAAATTTAG TTTTCCAAAA ATTTTTA-AT TTTCAATAAT AATAAGGAAA

*DC-98-4* ACAAATTTAG TTT-CCAAAA ATTTTTA-AT TTTCAATAAT AATAAGGAAA

*Can*(41-5) ACAAATTTAG TTT-CCAAAA ATTTTTA-AT TTTCAATAAA AATAAGGAAA

*Tour(41-5)* TCAAATTTAG TTT-CCAAAA ATTTTTA-AT TTTCAATTAT AATAATGAGA

*Ogu*(41-5) ACAAATTGGG TCCCCCAAAA ATTTTTATAT TTTCAAAAAA ACCATTGGGA

*DC-41-5* ACAAATTTAT TTT-CCAAAA ATTTTTA-AT TTTCAATAAT AATAATGAGA

160 170 180 190 200

....|....| ....|....| ....|....| ....|....| ....|....|

*Can(DC-23)* ATTAATTAAA AATTA-GATT GAATTTGAGA CCAAGTTTTA TA--TCAATT

*DC-23* ATTAATTAAA A-TTAAGATT GAATTTGGGA CCAAGTTTTA TA--TCAATT

*Can(98-4)* CTTAATTAAA ATTTAAGATA AAATTTGAAA CCAAGTTTTA TA--TCAATT

*DC-98-4* CTTAATTAAA AATTAAACTA AAATTTGAAA CCAAGTTTTA TA--TCAATT

*Can*(41-5) CTTAATTAAA A-TTAAGATA AAATTTGAAA CCAAGTTTTA TA--TCAATT

*Tour(41-5)* ATTAATTAAA ATTTA-GCTT GAATTTGGGA CCAAGTTTTA TA--TCAATT

*Ogu*(41-5) CCTTATGGAA ATTTAAGGTT TTTTGGGAAC CCATTTGTTA AAAACAAATT

*DC-41-5* ATTAATTAAA A-TTAAGCTA GAATTTGAAA CCAAGTTTTA TA--TCAATT

210 220 230 240 250

....|....| ....|....| ....|....| ....|....| ....|....|

*Can(DC-23)* TTAAAAAACC TAAACCTCCT TTTTGCGCAA CC-CTCCTTA AAAAAAAATT

*DC-23* TTAAAAAACC TAAACCTCCT TTTTGCGCAA CC-CTCCTTA AAAAAAAATT

*Can(98-4)* TTAAAAAACC TAAACCTCCT TTTTGCGCAA CA-CTCCTTA AAAAAAAATT

*DC-98-4* TTAAAAAACC TAAACCTCCT TTTTGCGCAA CC-CTCCTTA AAAAAAAATT

*Can*(41-5) TTAAAAAACC TAAACCCCCT TTTTGCGCAA CCACCCCTTA AAAAAAAATT

*Tour(41-5)* TTAAAAAACC TAAACCTCCT TTTTGCGCAA CC-CTCCTTA AAAAAAAATT

*Ogu*(41-5) GGAAAACCCC TACCCCTCCT TTTTGGCCAA AA--GCCCTA AAAAAAAATT

*DC-41-5* TTAAAAAACC TAAACCTCCT TTTTGCGCAA CA-CTCCTTA AAAAAAAATT

260 270 280 290 300

....|....| ....|....| ....|....| ....|....| ....|....|

*Can(DC-23)* TCCATT-AAA CTAAAAAGAA T--AAGGGGA AGGAAGAAAG CGGATCGA-G

*DC-23* TCCATT-TAA CTAAAAAGAA T--AAGGGGA AGGAAGAAAG CGGATCGATG

*Can(98-4)* TCCATT-AAA CTAAAAAGAA TT-AAGGGGA AGGAAGAAAG CGAATCGA-G

*DC-98-4* TCCATTTAAA CTAAAAAAAA A--AAGGGGA AGGAAAAAAG CAAATCGATG

*Can*(41-5) TCCATTTAAA CTAAAAAAAA ATAAGGGGAA GGGAAAAAAG CAAACCGATG

*Tour(41-5)* TCCATT-TAA CTAAAAAGAA T--AAGGGGA AGGAAGAAAG CGGATCGATG

*Ogu*(41-5) TCCCTT-AAA CCAAAATTTT CT-TGGGGGA CGGAAAAACG -AAAACAAGG

*DC-41-5* TCCATT-AAA CTAAAAAGAA T--AAGGGGA AGGAAGAAAG CGAATCGATG

310 320 330 340 350

....|....| ....|....| ....|....| ....|....| ....|....|

*Can(DC-23)* GTGTTAATTC CCCATCCTCC AATTT-GTCC TTCCCCA-GG GTTGTTGTCT

*DC-23* -TGTTAATTC CCCATCCCCC AATTT-GTCC TTCCCCA-GG GTTGTTGTCT

*Can(98-4)* GTGTTAATTC CCCATCCCC- AAATTAGTCC TTCCCCA-GG GTCGTTGCTT

*DC-98-4* GTGTTAATTC CCCATCCTCC AAATTAGTCC CTTCCCAAGG GTCGTTGTCT

*Can*(41-5) GTGTTAATTC CCCATCCCCC AATTTAGTCC TTCCCCAGGG GTGGTTGTCC

*Tour(41-5)* -TGTTAATTC CCCATCCTCC AATTT-GTCC TTCCCCA-GG GTTGTTGTCT

*Ogu*(41-5) --GGAAATCC CCCCCCCCC- --TTTTTTTT TTTCCCGGGG GGGGAGGC--

*DC-41-5* -TGTTAATTC CCCATCCTC- AAATTAGTCC -TTCCCAAGG GTTGTTGTCT

360 370 380 390 400

....|....| ....|....| ....|....| ....|....| ....|....|

*Can(DC-23)* CAATGAATAA TTGTAGGAGT GAAATCTTGA T-TGAATAAA AAAAAATACG

*DC-23* CAATGAATAA TTGTAGGAGG GAAATCTTGA T-TGAATAAA AAAAAATACG

*Can(98-4)* CAATGAATAA TTGTAGGAGG GAAATCTTGA T-GGAATAAA AAAAACTACG

*DC-98-4* CAATGAAAAA TTGTAGGAGG GAAATCTTGA T-AAAATAAA AAAAACTACA

*Can*(41-5) CAATAAAAAA TTGAAGGAGG GAAATCTTGA TTAAAAAAAA AAAAACTACA

*Tour(41-5)* CAATGAATAA TTGTAGGAGT GAAATCTTGA T-TGAATAAA AAAAAATACG

*Ogu*(41_5) CAAAAAAAAA ----AGAAGT GAGCATTTTT TTTAAAAAAA GGCCTCGGAA

*DC-41-5* CAATGAATAA TTGTAGGAGT GAAATCTTGA T-AGAATAAA AAAAACTACG

410 420

....|....| ....|....| ....|....

*Can(DC-23)* AAAAAAAAAA TTCCTAATTT TATTATTTT

*DC-23* AAAAAAAAAA TTCCTAATTT TATTATTTC

*Can(98-4)* AAAAAAAAAA TTCCTAATTT TATTA-TTT

*DC-98-4* AAAAAAAAAA TTCCTAATTC TATTA-TTT

*Can*(41-5) AAAAAAAAAA TTCCTAATTT TATTA-TTT

*Tour(41-5)* AAAAAAAAAA TTCCTAATTT TATTATT--

*Ogu*(41-5) AAAAAAGCTA TTTTTTTTTT TCCGAATAC

*DC-41-5* AAAAAAAAAA TCCCTAATTT TATTAATTT

Fig.S4b. Alignment pattern of amplicon sequences obtained from primer atpF-atpH

10 20 30 40 50

....|....| ....|....| ....|....| ....|....| ....|....|

*Can*(DC-23) CCGTGGGATG AGAAGTTGCA -GAGTAGCTG ATAGTGGCCT CCCTCTCCGA

*DC-23* CCGTGGGATG AGAAGTTGCA -GAGTAGCTG ATAGTGGCCT CCCTCTCCGA

*Can(*98-4) AATCGGGATC ATAT-TGGCA CGAG-ACCTG ACAGTGGCCT GCCTCTCCGA

DC-98-4 CCGTGGGATG AGAAGTGGCA -GAGTAGCTG ATAGCGGCCT CCCTGTAAGA

*Can*(*41-5)* CCGCGGGATG ATAAGTGGCA CGAGTAGCTG ATAGTGGCCT CCCTCTCCGA

*Tour(41-5)* CCGTGGGATG AGAAGTTGCA -GAGTAGCTG ATAGTGGCCT CCCTCTCCGA

*Ogu*(*41-5)* CCGTGGGATG AGAAGTTGCA -GAGTAGCTG ATAGTGGCCT CCCTCTCCGA

*DC-41-5* CCGTGGGATG AGAAGTTGCA -GAGTAGCTG ATAGTGGCCT CCCTCTCCGA

60 70 80 90 100

....|....| ....|....| ....|....| ....|....| ....|....|

*Can*(DC-23) GTAAGAAATA CCTCTCCCAA GGGTCAAGTC ACTCCGTACC TTTTTAGTCA

*DC-23* GTAAGAAATA CCTCTCCCAA GGGTCAAGTC ACTCCGTACC TTTTTAGTCA

*Can(*98-4) GGAAAAAATA CCTCTCCCAA GGGGCAAGTC ACTCCGGATC TTATTTCTCA

DC-98-4 GGAAGAAATA CCTCTCCCAA GGGTCAAGTC ACTCCGTAGA TTTTAAGGCA

*Can*(*41-5)* GGAAAAAATA CCTCTCC--- ---------- ---------- ----------

*Tour(41-5)* GTAAGAAATA CCTCTCCCAA GGGTCAAGTC ACTCCGTACC TTTTTAGTCA

*Ogu*(*41-5)* GTAAGAAATA CCTCTCC--- ---------- ---------- ----------

*DC-41-5* GTAAGAAATA CCTCTCCCAA GGGTCAAGTC ACTCCGTACC TTTTTAGTCA

110 120 130 140 150

....|....| ....|....| ....|....| ....|....| ....|....|

*Can*(DC-23 AGTAAGAAAT ACCTCTCCTA ACATGACTTC GAGAATTGGG CGGAGCTGGA

*DC-23* AGTAAGAAAT ACCTCTCCTA ACATGACTTC GAGAATTGGG CGGAGCTGGA

*Can(*98-4) AATAAGAAAT ACCTCTCCCA AAATGATTTC GACAATTGGG T----CTGGA

DC-98-4 AGTAAGAAAT ACCTCTCCTA ACATGACTTC GAAAATTGGG CGGATCTGGA

*Can*(*41-5)* ---------- --------TA ACATGACTTC GAGAATTGGG CGGAGCTGGA

*Tour(41-5)* AGTAAGAAAT ACCTCTCCTA ACATGACTTC GAGAATTGGG CGGAGCTGGA

*Ogu*(*41-5)* ---------- --------TA ACATGACTTC GAGAATTGGG CGGAGCTGGA

*DC-41-5* AGTAAGAAAT ACCTCTCCTA ACATGACTTC GAGAATTGGG CGGAGCTGGA

160 170 180 190 200

....|....| ....|....| ....|....| ....|....| ....|....|

*Can*(DC-23) ACATTTGATG CAATAGCTGA CAA-TCCAAT GGGAATTATC CAATCGAAGG

*DC-23* ACATTTGATG CAATAGCTGA CAA-TCCAAT GGGAATTATC CAATCGAAGG

*Can(*98-4) CTGGTTCTCT TAGTCGTTG- CGTTAACAGT TGTGACTCCC CGAGCTAACT

DC-98-4 ACATTTGTTC CCTTTCCAAA AAAATCCCAT GGGACTTTTC CAATCTCTGG

*Can*(*41-5)* ACATTTGATG CAATAGCTGA CAA-TCCAAT GGGAATTATC CCATTGAAGG

*Tour(41-5)* ACATTTGATG CAATAGCTGA CAA-TCCAAT GGGAATTATC CAATCGAAGG

*Ogu*(*41-5)* ACATTTGATG CAATAGCTGA CAA-TCCAAT GGGAATTATC CAATCGAAGG

*DC-41-5* ACATTTGATG CAATAGCTGA CAA-TCCAAT GGGAATTATC CAATCGAAGG

210 220 230 240 250

....|....| ....|....| ....|....| ....|....| ....|....|

*Can*(DC-23) AAGAATGATT CA-TGACTAC T-CTTGCTGC TTTGAAAGAA --CTCTCTGA

*DC-23* AAGAATGATT CA-TGACTAC T-CTTGCTGC TTTGAAAGAA --CTCTCTGA

*Can(*98-4) AAGAAAGGTC AC--GACCCT C-CGAGGGGG GTTGGATGAC --CGATTGGA

DC-98-4 AATAATGGTT TC-TCCCTTC TCTTGGCTCC TTAT-AAC-A -CCCTGCTGA

*Can*(*41-5)* AATACCGATT CC-TGTTTAA C-CTTGCTGG TATGAAAGAA --CTCGG-GA

*Tour(41-5)* AAGAATGATT CA-TGACTAC T-CTTGCTGC TTTGAAAGAA --TTCTCTGA

*Ogu*(*41-5)* AAGAATGATT CA-TGACTAC T-CTTGCTGC TTTGAAAGAA --CTCTCTGA

*DC-41-5* AAGAATGATT CA-TGACTAC T-CTTGCTGC TTTGAAAGAA --CTCTCTGA

260 270 280 290 300

....|....| ....|....| ....|....| ....|....| ....|....|

*Can*(DC-23) GC--TTGGTT TTC-AGTCAA CTTTAT--CT CTCTCTCTTT -ATGAGCGGT

*DC-23* GC--TTGGTT TTC-AGTCAA CTTTAT--CT CTCTCTCTTT -ATGAGCGGT

*Can(*98-4) GT--TGGGAT CCCTTATTGA TTCTCT--CT CTCTTTTGAC -GCGGGC---

DC-98-4 TG-TTGGGTT TTC--TCCCA TTTTATTTC- CTCTCCCTTA --AG-GG-GC

*Can*(*41-5)* G---TTAGTT TT--AGCCCC CTCTCA-CCC TTCCCCCAT- -AAGAAC---

*Tour(41-5)* GC--TTGGTT TTC-AGTCAA CTTTAT--CT CTCTCTCTTT -ATGAGCGGT

*Ogu*(*41-5)* GC--TTGGTT TTC-AGTCAA CTTTAT--CT CTCTCTCTTT -ATGAGCGGT

*DC-41-5* GC--TTGGTT TTC-AGTCAA CTTTAT--CT CTCTCTCTTT -ATGAGCGGT

310 320 330 340 350

....|....| ....|....| ....|....| ....|....| ....|....|

*Can*(DC-23) GGTCATGCTC TG--TACAT- -TCTAGTATA TGTTGATGAT CTTAGAATC-

*DC-23* GGTCATGCTC TG--TACAT- -TCTAGTATA TGTTGATGAT CTTAGAATC-

*Can(*98-4) --TCGGGCCT TGGGAGAAGA ----ATGAAT TGCTTTTCTA CCCAGAAAT-

DC-98-4 GCACATACCC CT-TGGCAG- --AGGTATTA T-TT--TG-T CG--GAC-C-

*Can*(*41-5)* ---C---C-C AA--AAAATG -TC-A-AACA -GT-AA--AA C--AAAATC-

*Tour(41-5)* GGTCATGCTC TG--TACAT- -TCTAGTATA TGTTGATGAT CTTAGAATC-

*Ogu*(*41-5)* GGTCATGCTC TG--TACAT- -TCTAGTATA TGTTAATGAT CTTAGAATC-

*DC-41-5* GGTCATGCTC TG--TACAT- -TCTAGTATA TGTTGATGAT CTTAGAATC-

360 370 380 390 400

....|....| ....|....| ....|....| ....|....| ....|....|

*Can*(DC-23) --ACATGGTC CAATCCTCTG AATATTGATG ACATAGTATG CA--TCAA--

*DC-23* --ACATGGTC CAATCCTCTG AATATTGATG ACATAGTATG CA--TCAA--

*Can(*98-4) --AAACCGGA TTCTCTCCTC GATATTAAGC AGCGAGAAGG CATTTCACCT

DC-98-4 -TGCC-GGCC ATCA-TGGGC TCCTCTGCTA T-TTCTCCTG CC--ACGA--

*Can*(*41-5)* --AAAAAACA AACACACCT- -AT--TG-CA AAAATAAATA AA--ACAAG-

*Tour(41-5)* --ACATGGTC CAATCCTCTG AATATTGATG ACATAGTATG CA--TCAA--

*Ogu*(*41-5)* --ACATGGTC CAATCCTCTG AATATTGATG ACATAGTATG CA--TCAA--

*DC-41-5* --ACATGGTC CAATCCTCTG AATATTGATG ACATAGTATG CA--TCAA--

410 420 430 440

....|....| ....|....| ....|....| ....|....| ....|.

*Can*(DC-23) --AAATGCC- ATCTTT-TCT ATAAAGTATC TGGCTCAATC TTGGTA

*DC-23* --AAATGCC- ATCTT-TTCT ATAAAGTATC TGGCTCAATC TTGGTA

*Can(*98-4) TTGCCCCCAA CTCTATAATC TTTCACTCGA TACCGAAGCC GTGGCC

DC-98-4 GTAGGCGTTT C-TCTTTTCC CGCTCTTTTT TTTTTTTTTT TCTGCC

*Can*(*41-5)* CTAAA-GAGG TCGTTTATCA AACGAGCTTT TCCTTTCATC CTCCGC

*Tour(41-5)* --AAATGCC- ATCTTT-TCT ATAAAGTATC TGGCTCAATC TTGGT-

*Ogu*(*41-5)* --AAATGCC- ATCTTT-TCT ATAAAGTATC TGGCTCAATC TTGGTA

*DC-41-5* --AAATGCC- ATCTTT-TCT ATAAAGTATC TGGCTAAATC TTGGTA

Fig.S4c. Alignment pattern of amplicon sequences obtained from primer Primer P16

10 20 30 40 50

....|....| ....|....| ....|....| ....|....| ....|....|

*Can*(DC-23) ATTCAAAGCT GG-TGTTAAA GAGTATAAAT TGAA--TTAT TATACTCCTG

*DC-23* ATTCAAAGCT GG-TGTTAAA GAGTATAAAT TGAA--TTAT TATACTCCTG

*Can(*98-4) ATTCAAAGCT GG-TGTTAAA GAGTATAAAT TGAA--TTAT TATACTCCTG

*DC-98-4* ACCCATAAAT GG--G--AAG GCGT-CA-AT TATC--TCAT TCTTCTTTTG

*Can*(*41-5)* ATTGGCATTT GCCTACTACC GCGCTCCCGC TAAAACTCGG AGTTCTACGG

*Tour(41-5)* ATTCAAAGCT GG-TGTTAAA GAGTATAAAT TGAA--TTAT TATACTCCTG

*Ogu*(*41-5)* ATTCAAAGCT GG-TGTTAAA GAGTATAAAT TGAA--TTAT TATACTCCTG

*DC-41-5* ATTCAAAGCT GG-TGTTAAA GAGTATAAAT TGAA--TTAT TATACTCCTG

60 70 80 90 100

....|....| ....|....| ....|....| ....|....| ....|....|

*Can*(DC-23) --AATATGAA ACCAAGGATA CTGATATCTT GGCAGCATTC CGA--GTAAC

*DC-23* --AATATGAA ACCAAGGATA CTGATATCTT GGCAGCATTC CGA--GTAAC

*Can(*98-4) --AATATGAA ACCAAGGATA CTGATATCTT GGCAGCATTC CGA--GTAAC

*DC-98-4* GTAAA-TCAA GTCCACCACG TAGACATTTC ATAAACTGCT CTACCATAGT

*Can*(*41-5)* --TTTCTAAA GGTGGGGCCA AAAAGAGTCG GGAAAAAAGG CAA----AAC

*Tour(41-5)* --AATATGAA ACCAAGGATA CTGATATCTT GGCAGCATTC CGA--GTAAC

*Ogu*(*41-5)* --AATATGAA ACCAAGGATA CTGATATCTT GGCAGCATTC CGA--GTAAC

*DC-41-5* --AATATGAA ACCAAGGATA CTGATATCTT GGCAGCATTC CGA--GTAAC

110 120 130 140 150

....|....| ....|....| ....|....| ....|....| ....|....|

*Can*(DC-23) TCCTC----- -AACCCGGAG TTCCAC-CTG AAG------- -AAGCAGGGG

*DC-23* TCCTC----- -AACCCGGAG TTCCAC-CTG AAG------- -AAGCAGGGG

*Can(*98-4) TCCTC----- -AACCCGGAG TTCCAC-CTG AAG------- -AAGCAGGGG

*DC-98-4* TCTTCGCGGA TAACCCCAAC TTAGGTTTAA TAGTACATCC TAATAGGGGA

*Can*(*41-5)* TCCTA----- -AACGCCCA- --CCACACGG GGG------- -AAGGCGGGG

*Tour(41-5)* TCCTC----- -AACCCGGAG TTCCAC-CTG AAG------- -AAGCAGGGG

*Ogu*(*41-5)* TCCTC----- -AACCCGGAG TTCCAC-CTG AAG------- -AAGCAGGGG

*DC-41-5* TCCTC----- -AACCCGGAG TTCCAC-CTG AAG------- -AAGCAGGGG

160 170 180 190 200

....|....| ....|....| ....|....| ....|....| ....|....|

*Can*(DC-23) CTGCGGTAGC TGCTGAATCT -TCTACTGGT ACATGGACA- -ACTGTGTGG

*DC-23* CTGCGGTAGC TGCTGAATCT -TCTACTGGT ACATGGACA- -ACTGTGTGG

*Can(*98-4) CTGCGGTAGC TGCTGAATCT -TCTACTGGT ACATGGACA- -ACTGTGTGG

*DC-98-4* CGTCCATACT TGTTCAATTT ATCTCTTTCA ACTTGGATAC CATGAGGTGG

*Can*(*41-5)* TTGCGCAACT TGTTG--TTC -AACACTGGA ACATGGGCA- -ACGGTGTGA

*Tour(41-5)* CTGCGGTAGC TGCTGAATCT -TCTACTGGT ACATGGACA- -ACTGTGTGG

*Ogu*(*41-5)* CTGCGGTAGC TGCTGAATCT -TCTACTGGT ACATGGACA- -ACTGTGTGG

*DC-41-5* CTGCGGTAGC TGCTGAATCT -TCTACTGGT ACATGGACA- -ACTGTGTGG

210 220 230 240 250

....|....| ....|....| ....|....| ....|....| ....|....|

*Can(DC-23)* ACC---GATG GGCTTAC--C AGCCTTGACC GTTACAAAGG ACGATGCTAC

*DC-23* ACC---GATG GGCTTAC--C AGCCTTGACC GTTACAAAGG ACGATGCTAC

*Can(*98-4) ACC---GATG GGCTTAC--C AGCCTTGACC GTTACAAAGG ACGATGCTAC

*DC-98-4* TCCCTGGAAA GTTTTAGTAT AAGCCGGAGG GATTCGCAGA TCCTCTAGAC

*Can*(*41-5)* A-----AAAA GTTTTACTAC AGGCTGGAGG GATACAAAGA ACCATGATAC

*Tour(41-5)* ACC---GATG GGCTTAC--C AGCCTTGACC GTTACAAAGG ACGATGCTAC

*Ogu*(*41-5)* ACC---GATG GGCTTAC--C AGCCTTGACC GTTACAAAGG ACGATGCTAC

*DC-41-5* ACC---GATG GGCTTAC--C AGCCTTGACC GTTACAAAGG ACGATGCTAC

260 270 280 290 300

....|....| ....|....| ....|....| ....|....| ....|....|

*Can(DC-23)* CACATCGAGC CCGTTCCAGG AGAAGAAACT CAATTT---- -ATTGCG-TA

*DC-23* CACATCGAGC CCGTTCCAGG AGAAGAAACT CAATTT---- -ATTGCG-TA

*Can(*98-4) CACATCGAGC CCGTTCCAGG AGAAGAAACT CAATTT---- -ATTGCG-TA

*DC-98-4* GTAAAGCAGC CAGGGCTTTG AACCCAAATA CGTTACCCAC AATTGAGGTA

*Can*(*41-5)* CACAAGCAGC CCGGTCTTGG AAAAAAAATA CGTTTC---- -CCTGAG-TA

*Tour(41-5)* CACATCGAGC CCGTTCCAGG AGAAGAAACT CAATTT---- -ATTGCG-TA

*Ogu*(*41-5)* CACATCGAGC CCGTTCCAGG AGAAGAAACT CAATTT---- -ATTGCG-TA

*DC-41-5* CACATCGAGC CCGTTCCAGG AGAAGAAACT CAATTT---- -ATTGCG-TA

310 320 330 340 350

....|....| ....|....| ....|....| ....|....| ....|....|

*Can(DC-23)* TGTAGCTTAC CCATTAGACC TTTTTGAAGA AGGGTCTGTT ACTAACATGT

*DC-23* TGTAGCTTAC CCATTAGACC TTTTTGAAGA AGGGTCTGTT ACTAACATGT

*Can(*98-4) TGTAGCTTAC CCATTAGACC TTTTTGAAGA AGGGTCTGTT ACTAACATGT

*DC-98-4* AACATGTTAG TAACAGACCC TTCTTCAAAA AGGTCTAATG GGTAAGCTAC

*Can*(*41-5)* GGGGAAATAC TGTTAAAAAC TTATCCTACA AGGAAATGGT AATAGGGTGA

*Tour(41-5)* TGTAGCTTAC CCATTAGACC TTTTTGAAGA AGGGTCTGTT ACTAACATGT

*Ogu*(*41-5)* TGTAGCTTAC CCATTAGACC TTTTTGAAGA AGGGTCTGTT ACTAACATGT

*DC-41-5* TGTAGCTTAC CCATTAGACC TTTTTGAAGA AGGGTCTGTT ACTAACATGT

360 370 380 390 400

....|....| ....|....| ....|....| ....|....| ....|....|

*Can(DC-23)* TTACCTCAAT TGTGGGTAAC GTATTTGGGT TCAAAGCCCT GGCTG-CTCT

*DC-23* TTACCTCAAT TGTGGGTAAC GTATTTGGGT TCAAAGCCCT GGCTG-CTCT

*Can-(*98-4) TTACCTCAAT TGTGGGTAAC GTATTTGGGT TCAAAGCCCT GGCTG-CTCT

*DC 98-4* ATACG-CAAT -----AAATT GAGTTTCTTC TCCTGGAACG GGCTCGATGT

*Can*(*41-5)* ATCCCTCTAT GGAAGAAATC GATTTTGTTC TCCGGGACCG GGCTGGTTTT

*Tour(41-5)* TTACCTCAAT TGTGGGTAAC GTATTTGGGT TCAAAGCCCT GGCTG-CTCT

*Ogu*(*41-5)* TTACCTCAAT TGTGGGTAAC GTATTTGGGT TCAAAGCCCT GGCTG-CTCT

*DC-41-5* TTACCTCAAT TGTGGGTAAC GTATTTGGGT TCAAAGCCCT GGCTG-CTCT

410 420 430 440 450

....|....| ....|....| ....|....| ....|....| ....|....|

*Can(DC-23)* ACGTCTAGAG GATCTGCGAA -TCCCTCCGG CTTATACTAA AAC----TTT

*DC-23* ACGTCTAGAG GATCTGCGAA -TCCCTCCGG CTTATACTAA AAC----TTT

*Can(*98-4) ACGTCTAGAG GATCTGCGAA -TCCCTCCGG CTTATACTAA AAC----TTT

*DC-98-4* G-GTAGCATC GTCCTTTGTA ACGGTCAAGG CTGGTAAGCC CAT---CGGT

*Can*(*41-5)* AGGTCAAAAG GATCTTTGAA ACCCCTCCGG GGTTGAAAAA AACAAATTTG

*Tour(41-5)* ACGTCTAGAG GATCTGCGAA -TCCCTCCGG CTTATACTAA AAC----TTT

*Ogu*(*41-5)* ACGTCTAGAG GATCTGCGAA -TCCCTCCGG CTTATACTAA AAC----TTT

*DC-41-5* ACGTCTAGAG GATCTGCGAA -TCCCTCCGG CTTATACTAA AAC----TTT

460 470 480 490 500

....|....| ....|....| ....|....| ....|....| ....|....|

*Can(DC-23)* CCAGGGACCA CCTCATGG-T ATCCAAGTTG AAAGAGATAA A-TTGAACAA

*DC-23* CCAGGGACCA CCTCATGG-T ATCCAAGTTG AAAGAGATAA A-TTGAACAA

*Can(*98-4) CCAGGGACCA CCTCATGG-T ATCCAAGTTG AAAGAGATAA A-TTGAACAA

*DC-98-4* CCACACAGTT GTCCATG--T ACC--AGTAG AAGATTCAGC AGCTACCGCA

*Can*(*41-5)* CCGGGGACCT TCTTCCGGGT ACCCAAAAAA AAAAAAAAAA AATTCCGCCA

*Tour(41-5)* CCAGGGACCA CCTCATGG-T ATCCAAGTTG AAAGAGATAA A-TTGAACAA

*Ogu*(*41-5)* CCAGGGACCA CCTCATGG-T ATCCAAGTTG AAAGAGATAA A-TTGAACAA

*DC-41-5* CCAGGGACCA CCTCATGG-T ATCCAAGTTG AAAGAGATAA A-TTGAACAA

510 520 530 540 550

....|....| ....|....| ....|....| ....|....| ....|....|

*Can(DC-23)* GTATGGACGT C-CCCTATTA GGA-TGTACT ATTAAACCTA AGTTGGGGTT

*DC-23* GTATGGACGT C-CCCTATTA GGA-TGTACT ATTAAACCTA AGTTGGGGTT

*Can(*98-4) GTATGGACGT C-CCCTATTA GGA-TGTACT ATTAAACCTA AGTTGGGGTT

*DC-98-4* GCCCCTGCTT C-TTCAGGTG GAACTCCGGG TTGAGGAGTT ACTCGGAATG

*Can*(*41-5)* GCCCCGGCTT CTCCCTATTG GAAATGTCGG TTTAAAGATT TATTGGGGAT

*Tour(41-5)* GTATGGACGT C-CCCTATTA GGA-TGTACT ATTAAACCTA AGTTGGGGTT

*Ogu*(*41-5)* GTATGGACGT C-CCCTATTA GGA-TGTACT ATTAAACCTA AGTTGGGGTT

*DC-41-5* GTATGGACGT C-CCCTATTA GGA-TGTACT ATTAAACCTA AGTTGGGGTT

560 570 580 590 600

....|....| ....|....| ....|....| ....|....| ....|....|

*Can(DC-23)* AT--CCGCGA AGAACTATGG TAGAGCAGTT TATGAATGTC TACGTGGTGG

*DC-23* AT--CCGCGA AGAACTATGG TAGAGCAGTT TATGAATGTC TACGTGGTGG

*Can(*98-4) AT--CCGCGA AGAACTATGG TAGAGCAGTT TATGAATGTC TACGTGGTGG

*DC-98-4* CTGCCAAGAT ATCAGTATCC TTGGTTTCAT ATTCAGGAGT ATAATAATTC

*Can*(*41-5)* GCTCCCCCAA AAAAATATGG CCTAGGTTTT TTTTAAGGGG GACAAGGGGG

*Tour(41-5)* AT--CCGCGA AGAACTATGG TAGAGCAGTT TATGAATGTC TACGTGGTGG

*Ogu*(*41-5)* AT--CCGCGA AGAACTATGG TAGAGCAGTT TATGAATGTC TACGTGGTGG

*DC-41-5* AT--CCGCGA AGAACTATGG TAGAGCAGTT TATGAATGTC TACGTGGTGG

610 620 630 640 650

....|....| ....|....| ....|....| ....|....| ....|....|

*Can(DC-23)* AC--TTGATT TTACCAAAGA TGATGAGAAT GTGAACTCTC AACCA--TTT

*DC-23* AC--TTGATT TTACCAAAGA TGATGAGAAT GTGAACTCTC AACCA--TTT

*Can(*98-4) AC--TTGATT TTACCAAAGA TGATGAGAAT GTGAACTCTC AACCA--TTT

*DC-98-4* AATTTATACT CTTTAACACC AGCTTTGAAT CCAAC-ACTT GCTTTAGTCT

*Can*(*41-5)* GTCATTTTTT TCCCCCTAAA CGCCGGGGAT TGGGACCCCC CCCCCCCTTT

*Tour(41-5)* AC--TTGATT TTACCAAAGA TGATGAGAAT GTGAACTCTC AACCA--TTT

*Ogu*(*41-5)* AC--TTGATT TTACCAAAGA TGATGAGAAT GTGAACTCTC AACCA--TTT

*DC-41-5* AC--TTGATT TTACCAAAGA TGATGAGAAT GTGAACTCTC AACCA--TTT

660

....|....| ...

*Can(DC-23)* ATGGGTTGGA GAG

*DC-23* ATGGGTTGGA GAG

*Can(*98-4) ATGGGTTGGA GAG

*DC-98-4* CT--GTTGGG GGG

*Can*(*41-5)* TTGGGGGGGG GGG

*Tour(41-5)* ATGGGTTGGA GAG

*Ogu*(*41-5)* ATGGGTTGGA GAG

*DC-41-5* ATGGGTTGGA GAG

Fig.S4d. Alignment pattern of amplicon sequences obtained from primer Primer rbeL

Primer trnL

10 20 30 40 50

....|....| ....|....| ....|....| ....|....| ....|....|

*Can(DC-23)* ACTTGAACCC TCA--CGATT TTAAAAGTCA ACGGATTTTC ATCTTACTAT

*DC-23* ACTTGAACCC TCA--CGATT TTAAAAGTCA ACGGATTTTC ATCTTACTAT

*Can(*98-4) ACTTGAACCC TCA--CGATT TTAAAAGTCA ACGGATTTTC ATCTTACTAT

*DC-98-4* ACTTGAACCC TCA--CGATT TTAAAAGTCA ACGGATTTTC ATCTTACTAT

*Can*(*41-5)* ACTTGAACCC TCGGCCGAGC C-AACAGTGA A-GAAATTTC A-CTTTC-A-

*Tour(41-5)* ACTTGAACCC TCA--CGATT TTAAAAGTCA ACGGATTTTC ATCTTACTAT

*Ogu*(*41-5)* ACTTGAACCC TCA--CGATT TTAAAAGTCA ACGGATTTTC ATCTTACTAT

*DC-41-5* ACTTGAACCC TCA--CGATT TTAAAAGTCA ACGGATTTTC ATCTTACTAT

60 70 80 90 100

....|....| ....|....| ....|....| ....|....| ....|....|

*Can(DC-23)* AAATTTCAT- TGTTGTCAGT ATT-GACAT- GTAGAATGGG ACTCTATCTT

*DC-23* AAATTTCAT- TGTTGTCAGT ATT-GACAT- GTAGAATGGG ACTCTATCTT

*Can(*98-4) AAATTTCAT- TGTTGTCAGT ATT-GACAT- GTAGAATGGG ACTCTATCTT

*DC-98-4* AAATTTCAT- TGTTGTCAGT ATT-GACAT- GTAGAATGGG ACTCTATCTT

*Can*(*41-5)* ATATT-CATA TGTTGTCAGT ATTTGACATT GTAGAATGGG ACTCTATCTT

*Tour(41-5)* AAATTTCAT- TGTTGTCAGT ATT-GACAT- GTAGAATGGG ACTCTATCTT

*Ogu*(*41-5)* AAATTTCAT- TGTTGTCAGT ATT-GACAT- GTAGAATGGG ACTCTATCTT

*DC-41-5* AAATTTCAT- TGTTGTCAGT ATT-GACAT- GTAGAATGGG ACTCTATCTT

110 120 130 140 150

....|....| ....|....| ....|....| ....|....| ....|....|

*Can(DC-23)* TATTCTCGTC CGATTAATAA GTTCCACCAA GGATCTATCA GACTATGAAG

*DC-23* TATTCTCGTC CGATTAATAA GTTCCACCAA GGATCTATCA GACTATGAAG

*Can(*98-4) TATTCTCGTC CGATTAATAA GTTCCACCAA GGATCTATCA GACTATGAAG

*DC-98-4* TATTCTCGTC CGATTAATAA GTTCCACCAA GGATCTATCA GACTATGAAG

*Can*(*41-5)* TATTCTCGTC CGATTAATAA GTTCCACCAA GGATCTATCA GACTATGAAG

*Tour(41-5)* TATTCTCGTC CGATTAATAA GTTCCACCAA GGATCTATCA GACTATGAAG

*Ogu*(*41-5)* TATTCTCGTC CGATTAATAA GTTCCACCAA GGATCTATCA GACTATGAAG

*DC-41-5* TATTCTCGTC CGATTAATAA GTTCCACCAA GGATCTATCA GACTATGAAG

160 170 180 190 200

....|....| ....|....| ....|....| ....|....| ....|....|

*Can(DC-23)* TGAATCGTTT GATTCAACAC AAGGGATTGA ACTCCATTTG TTAGAACAGC

*DC-23* TGAATCGTTT GATTCAACAC AAGGGATTGA ACTCCATTTG TTAGAACAGC

*Can(*98-4) TGAATCGTTT GATTCAACAC AAGGGATTGA ACTCCATTTG TTAGAACAGC

*DC-98-4* TGAATCGTTT GATTCAACAC AAGGGATTGA ACTCCATTTG TTAGAACAGC

*Can*(*41-5)* TGAATCGTTT GATTCAACAC AAGGGATTGA ACTCCATTTG TTAGAACAGC

*Tour(41-5)* TGAATCGTTT GATTCAACAC AAGGGATTGA ACTCCATTTG TTAGAACAGC

*Ogu*(*41-5)* TGAATCGTTT GATTCAACAC AAGGGATTGA ACTCCATTTG TTAGAACAGC

*DC-41-5* TGAATCGTTT GATTCAACAC AAGGGATTGA ACTCCATTTG TTAGAACAGC

210 220 230 240 250

....|....| ....|....| ....|....| ....|....| ....|....|

*Can(DC-23)* TTCCATTGAG TCTCTGCACC TATCCCGCTT TCTAAACTCT GTTTTGTTCG

*DC-23* TTCCATTGAG TCTCTGCACC TATCCCGCTT TCTAAACTCT GTTTTGTTCG

*Can(*98-4) TTCCATTGAG TCTCTGCACC TATCCCGCTT TCTAAACTCT GTTTTGTTCG

*DC-98-4* TTCCATTGAG TCTCTGCACC TATCCCGCTT TCTAAACTCT GTTTTGTTCG

*Can*(*41-5)* TTCCATTGAG TCTCTGCACC TATCCCGCTT TCTAAACTCT GTTTTGTTCG

*Tour(41-5)* TTCCATTGAG TCTCTGCACC TATCCCGCTT TCTAAACTCT GTTTTGTTCG

*Ogu*(*41-5)* TTCCATTGAG TCTCTGCACC TATCCCGCTT TCTAAACTCT GTTTTGTTCG

*DC-41-5* TTCCATTGAG TCTCTGCACC TATCCCGCTT TCTAAACTCT GTTTTGTTCG

260 270 280 290 300

....|....| ....|....| ....|....| ....|....| ....|....|

*Can(DC-23)* CGTAACCCAG GATTTGGCTC AGGATTGCCC ATTGTTAATT CCAGGGTTTC

*DC-23* CGTAACCCAG GATTTGGCTC AGGATTGCCC ATTGTTAATT CCAGGGTTTC

*Can(*98-4) CGTAACCCAG GATTTGGCTC AGGATTGCCC ATTGTTAATT CCAGGGTTTC

*DC-98-4* CGTAACCCAG GATTTGGCTC AGGATTGCCC ATTGTTAATT CCAGGGTTTC

*Can*(*41-5)* CGTAACCCAG GATTTGGCTC AGGATTGCCC ATTGTTAATT CCAGGGTTTC

*Tour(41-5)* CGTAACCCAG GATTTGGCTC AGGATTGCCC ATTGTTAATT CCAGGGTTTC

*Ogu*(*41-5)* CGTAACCCAG GATTTGGCTC AGGATTGCCC ATTGTTAATT CCAGGGTTTC

*DC-41-5* CGTAACCCAG GATTTGGCTC AGGATTGCCC ATTGTTAATT CCAGGGTTTC

310 320 330 340 350

....|....| ....|....| ....|....| ....|....| ....|....|

*Can(DC-23)* TCTGAATTTG AAAGTTATCA CTTAGTAGGT TTCCATACCA A-GGCT-CAA

*DC-23* TCTGAATTTG AAAGTTATCA CTTAGTAGGT TTCCATACCA A-GGCT-CAA

*Can(*98-4) TCTGAATTTG AAAGTTATCA CTTAGTAGGT TTCCATACCA A-GGCT-CAA

*DC-98-4* TCTGAATTTG AAAGTTATCA CTTAGTAGGT TTCCATACCA A-GGCT-CAA

*Can*(*41-5)* TCTGAATTTG AAAGTTATCA CTTAGTAGGT TTCCATACCA A-GGCT-CAA

*Tour(41-5)* TCTGAATTTG AAAGTTATCA CTTAGTAGGT TTCCATACCA A-GGCT-CAA

*Ogu*(*41-5)* TCTGAATTTG AAAGTTATCA CTTAGTAGGT TTCCATACCA A-GGCT-CAA

*DC-41-5* TCTGAATTTG AAAGTTATCA CTTAGTAGGT TTCCATACCA A-GGCT-CAA

360

....|....| ....|....

*Can(DC-23)* TCCAATTAAG TCCGTAGCG

*DC-23* TCCAATTAAG TCCGTAGCG

*Can(*98-4) TCCAATTAAG TCCGTAGCG

*DC-98-4* TCCAATTAAG TCCGTAGCG

*Can*(*41-5)* TCCAATTAAG TCCGTAGCG

*Tour(41-5)* TCCAATTAAG TCCGTAGCG

*Ogu*(*41-5)* TCCAATTAAG TCCGTAGCG

*DC-41-5* TCCAATTAAG TCCGTAGCG

Fig.S4e. Alignment pattern of amplicon sequences obtained from primer Primer trnL
